# Dogs, cats, parasites, and humans in Brazil: opening the black box

**DOI:** 10.1186/1756-3305-7-22

**Published:** 2014-01-14

**Authors:** Filipe Dantas-Torres, Domenico Otranto

**Affiliations:** 1 Department of Immunology, Aggeu Magalhães Research Centre, Oswaldo Cruz Foundation, Recife Pernambuco 50670420, Brazil; 2 Department of Veterinary Medicine, University of Bari, Valenzano 70010, Bari, Italy

**Keywords:** Dogs, Cats, Humans, Zoonosis, Control, South America

## Abstract

Dogs and cats in Brazil serve as primary hosts for a considerable number of parasites, which may affect their health and wellbeing. These may include endoparasites (e.g., protozoa, cestodes, trematodes, and nematodes) and ectoparasites (i.e., fleas, lice, mites, and ticks). While some dog and cat parasites are highly host-specific (e.g., *Aelurostrongylus abstrusus* and *Felicola subrostratus* for cats, and *Angiostrongylus vasorum* and *Trichodectes canis* for dogs), others may easily switch to other hosts, including humans. In fact, several dog and cat parasites (e.g., *Toxoplasma gondii*, *Dipylidium caninum*, *Ancylostoma caninum*, *Strongyloides stercoralis*, and *Toxocara canis*) are important not only from a veterinary perspective but also from a medical standpoint. In addition, some of them (e.g., *Lynxacarus radovskyi* on cats and *Rangelia vitalii* in dogs) are little known to most veterinary practitioners working in Brazil. This article is a compendium on dog and cat parasites in Brazil and a call for a One Health approach towards a better management of some of these parasites, which may potentially affect humans. Practical aspects related to the diagnosis, treatment, and control of parasitic diseases of dogs and cats in Brazil are discussed.

## Portuguese version

Please see Additional file 1 (http://www.biomedcentral.com/content/supplementary/1756-3305-7-22-S1.pdf) for the Portuguese version of this review article.

## Introduction

The word “parasite” (from Ancient Greek, *parasitos*: *para* = beside, *sitos* = food) means literally a person that eats at the table of another. In the figurative sense, corrupt politicians that make the use their political power and public resources for their own benefit may be called parasites of society. But in parasitology, a parasite is an organism that takes benefit from another (the host), without giving something back and usually causing some damage to it. Incidentally, parasites constitute a diverse group of organisms that may affect a wide range of animal hosts, including amphibians, birds, fishes, mammals, and reptiles. They may be generally subdivided as endoparasites (=internal) and ectoparasites (=external), according to their location in the host. Ectoparasites may also be classified as permanent (e.g., lice and mites) or non-permanent (e.g., ticks and mosquitoes), depending on the relationship with their host; i.e., whether their life cycle takes place solely on their hosts or also in the environment.

**Figure 1:**
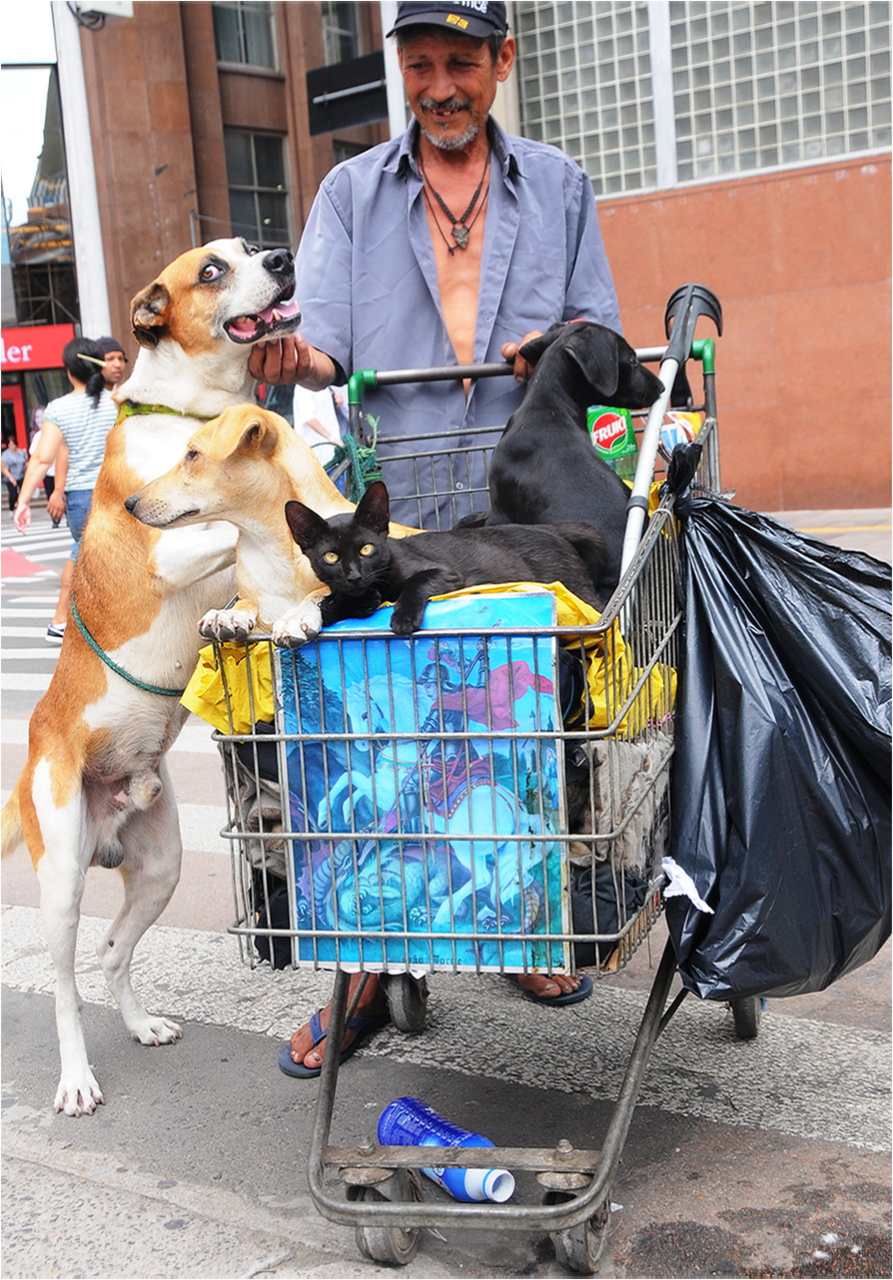
**Human**-**animal bond.** A homeless with his inseparable friends that were found abandoned in the streets of Porto Alegre, southern Brazil.

Dogs and cats are the most popular pet animals worldwide. Nowadays, dogs for example have assumed many functions as guides for blind people, as therapeutic agents, as guard and hunting dogs. Moreover, in many developing countries, both dogs and cats have become part of human families, regardless of the social class (Figure 1). The benefits of having a pet animal are undisputed [1], but both dogs and cats may harbour many parasites potentially transmissible to humans, which may represent a health risk, especially to children, the elderly and the immunocompromised [2]. For instance, *Toxoplasma gondii*, *Dipylidium caninum*, *Echinococcus granulosus*, *Ancylostoma braziliense*, *Toxocara canis*, *Onchocerca lupi*, and *Thelazia callipaeda* are common parasites of dogs and cats that can affect humans in different countries around the world. This fact emphasizes the need for a One Health approach towards a more efficient management and control of these parasites [3,4].

**Figure 2:**
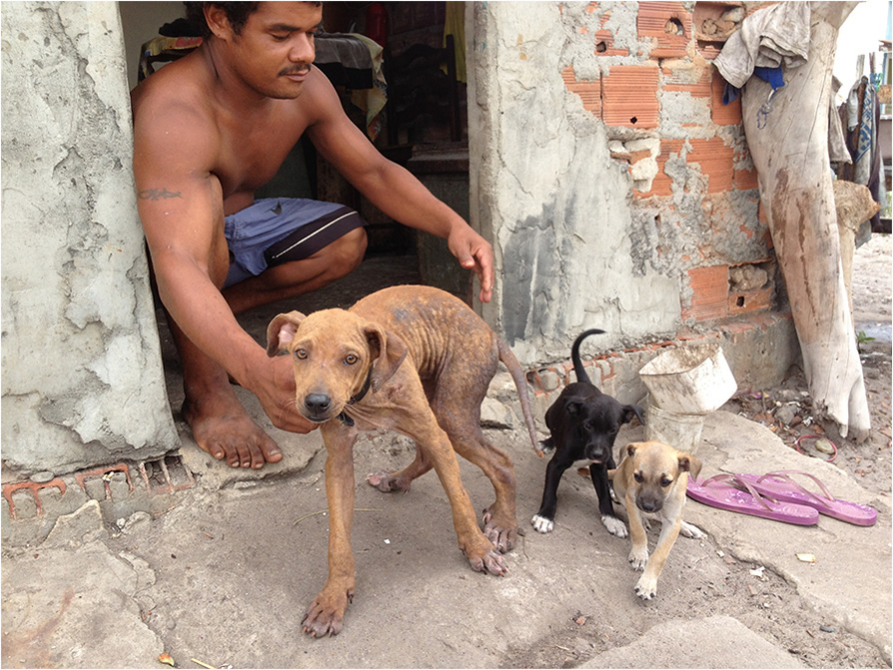
**Poor living conditions.** An owner and his dogs living in a poor rural community in Goiana, north-eastern Brazil, where visceral leishmaniosis is endemic. In poor rural communities people (as this man in the picture) are used to walking barefoot, which is a risk factor for bare feet-related diseases, such as cutaneous *larva migrans* and tungiasis.

At the beginning of the 21st century, parasitic diseases are still causing a severe burden on animal and human populations in tropical and subtropical regions around the world [5-7]. Furthermore, the impact of some of these diseases is disproportionally higher in developing countries such as Brazil, because the living conditions of the populations often favour the exposure to certain parasites, whose transmission may be associated with poor housing and sanitary conditions (Figure 2), as well as with inequities in the access to education and primary healthcare services.

Brazil is a country with continental dimensions (i.e., 8,515,767 km^2^), occupying most of the land area of South America and representing the largest economy in the region. Despite the persistent social and economic inequalities (e.g., unequal access to education, health care and sanitation, safe drinking water and decent housing), which have become the dark side of the Brazilian history, the importance of dogs and cats as pets, as well as the demand for high standard veterinary services, has increased over the last decades in Brazil. Impressively, according to the *Associação Brasileira da Indústria de Produtos para Animais de Estimação* (Abinpet), Brazil currently holds the world’s second largest dog and cat populations, with over 37 million dogs and 21 million cats (http://abinpet.org.br/imprensa/mercado-pet-deve-faturar-r-154-bilhoes-em-2013). Apropos, the growing number of dogs and cats in urban areas in Brazil has been accompanied by substantial increases in the animal feed and pet care markets. According to Abinpet, the Brazilian pet market will earn 15.4 billion Brazilian real (approximately 7 billion US dollars) in 2013, with an overall increase of 8.3% in comparison with 2012. Specifically, it has been estimated that in 2013 the pet service sector (services related to animal care, including bath and hair cut) will increase in 24.5%, pet food (animal feed) in 4.9%, pet care (equipments, accessories and products for animal hygiene and care) in 5.2%, and pet vet (medicines) in 6.7%. These numbers pose Brazil as the world’s second largest pet market, behind only the United States.

The diversity of natural environments present in Brazil is outstanding, as is its biodiversity. By the bye, dogs and cats living in Brazil may harbour a long list of parasites that may affect their wellbeing and eventually pose a risk to the health of their owners [8,9]. Information on their distribution, epidemiology, and impact on human health is fragmentary and mainly published in Brazilian journals. In this sense, this article is a compendium on dog and cat parasites in Brazil and a call for a One Health approach towards a better management of some of these parasites, which may potentially affect humans. Practical aspects related to the diagnosis, treatment, and control of parasitic diseases of dogs and cats in Brazil are discussed.

## Review

### Ectoparasites of dogs and cats in Brazil

Ectoparasites may cause irritation, blood depletion, pruritus, and skin lesions, potentially leading to the occurrence of secondary bacterial infections. Some ectoparasites such as fleas, lice and ticks may also transmit pathogens to dogs and cats, including bacteria, protozoa, and helminths reviewed in Ref. [9]. Furthermore, ticks can also cause toxicosis, as it has recently been reported in a dog bitten by the *mouro* tick *Ornithodoros brasiliensis* in Brazil [10].

In Brazil, dogs and cats may serve as hosts for a large number of ectoparasites, including fleas, lice, mites, and ticks (Tables 1 and 2). Indeed, dogs may be frequently infested by species of fleas (e.g., *Ctenocephalides canis*, *Ctenocephalides felis felis*, and *Tunga penetrans*), lice (e.g., *Heterodoxus spiniger* and *Trichodectes canis*), mites (e.g., *Demodex canis*, *Otodectes cynotis*, and *Sarcoptes scabiei*) and ticks (e.g., *Amblyomma aureolatum*, *Amblyomma ovale*, and *Rhipicephalus sanguineus* sensu lato) [11-41]. While some flea species may be common in rural settings (e.g., *T. penetrans*), *C. felis felis* is indeed the most common flea infesting dogs in Brazil. Other less common ectoparasites (e.g., the flea *Rhopalopsyllus lutzi lutzi*) have also been reported in dogs in Brazil [21,28]. In a similar manner, the findings of some wildlife-associated ticks (e.g., *Amblyomma longirostre*, *Amblyomma naponsense*, *Amblyomma nodosum*, *Amblyomma pacae*, *Amblyomma scalpturatum*, and *Haemaphysalis juxtakochi*) on dogs in Brazil are incidental findings and of unknown clinical significance.

**Table 1:** Ectoparasites of dogs in Brazil

**Phylum**	**Class**	**Order**	**Family**	**Species**^**a**^
Arthropoda	Arachnida	Ixodida	Argasidae	*Ornithodoros brasiliensis*
				*Ornithodoros rostratus*
			Ixodidae	*Amblyomma aureolatum*
				*Amblyomma cajennense*
				*Amblyomma longirostre*
				*Amblyomma naponsense*
				*Amblyomma nodosum*
				*Amblyomma oblongoguttatum*
				*Amblyomma ovale*
				*Amblyomma pacae*
				*Amblyomma parvum*
				*Amblyomma scalpturatum*
				*Amblyomma tigrinum*
				*Haemaphysalis juxtakochi*
				*Rhipicephalus microplus*
				*Rhipicephalus sanguineus* s.l.
		Sarcoptiformes	Psoroptidae	*Otodectes cynotis*
			Sarcoptidae	*Notoedres cati*
				*Sarcoptes scabiei*
		Trombidiformes	Demodicidae	*Demodex canis*
	Insecta	Phthiraptera	Boopidae	*Heterodoxus spiniger*
			Linognathidae	*Linognathus setosus*
			Trichodectidae	*Trichodectes canis*
		Siphonaptera	Ceratophyllidae	*Nosopsyllus fasciatus*
			Tungidae	*Tunga penetrans*
			Pulicidae	*Ctenocephalides canis*
				*Ctenocephalides felis felis*
				*Pulex irritans*
				*Xenopsylla brasiliensis*
				*Xenopsylla cheopis*
			Rhopalopsyllidae	*Rhopalopsyllus lutzi lutzi*

^a^Checklist of ectoparasites (flying insects excluded) infesting dogs in Brazil [11-41].

**Table 2:** Ectoparasites of cats in Brazil

**Phylum**	**Class**	**Order**	**Family**	**Species**^**a**^
Arthropoda	Arachnida	Ixodida	Ixodidae	*Amblyomma aureolatum*
				*Amblyomma ovale*
				*Amblyomma triste*
				*Rhipicephalus sanguineus* s.l.
		Sarcoptiformes	Listrophoridae	*Lynxacarus radovskyi*
			Psoroptidae	*Otodectes cynotis*
			Sarcoptidae	*Notoedres cati*
				*Sarcoptes scabiei*
		Trombidiformes	Demodicidae	*Demodex cati*
	Insecta	Phthiraptera	Trichodectidae	*Felicola subrostratus*
		Siphonaptera	Pulicidae	*Ctenocephalides canis*
				*Ctenocephalides felis felis*
				*Pulex irritans*
			Tungidae	*Tunga penetrans*

^a^Checklist of ectoparasites (flying insects excluded) infesting cats in Brazil [19,22,42-49].

Cats in Brazil are frequently found infested by fleas (e.g., *C. canis*, *C. felis felis*, *P. irritans*, and *T. penetrans*), lice (*Felicola subrostratus*), and mites (e.g., *D. cati*, *Lynxacarus radovskyi*, *Notoedres cati*, and *O. cynotis*) [19,22,42-49]. Less frequently, they have been found infested by ticks such as *R. sanguineus* s.l. and *Amblyomma triste*[49,50]. Some of these ectoparasites are well known in certain regions (e.g., *L. radovskyi* in north-eastern Brazil), but completely unknown or considered rare in others. Even if cats have more knowhow than dogs when the issue is “ectoparasite removal” by grooming, they may be highly exposed to ectoparasites, particularly in high-density situations such as in catteries [45].

**Figure 3:**
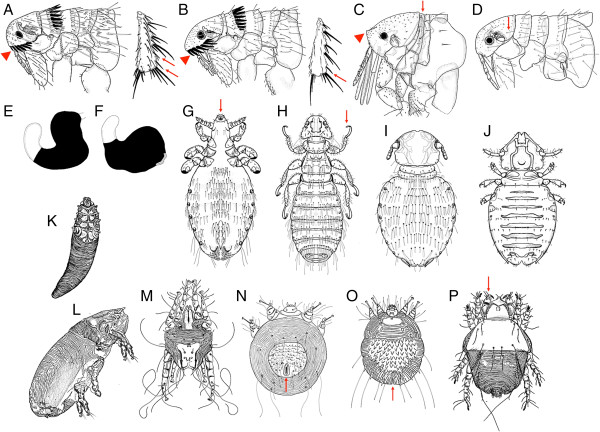
**Ectoparasites of dogs and cats.** Line drawings for the identification of common dog and cat ectoparasites (fleas: **A-F**; lice: **G-J** and mites: **K-P**) found in Brazil. For details see Table 3).

Even if most fleas, lice, mites, and ticks infesting dogs and cats may be easily identified, the delineation of some species may be troublesome for non-taxonomists [51,52]. While some keys for fleas, lice, and ticks have been published in books [13,14,53] and journals [54-56], there are no comprehensive pictorial keys for the identification of dog and cat ectoparasites in Brazil (see Table 3 and Figure 3).

**Table 3:** Key for ectoparasites identification

1a	Three pairs of legs	**2**
1b	Four pairs of legs	**13**
2a	Body laterally flattened (SIPHONAPTERA)	**3**
2b	Body dorsoventrally flattened (PHTHIRAPTERA)	**10**
3a	Pronotal and/or genal ctenidia present	**4**
3b	Pronotal and genal ctenidia absent	**6**
4a	Genal ctenidium absent and pronotal ctenidium present	***Nosopsyllus fasciatus***
4b	Genal and pronotal ctenidia present	**5**
5a	Genal ctenidium with the first seta much shorter than the others (Figure 3A; arrowhead); posterior tibia with two simple setae between the penultimate and the last double setae (Figure 3A; arrows)	***Ctenocephalides canis***
5b	Genal ctenidium with the first seta approximately the same length of the remaining (Figure 3B; arrowhead); posterior tibia with one simple seta between the penultimate and the last double setae (Figure 3B; arrow)	***Ctenocephalides felis felis***
6a	Thoracic segments narrow (Figure 3C; arrow), ocular and occipital setae absent; front of the head angular (upturned) (Figure 3C; arrowhead)	***Tunga penetrans***
6b	Not as above	**7**
7a	Abdominal segments with two rows of setae in the dorsal border	***Rhopalopsyllus lutzi lutzi***
7b	Abdominal segments with one row of setae in the dorsal border	**8**
8a	Occipital region with one seta (Figure 3D; arrow)	***Pulex irritans***
8b	Occipital region with two rows of setae arranged in a “V”	**9**
9a	Male antepygidial seta inserted on a discrete tubercle; female with body of spermatheca (in black) not larger than the base of tail (Figure 3E)	***Xenopsylla cheopis***
9b	Male antepygidial seta inserted on a well-developed tubercle; female with body of spermatheca (in black) larger than the base of tail (Figure 3F)	***Xenopsylla brasiliensis***
10a	Head longer than large (Figure 3G; arrow); sucking mouthparts	***Linognathus setosus***
10b	Head as large as long; chewing mouthparts	**11**
11a	Tarsus with two claws (Figure 3H; arrow)	***Heterodoxus spiniger***
11b	Tarsus with one claw	**12**
12a	Head rounded anteriorly (Figure 3I)	***Trichodectes canis***
12b	Head triangular in shape (Figure 3J)	***Felicola subrostratus***
13a	Body length equal or less than 0.5 mm; Haller’s organ absent	**14**
13b	Body length larger than 0.5 mm; Haller’s organ present on first tarsi (IXODIDA)	**19**
14a	Body carrot-shaped or with a bell pepper shape (TROMBIDIFORMES)	**15**
14b	Body not as above, usually rounded in shape (SARCOPTIFORMES)	**16**
15a	Body long and slender (carrot-shaped) (Figure 3K)	***Demodex *** **spp.**^**a**^
15b	Body with a bell pepper shape; well-developed mouthparts; palpi that end in prominent hooks (Figure 3L; arrow)	***Cheyletiella *** **spp.**^**b**^
16a	Body rounded	**17**
16b	Body cylindrical and elongate, heavily striated (Figure 3M)	***Lynxacarus radovskyi***
17a	Legs long, with short, unjointed pedicels (Figure 3N)	***Otodectes cynotis***
17b	Legs short, with long, unjointed pedicels	**18**
18a	Anus dorsal (Figure 3O; arrow)	***Notoedres cati***
18b	Anus terminal (Figure 3P; arrow); body surface covered by several triangular spines arranged in rows	***Sarcoptes scabiei***
19a	Inornate; *basis capituli* hexagonal; coxa I bifid	***Rhipicephalus sanguineus *** **s.l.**^**c**^
19b	Usually ornate; *basis capituli* variable in form	***Amblyomma *** **spp.**^**d**^

^a^*Demodex canis* in dogs and *Demodex cati* in cats.

^b^No *bona fide* data on *Cheyletiella* spp. in dogs or cats in Brazil.

^c^At least two distinct biologically and genetically distinct tick populations under the name “*R. sanguineus*” have been found on dogs in South America [52,161,162].

^d^Several species reported in dogs and cats. *Amblyomma aureolatum*, *Amblyomma cajennense* and *Amblyomma ovale* are among the most common *Amblyomma* species found on rural dogs in Brazil [55].

Flying insects may also represent a significant clinical problem to dogs as cats in Brazil [9,57-66]. For instance, mosquitoes, phlebotomine sand flies and triatomine bugs are vectors of *Dirofilaria immitis*, *Leishmania infantum*, and *Trypanosoma cruzi*, respectively, to both dogs and cats [9]. Myiasis is also quite a common problem in dogs and cats in Brazil, being most frequently associated with infestation by larvae of *Cochliomyia hominivorax* and *Dermatobia hominis *[58-65], and less commonly by other species of flies (e.g., *Lucilia eximia*) [57,65]. Furthermore, stable flies (*Stomoxys calcitrans*) can produce skin lesions on the ears of these animals, which may also lead to the occurrence of secondary bacterial infections [9,66].

### Endoparasites of dogs and cats in Brazil

In addition to ectoparasites, dogs in Brazil may be affected by numerous endoparasites (Table 4), including protozoa, cestodes (tapeworms), trematodes (flukes), and nematodes (roundworms) [32,67-130]. For instance, endoparasites such as *Dipylidium caninum*, *Echinococcus granulosus*, *A. braziliense*, *Ancylostoma caninum*, *Strongyloides stercoralis*, *Toxocara canis*, *Trichuris vulpis*, and *Dirofilaria immitis* are commonly found in dogs from different Brazilian regions [67,69]. Other endoparasites such as *Angiostrongylus vasorum* have only sporadically been reported in Brazil [74]. Similarly, cats have frequently been found affected by several endoparasites (Table 5) in Brazil, including *T. gondii*, *D. caninum*, *Taenia taeniformis*, *Spirometra mansonoides*, *Aelurostrongylus abstrusus*, *A. braziliense*, *A. caninum*, *A. tubaeforme*, *Toxascaris leonina*, *Toxocara cati*, *Pearsonema feliscati*, *Trichuris campanula*, *Trichuris serrata*, *Physaloptera praeputialis*, and *Platynosomum fastosum *[47,71,87,90,92,97,131-137]. Less common endoparasites have also been reported in cats, such as the acanthocephalan *Sphaerirostris erraticus *[137].

**Table 4:** Endoparasites of dogs in Brazil

**Phylum**	**Class**	**Order**	**Family**	**Species**^**a**^
Apicomplexa	Aconoidasida	Piroplasmida	Babesiidae	*Babesia gibsoni*
				*Babesia vogeli*
			Theileriidae^b^	*Rangelia vitalii*
	Conoidasida	Eucoccidiorida	Hepatozoidae	*Hepatozoon canis*
			Cryptosporidiidae	*Cryptosporidium canis*
			Eimeridae	*Isospora canis* (syn. *Cystoisospora canis*)
				*Isospora ohioensis* (syn. *Cystoisospora ohioensis*)
			Sarcocystidae	*Hammondia heydorni*
				*Neospora caninum*
				*Sarcocystis capracanis*
				*Sarcocystis cruzi* (syn. *Sarcocystis bovicanis*)
				*Sarcocystis levinei*
				*Sarcocystis miescheriana* (syn. *Sarcocystis suicanis*)
				*Sarcocystis tenella* (syn. *Sarcocystis ovicanis*)
Metamonada	Parabasalia	Trichomonadida	Trichomonadidae	*Pentatrichomonas hominis* (syn. *Trichomonas hominis*)
Sarcomastigophora	Zoomastigophora	Diplomonadida	Hexamitidae	*Giardia duodenalis* (syn. *Giardia intestinalis*)
		Kinetoplastida	Trypanosomatidae	*Leishmania infantum* (syn. *Leishmania chagasi*)
				*Leishmania amazonensis*
				*Leishmania braziliensis*
				*Trypanosoma caninum*
				*Trypanosoma evansi*
				*Trypanosoma cruzi*
Nematoda	Secernentea	Strongylida	Ancylostomatidae	*Ancylostoma braziliense*
				*Ancylostoma caninum*
			Angiostrongylidae	*Angiostrongylus vasorum*
		Rhabditida	Strongyloididae	*Strongyloides stercoralis*
		Ascaridida	Ascarididae	*Lagochilascaris minor*
				*Toxascaris leonina*
				*Toxocara canis*
			Dioctophymatidae	*Dioctophyme renale*
		Trichurida	Trichinellidae	*Calodium hepaticum* (syn. *Capillaria hepatica*)
			Trichuridae	*Trichuris vulpis*
		Spirurida	Gongylonematidae	*Gongylonema pulchrum*
			Onchocercidae	*Acanthocheilonema reconditum*
				*Cercopithifilaria bainae*
				*Cercopithifilaria grassii* (syn. *Dipetalonema grassii*)
				*Dirofilaria immitis*
			Physalopteridae	*Physaloptera praeputialis*
			Spirocercidae	*Spirocerca lupi*
Platyhelminthes	Cestoda	Cyclophyllidea	Dipylidiidae	*Dipylidium caninum*
			Taeniidae	*Echinococcus granulosus*
				*Taenia hydatigena*
				*Taenia multiceps* (syn. *Multiceps multiceps*)
	Trematoda	Opisthorchiida	Heterophyidae	*Ascocotyle longa* (syn. *Phagicola arnaldoi*)
Acanthocephala	Archiacanthocephala	Oligacanthorhynchida	Oligacanthorhynchidae	*Oncicola canis*

^a^Checklist of endoparasites (protozoa and helminths) affecting dogs in Brazil [32,67-130,344-353] Doubtful records (e.g., *Phagicola minuta*, *Aelurostrongylus abstrusus*, *Dirofilaria repens*, *Necator americanus*, *Trichuris serrata*, *Uncinaria stenocephala*, *Isospora rivolta*, and *Isospora bigemina*) [68,69] are not included.

^b^The genus *Rangelia* has long been regarded a synonym of *Babesia* (family Babesiidae), but recent genetic and biological data supports its validity and its inclusion in the family Theileriidae. The creation of a monospecific family (Rangeliidae) does not seem desirable.

**Table 5:** Endoparasites of cats in Brazil

**Phylum**	**Class**	**Order**	**Family**	**Species**^**a**^
Sarcomastigophora	Zoomastigophora	Diplomonadida	Hexamitidae	*Giardia duodenalis*
		Kinetoplastida	Trypanosomatidae	*Leishmania infantum*
				*Leishmania amazonensis*
				*Leishmania braziliensis*
				*Trypanosoma cruzi*
Apicomplexa	Aconoidasida	Piroplasmida	Babesiidae	*Babesia vogeli*
			Theileriidae	*Cytauxzoon felis*
	Conoidasida	Eucoccidiorida	Hepatozoidae	*Hepatozoon canis*
				*Hepatozoon felis*
			Cryptosporidiidae	*Cryptosporidium felis*
			Eimeriidae	*Isospora felis* (syn. *Cystoisospora felis*)
				*Isospora felis* (syn. *Cystoisospora rivolta*)
			Sarcocystidae	*Hammondia hammondi*
				*Sarcocystis hirsuta* (syn. *Sarcocystis bovifelis*)
				*Toxoplasma gondii*
Nematoda	Secernentea	Strongylida	Ancylostomatidae	*Ancylostoma braziliense*
				*Ancylostoma caninum*
				*Ancylostoma tubaeforme*
			Angiostrongylidae	*Aelurostrongylus abstrusus*
				*Gurltia paralysans*
			Syngamidae	*Mammomonogamus dispar*
		Rhabditida	Strongyloididae	*Strongyloides stercoralis*
		Ascaridida	Ascarididae	*Lagochilascaris minor*
				*Toxascaris leonina*
				*Toxocara cati* (syn. *Toxocara mistax*)
			Dioctophymatidae	*Dioctophyme renale*
		Trichurida	Capillariidae	*Calodium hepaticum* (syn. *Capillaria hepatica*)
				*Pearsonema feliscati*
			Trichuridae	*Trichuris campanula*
				*Trichuris serrata*
		Spirurida	Onchocercidae	*Dirofilaria immitis*
			Physalopteridae	*Physaloptera praeputialis*
Platyhelminthes	Cestoda	Cyclophyllidea	Dipylidiidae	*Dipylidium caninum*
			Taeniidae	*Taenia taeniformis*
		Pseudophyllidea	Diphyllobothriidae	*Spirometra mansonoides*
	Trematoda	Opisthorchiida	Heterophyidae	*Ascocotyle angrense*
				*Ascocotyle longa* (syn. *Phagicola arnaldoi*)
		Plagiorchiida	Dicrocoeliidae	*Platynosomum fastosum*
		Strigeatida	Diplostomatidae	*Alaria alata*
Acanthocephala	Archiacanthocephala	Moniliformida	Moniliformidae	*Moniliformis moniliformis*
	Palaecanthocephala	Polymorphida	Centrorhynchidae	*Sphaerirostris erraticus*

^a^Checklist of endoparasites (protozoa and helminths) affecting cats in Brazil [42,44,47,71,87,90,92,97],[129,131-148,346,349,352-356]. Doubtful records (e.g., *Ascaridia galli* and *Heterakis gallinarum*) [68,69] are not included.

Dogs have been found infected by vector-borne protozoa, including *Babesia gibsoni*, *Babesia vogeli*, *Rangelia vitalii*, *Hepatozoon canis*, *Leishmania amazonensis*, *Leishmania braziliensis*, *Leishmania infantum*, *Trypanosoma cruzi*, and *Trypanosoma evansi *[73,78-82,85,87-90,96,102-104,109-113,117-121],[124,128-130]. Other protozoa of unknown pathogenicity (e.g., *Trypanosoma caninum*) have also been described in Brazilian dogs [108]. In addition, other vector-borne organisms have also been frequently reported in dogs, including *Anaplasma platys*, *Ehrlichia canis* and, less frequently, *Rickettsia rickettsii *[9].

Cats have also been found infected by vector-borne protozoa such as *B. vogeli*, *Cytauxzoon felis*, *H. canis*, *Hepatozoon felis*, *L. amazonensis*, *L. braziliensis*, and *L. infantum *[42,44,129,136,138-148]. Indeed, the diversity of endoparasites of cats and dogs in Brazil is impressive, but certainly underestimated, mainly because most studies carried out in this country have been based on the detection and identification of eggs and oocysts in faecal samples using ordinary coprological techniques. This emphasizes the need for additional diagnostic methods (e.g., coproantigen tests, molecular tests or *post-mortem* examination) if we are to understand the actual diversity and prevalence of endoparasites of dogs and cats in Brazil.

### Geographical distribution and prevalence

Dog and cat parasites are widespread in Brazil and in most cases occur virtually in all geographical regions (north, northeast, south, southeast, and centre-west) of the country. However, for several common external parasites (e.g., *D. canis*, *N. cati *e *O. cynotis*) and internal (e.g., *D. caninum*, *A. brasiliense *e *T. canis*) there is relatively limited published information, particularly in international journals. Therefore, it is presently difficult to generate informative, *bona fide* maps for most dog and cat parasites occurring in Brazil, even if most of these parasites are certainly present in all regions of the country. For instance, in a study carried out in south-eastern Brazil, 155 dogs were necropsied and *D. caninum* was diagnose in 57 (36.8%) of them, *A. caninum* in 30 (19.4%), and *T. canis* in 24 (15.5%) [36]. Similarly, a study conducted in north-eastern Brazil, revealed that *A. caninum* (prevalence, 95.7%) was the most prevalent endoparasite in 46 stray dogs necropsied, followed by *D. caninum* (45.7%), *T. canis* (8.7%), and *T. vulpis* (4.3%) [32]. These studies indicate high prevalence rates of infestation by endoparasites in stray dogs from different Brazilian regions representing a permanent source of endoparasites to pet animals and a major zoonotic hazard for humans, through the contamination of public parks and beaches (see “Dogs, cats, parasites and humans: public health aspects”).

Stray cats are also frequently exposed to endoparasites. For instance, a study conducted in south-eastern Brazil reported infestations by *A. braziliense* (67.3%), *A. caninum* (21.1%), and *A. tubaeforme* (9.6%) in 52 cats necropsied [116]. Similarly, in a recent study conducted in centre-western Brazil, 146 cats were necropsied and 98 of them were infested by roundworms [*A. braziliense* (50.68%); *A. tubaeforme* (10.27%); *T. cati* (4.11%); *P. praeputialis* (2.05%); *P. feliscati* (3.42%); and *A. abstrusus* (1.37%)], cestodes [*S. mansonoides* (4.11%); *D. caninum* (3.42%); and *T. taeniformis* (0.68%)], trematodes [*P. fastosum* (26.03%)], and acanthocephalans [*S. erraticus* (3.42%)] [137]. Again, these studies reveal a considerable diversity of endoparasites in cats from different Brazilian regions, with relatively high prevalence rates.

The geographical distribution of vector-borne protozoa and heminths of dogs has been reviewed elsewhere [9]. For instance, *L. infantum* is widespread in all Brazilian regions [81,113,149,150], but less frequent in southern parts of the country [151,152]. However, *L. longipalpis*, the principal vector for *L. infantum*, is present in northern Argentina [153], Paraguay [154], and has recently been detected in Uruguay [155]. Furthermore, *L. longipalpis* is abundant in Mato Grosso do Sul State [156], which makes border with Paraná State in southern Brazil. Last but not least, the presence of *L. longipalpis* in Rio Grande do Sul (the southernmost Brazilian state) has been proven [157]. These findings may suggest that *L. longipalpis* may have already colonized other areas of southern Brazil and that canine leishmaniosis may become a problem in that region in the near future.

The little known protozoan *R. vitalii* occurs mainly in southern and south-eastern Brazil [119,124,158,159], where ticks such as *A. aureolatum*, the putative vector, are commonly found in dogs frequenting the Atlantic rainforest environment [160]. Interestingly, *B. gibsoni* has been reported exclusively in southern Brazil [87,104], which also suggests that *R. sanguineus* s.l. is not the vector of this protozoan in Brazil, considering that this tick is prevalent in virtually all regions of the country [52,161,162]. In fact, pathogens that are transmitted by *R. sanguineus* s.l. (e.g., *B. vogeli* and *Ehrlichia canis*) are usually widespread in Brazil [9], not concentrated in a specific region as it occurs with *B. gibsoni*. In any case, the possibility that *R. sanguineus* s.l. ticks are acting as vectors of *B. gibsoni* in Brazil cannot be ruled out.

*Ctenocephalides* spp. fleas are also widespread in Brazil [18,35,36,38,129], but *C. felis felis* surpasses *C. canis* in distribution, the latter being reputed to be more common in regions with more temperate climates, as in the south-east and south of the country [51]. Indeed, *C. felis felis* has been reported in 17 states (Alagoas, Amazonas, Bahia, Ceará, Espírito Santo, Goiás, Mato Grosso, Minas Gerais, Paraíba, Paraná, Pernambuco, Rio de Janeiro, Rio Grande do Norte, Rio Grande do Sul, Roraima, Santa Catarina, and São Paulo), while *C. canis* has been reported in only nine (Amazonas, Bahia, Maranhão, Minas Gerais, Paraná, Pernambuco, Rio Grande do Sul, Santa Catarina, and São Paulo) [51]. As a consequence of the widespread distribution of *Ctenocephalides* spp. fleas on dogs and cats, flea-borne parasites like *D. caninum* and *A. reconditum *[163-167] are widespread in Brazil as reviewed in Ref. [9].

Even if fleas, lice and ticks may be found infesting dogs in all Brazilian regions, the distribution and prevalence at the local level may vary widely, also according to climate conditions and the degree of urbanization of each area. For instance, in a study conducted in north-eastern Brazil, the frequency of infestation by *R. sanguineus* s.l. tended to be higher in urban than in rural areas, whereas infestations by *Amblyomma* ticks and *C. felis felis* was more common on rural dogs [39]. In the same study, mixed infestations were significantly more frequent on rural than on urban dogs. Nonetheless, even if *R. sanguineus* s.l. ticks are more prevalent in urban settings [18,28,32], they may infest a high proportion of rural dogs in some areas [30,33,36].

Serological surveys have also reported moderate to high levels of exposure to parasites such as *Neospora caninum* and *T. gondii* in dogs and cats in Brazil [151,168-173]. For instance, in a recent study carried out in south-eastern Brazil, 386 cats were assayed by indirect immunofluorescence antibody tests (IFAT) for the presence of antibodies against *T. gondii* and 63 (16.3%) of them were positive [172]. Prevalence rates of anti-*N. caninum* antibodies in dogs ranging from 26% to 34.5% were reported in north-eastern Brazil, with 57.6% of the seropositive dogs also being positive for anti-*T. gondii* antibodies [169]. In another study conducted in south-eastern Brazil, 703 dogs from urban and rural areas were tested for *N. caninum* and 11.4% of them were positive. The likelihood of being positive to *N. caninum* was associated with dogs aged >4 years, guard dogs, hunting dogs, and with dogs from dairy farms with a history of bovine abortion [173]. In particular, among rural dogs, an association with seroprevalence was recorded for milk farms, dogs not fed with commercial food, hunting, and guard dogs. Altogether, these studies reveal that dogs and cats from different regions both rural and urban areas are at risk of infection by parasites such as *N. caninum* and *T. gondii*. This is also true for *Leishmania* spp. in dogs, for which seroprevalence rates higher than 60% have been reported in some highly endemic areas [168].

### Seasonal dynamics in the tropics

Information on the ecology of parasites of dogs and cats in Brazil is fragmentary, meagre, and, for several parasites, completely lacking. Considering the variety of biomes (Atlantic Rainforest, Amazon, Pantanal, Cerrado, Caatinga, and Pampa) and climates (from tropical north to temperate zones below the Tropic of Capricorn) registered in Brazil, the seasonal dynamics of dog and cat parasite populations would be expectedly variable from region to region. Nonetheless, the climate conditions in Brazil are so suitable that a range of arthropod vectors are found the year-round. For instance, studies carried out in an area of *D. immitis* endemicity in Rio de Janeiro indicated that mosquito vectors (e.g., *Ochlerotatus scapularis* and *Ochlerotatus taeniorhynchus*) are more abundant during summer months, but may also be captured in high number in winter months [174]. These findings suggest that dogs and cats may be at risk of *D. immitis* infestation during the whole year, especially because the average annual temperature in most of the Brazilian territory is between 21 and 30°C, which indeed facilitates the development of infective larvae (L3) in the mosquito vector.

**Figure 4:**
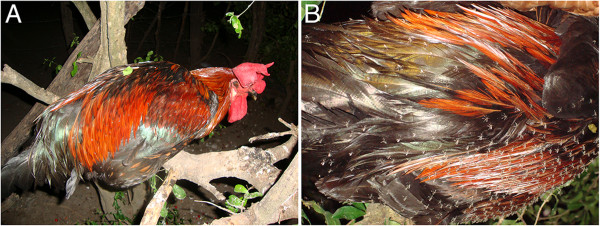
**Vector density.** Hundreds of phlebotomine sand flies (*Lutzomyia longipalpis*) on a chicken **(A)** that was resting on a tree trunk in Passira, north-eastern Brazil, where visceral leishmaniosis is endemic. **(B)** shows a close-up of the phlebotomine sand flies

The presence of ectoparasites during the whole year in Brazil is apparently the rule, not the exception. Phlebotomine sand fly vectors of *Leishmania* parasites such as *L. longipalpis* (Figure 4) and *L. whitmani* may be found in high numbers throughout the year in different Brazilian regions [175-180], even if some species may display population peaks during the months of highest rainfall [181]. The same applies for fleas and ticks. A recent study developed in south-eastern Brazil revealed high prevalence rates of flea (*C. felis felis*) and tick (*A. cajennense* and *R. sanguineus s.l.*) infestations during the dry and rainy seasons [35]. Another study carried out in the same region indicated that *R. sanguineus s.l.* may infest dogs during any period of the year and completes three generations per year [182]. Similarly, an investigation conducted in centre-western Brazil suggested that this tick may complete up to four generations per year [183]. Again, these findings suggest that different Brazilian regions possess climate types and environmental conditions that enable the occurrence of ticks during all months of the year. For example, even if a study has identified variations in *B. vogeli* infection in dogs apparently associated with peaks in the tick vector population [184], evidence indicates that this protozoan may infect dogs at any period of the year in some regions, regardless of the season [120]. On the other hand, myiasis by *C. hominivorax* in dogs and cats presents a clearly defined seasonal pattern in Brazil, peaking during the hottest months of the year [59-61,65].

Data concerning the seasonal dynamics of endoparasites in dogs and cats in Brazil is meagre. The incidence of hookworm-related cutaneous *larva migrans* is higher during the rainy season [185]. Interestingly, a study reported a marked peak in *Ancylostoma* eggs shedding in dog faeces during summer and autumn, suggesting that winter conditions in São Paulo (south-eastern Brazil) have a negative effect on hookworm transmission [74]. Another study concluded that the southern region of Brazil has favourable edaphic and climatic conditions for soil contaminants (e.g., *Toxocara* eggs) to persist year-round, even if the species diversity of parasites was lower in winter and the number of parasites lower in summer [186]. This is in agreement with studies carried out in other parts of South America. For instance, a study conducted in Mar del Plata, Argentina, reported a high prevalence of intestinal parasites of dogs throughout the year, with some seasonal variations according to the genus or species of parasite [187]. Hookworms (*Ancylostoma* spp.) were more prevalent in the summer-autumn period, ascarids (*T. canis*) in winter, whereas whipworms (*T. vulpis*) peaked in winter, spring and summer. Once again, these data demonstrate a high risk of parasite infestation in all seasons in this part of the Americas.

### Diagnostic and management approaches

#### ***Diagnosis of parasitic diseases***

The diagnosis of parasitic diseases affecting dogs and cats in Brazil is still predominately made by traditional methods. For instance, gastrointestinal parasites are usually detected by ordinary coprological techniques, such as Willis (flotation in saturated sodium chloride solution), Faust (zinc sulphate centrifugal flotation) and Hoffman-Pons-Janer (spontaneous faecal sedimentation in water) techniques [188,189]. These methods may present low sensitivity in some instances and result in the underestimation of the real prevalence of some parasites, such as *D. caninum *[74,77,100,188,190], when compared with necropsy data e.g., [32]. A commercial assay for faecal concentration (namely, TF-test^®^) designed for detecting human intestinal parasites [191] has also been used for detecting helminth eggs, protozoan cysts and oocysts in canine faeces [190]. A comparative study revealed that the centrifugal flotation technique was more sensitive than centrifugal sedimentation and TF-test^®^ in detecting *Ancylostoma* spp., *T. canis*, *T. vulpis* and *Giardia* in dog faeces [190]. Another study reported that the Willis technique was more efficient in detecting eggs of *A. caninum* and *T. canis* in dog faeces [188]. As a corollary, a more recent study showed that the Willis and the centrifugal flotation techniques performed better than the Hoffman-Pons-Janer technique for detecting *Ancylostoma* spp. in dog faeces [189]. Other methods have also been proposed, but apparently with no significant difference in terms of sensitivity, as compared with traditional methods [98]. The use of an enzyme linked immunosorbent assay (ELISA) for the detection *E. granulosus* coproantigen revealed high positivity rates (from 27.69 to 47.37%) in rural dogs from southern Brazil [192]. In the same way, PCR-based techniques have been used to detect endoparasites (heminths and protozoa) of dogs in Brazil e.g., [83,126,193,194] but currently these methods are mostly restricted to research.

Blood-dwelling protozoan parasites (e.g., *B. vogeli* and *H. canis*) are usually diagnosed by examination of stained-blood smears under a light microscope, which may lack in sensitivity, particularly if blood samples are collected outside the acute phase of the infection [195]. Serological tests are widely used to assess exposure to pathogens, such as *B. vogeli*, *Leishmania* spp., *N. caninum*, and *T. gondii *[168,169,184,196-198]. The use of molecular tools for diagnosing protozoan parasites (e.g., *B. vogeli*, *H. canis*, and *L. infantum*) is becoming more and more popular, but it is still mostly restricted to research [121,199-204]. Indeed, current PCR protocols have shown a good level of concordance with parasitological methods [150]. Unfortunately, the costs of molecular tools are still prohibitive for most pet owners living in endemic areas and this severely impairs the diagnosis of diseases such as visceral and cutaneous leishmaniosis in dogs. Indeed, current serological tools cannot distinguish between *L. braziliensis* and *L. infantum *[113,205]. This may have direct implications for veterinary practitioners in Brazil because seropositive dogs are usually eliminated as part of the control programme against human visceral leishmaniosis, whilst it has been demonstrated that many of these dogs are infected by *L. braziliensis* rather than *L. infantum *[113,205,206]. Indeed, the high diversity of parasites in Brazil may be a confounding factor for the diagnosis of some diseases in dogs and cats in this country. For instance, different *Leishmania* species have been reported in these animals in Brazil and the clinical picture is not always predictable. A recent report described a case of canine leishmaniosis by *L. infantum* in which the dog presented a skin ulcer that is typically found in dogs and humans infected by *L. braziliensis *[207]. Again, dogs infected by *L. amazonensis* generally display clinical signs commonly observed in dogs with visceral leishmaniosis [96,117]. On the other hand, cats infected by *L. amazonensis* typically display a cutaneous disease that resembles sporotrichosis [141,208]. It means that the same parasite may eventually induce different clinical diseases in dogs and cats, which emphasizes the need for a proper laboratory diagnosis to confirm the parasite species involved. This is also true for eggs of ascarids (*Toxocara* spp.), and taenids (*Taenia* spp. and *Echinococcus* spp.) found in dog faeces, which cannot be normally differentiated by light microscopy [209,210]. Hookworm eggs are not easily distinguishable by morphology, but morphometric data suggests that they can be differentiated [211]. Nonetheless, the use of improved, specific diagnostic techniques is fundamental towards a better management of parasitic diseases of dogs and cats in Brazil.

#### ***Controlling dog and cat parasites***

Veterinary practitioners working in Brazil are provided with a relatively vast repertory of tools to control parasites affecting dogs and cats. Nonetheless, the control of common parasites such as fleas and ticks may sometimes appear quite difficult, mainly when the available tools are not properly used or when veterinary practitioners do not possess basic knowledge on the bio-ecology of some of these parasites. In Brazil, there are no official guidelines for the control of ecto- and endoparasites of dogs and cats, such as those provided by European Scientific Counsel Companion Animal Parasites (ESCCAP: http://www.esccap.org/) in Europe and the Companion Animal Parasite Council (CAPC: http://www.capcvet.org/) in the United States. These guidelines are fundamental to ensure the delivery of the latest scientific information on parasite control to veterinary practitioners.

Because lice and mites live in permanent association with their hosts, they are relatively easily controlled and, once eliminated, re-infestation only occurs following contact with other infested animals [212,213]. As such, the control of louse infestations in dogs and cats is mostly based on the application of insecticides on infested animals, which may be available as collars, spot-on solutions, and sprays [214-216]. Mites are also permanent ectoparasites, with some of them living in the fur (*L. radovskyi*), others in the outer ear canal (*O. cynotis*), hair follicles and sebaceous glands (*D. canis* and *D. cati*), or even in galleries burrowed under the epidermis (*N. cati *e and *S. scabiei*). The treatment may be systemic (e.g., oral selamectin for *S. scabiei*) or topic (e.g., amitraz dip for *D. canis*). For instance, the treatment of infestations by the cat fur mite (*L. radovskyi*) in Brazil has been successfully performed with weekly baths using a soap containing tetramethylthiuram monosulfide [217]. Similarly, a single subcutaneous injection of doramectin 1% (0.3 mg/kg) has been effective in treating dogs with sarcoptic mange and endoparasites as well [218]. However, the treatment of demodicosis in dogs may vary depending on case severity, animal age, and the presence of underlying conditions [219]. Indeed, most young dogs with juvenile-onset localized demodicosis will resolve spontaneously without treatment, whereas adult dogs with generalized demodicosis usually require aggressive therapy, for example, with amitraz dip every two weeks for several months [219]. Furthermore, an underlying disease or condition (e.g., cancer, hyperadrenocorticism, use of immunosuppressive drugs) should always be investigated and treated accordingly, especially in cases of demodicosis in adult dogs. It is clear that the treatment of mange in dogs and cats depends on several factors linked to the mite species involved and to the characteristics of each individual patient. Veterinary practitioners should use the products specifically labelled for mange treatment in dogs and cats in Brazil and strictly follow the manufacturer’s recommendations.

In contrast to mites and lice, ticks and fleas are non-permanent parasites [212] and therefore specimens found on dogs and cats may represent only a small proportion of the whole flea and tick population present in the environment where infested animals live. A vast repertory of products are available in the veterinary market in Brazil for controlling fleas and ticks on dogs and cats. Insecticides and acaricides with potent anti-feeding and fast killing effects, repellents, insect growth regulators, and juvenile hormone analogues are available as spot-on pipettes, collars, shampoos, and sprays [212,220]. However, before deciding the best control strategy to be adopted, the veterinary practitioner should also investigate the presence and the abundance of fleas/ticks in the environment where the animal lives. Ticks tend to drop off during the night when the dog is sleeping [221]. Moreover, flea eggs are not sticky, falling from the animal’s fur in the environment along with adult flea faeces. Newly hatched larvae usually avoid sunlight and actively move deep into carpet fibres or under organic debris [222]. Vacuuming places frequented by pets (e.g., carpets, furniture cushions, rugs, or other substrata), with a vacuum cleaner as well as washing of pet bedding or bed linen frequented by pets will help in the removal of flea eggs and larvae [222]. Furthermore, the treatment of the indoor and outdoor environment with insecticides and acaricides may be required in some situations, such as in kennels and catteries with high levels of environment flea and tick infestation. Care should be taken when applying chemical products in the environment and it is advisable that pet owners consult with a veterinarian or licensed pest control specialist [222].

The chemical control of other arthropods (e.g., kissing bugs, mosquitoes, and phlebotomine sand flies) in the environment is more complex and should also be performed in conjunction with public health authorities. For example, because mosquito larvae and pupae are strictly associated with water collections (e.g., containers, deactivated swimming pools, abandoned tyres), their population may be reduced and sometimes eradicated locally by destroying their breeding sites or by using biological control agents, such as the *Bacillus thuringiensis israelensis* and fishes such as bettas (*Betta splendens*) and guppies (*Poecilia reticulata*) [223,224]. The spraying of houses and animal shelters (e.g., chicken pen) with residual insecticides has long been recommended for controlling insect vectors, such as phlebotomine sand flies in Brazil [225,226]. However, results indicate that an effective vector population control may be difficult to achieve and resistance to insecticides currently in use in Brazil has been documented [227,228]. The use of insecticide-treated bed nets has also been proven to be effective in protecting humans against *L. longipalpis* bites [229], but this strategy is not currently used in Brazil as part of the national programme against zoonotic visceral leishmaniosis.

The control of common endoparasites of dogs (e.g., *A. caninum* and *T. canis*) and cats (e.g., *A. braziliense* and *T. cati*) usually consists of the routine administration of anthelminthics associated with vector control for those that are vector borne (e.g., *D. caninum*, *D. immitis*, and *P. praeputialis*); reviewed in Ref. [8]. Indeed, the control of zoonotic tapeworms (e.g., *E. granulosus*) may be effectively performed by the systematic use of praziquantel. For instance, a pilot control programme was conducted in southern Brazil, in which 44 rural dogs were treated monthly with praziquantel during 8 months [192]. At the beginning of the programme, 17 out 44 (28%) dogs were positive for *E. granulosus* coproantigens by ELISA and 30 days after the last treatment administration no dog was found positive [192]. Specifically, in the case of the double-pore tapeworm *D. caninum*, the control of fleas and lice is also advocated [8].

The level of awareness and compliance of pet owners may be low in some settings, particularly in outskirts and rural areas. Even if an important part of pet owners in Brazil cannot handle the costs of year-round preventive programmes, the lack of compliance of wealthy people to cope with veterinary recommendations is usually due to their reduced risk perception. Alternative strategies have been proposed, including the use of the nematophagous fungus *Pochonia chlamydosporia* as a biological control agent against embryonated eggs of *T. canis *[230]. Nevertheless, these alternatives are still a bit far from reality in Brazil.

Finally, the control of vector-borne parasites is usually based on the use of repellents on animals and on the control of arthropod vectors in the environment, whenever applicable [9,231]; e.g., for the control of *H. canis*, which is associated with high levels of environmental tick infestation [232,233]. However, other strategies such as dog culling have long been used in Brazil to control zoonotic visceral leishmaniasis, even if this strategy has been considered unethical and scientifically unsound [234]. Recently, the Minister Joaquim Barbosa, President of the Supreme Federal Court of Brazil, refused the request to suspend the judgment of the Interministerial Ordinance 1.426/2008, which prohibits the treatment and recommends the euthanasia of *L. infantum*-infected dogs (http://www.brasileish.com.br/dados/artigos/artigos38155712122013.pdf). The Minister stated that this Ordinance violates the Federal Constitution and that the public power should find alternatives to confront this issue, in partnership with scientists and veterinarians.

#### ***Prevention is better than cure***

Most dog owners seek the aid of a veterinary practitioner only when their animals display overt disease signs. This is particularly true in developing countries, where most dog owners cannot handle the costs of year-round preventive programmes. Naturally, in some rural and suburban areas the level of compliance may be even more reduced. Nonetheless, a considerable number of pet owners are increasingly aware of the risks and potential costs attached to the non-adoption of preventive measures directed at avoiding the exposure to certain pathogens, such as *L. infantum*. Thus, it is fundamental that veterinary practitioners recommend preventatives to pet owners towards the establishment of a long-term preventive programme against endoparasites and ectoparasites in Brazil.

The only vaccines against parasitic diseases available in Brazil are those against leishmaniosis, which are designed and approved against “canine leishmaniosis”, not against “*L. infantum* infection”. Indeed, none of the currently available vaccines against canine leishmaniosis will prevent dogs from becoming infected by *L. infantum*, but they should reduce the probability of vaccinated dogs to develop overt disease [231]. Importantly, if a vaccinated dog is able to mount an effective immune response against *L. infantum*, it will probably maintain the parasitic burden at low levels, eventually representing a poor source of parasites to phlebotomine vectors [235]. But again, it is important to emphasize that vaccines are not repellents and thus will not prevent dogs from being bitten by phlebotomine sand flies [235].

The use of repellents on dogs is an important strategy against ectoparasites and the pathogens they transmit [236-238]. Currently, there are several products formulated as spot-on solutions, collars, and sprays with fast killing effects, but synthetic pyrethroids remain the only chemical compounds with repellent properties available in the global veterinary market [239]. By virtue of their repellent and anti-feeding effects, synthetic pyrethroids (e.g., deltamethrin and permethrin) are highly efficacious against ectoparasites such as fleas, lice, sand flies, and ticks, but may also help to prevent the transmission of pathogens such as *B. vogeli*, *E. canis*, and *L. infantum *[236-238]. In the same way, some products with a fast killing effect are able to interrupt the attachment and blood feeding processes at their very early stages, thus also reducing the risk of pathogen transmission [240,241].

### Dogs, cats, parasites and humans: public health aspects

While some dog and cat ectoparasites are strictly associated to these animals, some of them may also infest humans. For instance, cases of human infestation by dog-associated ticks (e.g., *A. aureolatum*, *A. ovale*, and *R. sanguineus* s.l.) have sporadically been described in different Brazilian regions [242-247]. Cases of human infestations by dog ticks (*R. sanguineus* s.l.) appear to be associated with high levels of environmental infestation in human dwellings and with people working in veterinary clinics or kennels [242,244]. Importantly, some of these ticks may potentially transmit life-threatening pathogens to humans, including *Rickettsia rickettsii*[248]. For instance, *R. sanguineus* s.l. ticks have been implicated in the transmission of rickettsial organisms (e.g., *Rickettsia conorii*, *Rickettsia massiliae*, and *R. rickettsii*) to humans in the Mexico, the United States and the Mediterranean region [3]. Moreover, cases of human ehrlichiosis by *E. canis* have been described in Venezuela [249] and there is serological evidence that humans may be in jeopardy of being infected by *E. canis* in Brazil as well [250]. Importantly, *E. canis* is widespread and highly prevalent in Brazil [199,251-255], being primarily vectored by *R. sanguineus* s.l. ticks [256]. Cases of human infestation by *Ctenocephalides* spp. fleas have also been reported in Brazil [13,257], and it is now acknowledged that those fleas may carry *Rickettsia felis *[258], an emerging human pathogen worldwide [259]. It means that the control of some common dog and cat ectoparasites (e.g., *C. felis felis* and *R. sanguineus* s.l.) is fundamental not only for their health, but also to safeguard the health of their owners.

**Figure 5:**
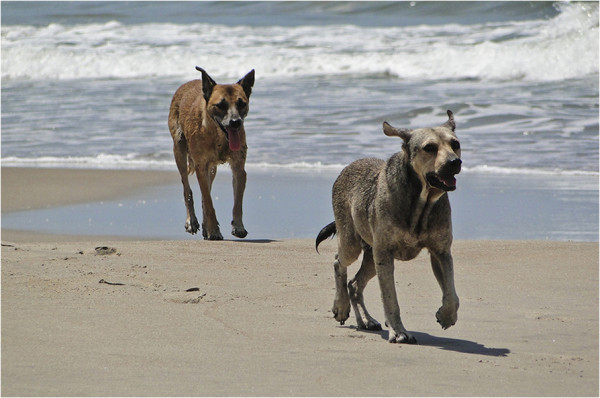
**Environmental contamination with dog faeces.** Stray dogs roaming freely in a beach in southern Brazil. Dog faeces are an important source of zoonotic parasites that may cause diseases such as cutaneous *larva migrans*.

Ascarids (*Toxocara* spp.) and hookworms (*Ancylostoma* spp.) of dogs and cats are parasites of great veterinary and public health relevance, even if the general perception of pet owners is that intestinal parasites of dogs and cats are of minor public health significance; reviewed in Ref. [260]. For instance, a study carried out in south-eastern Brazil revealed that most dog owners do not know the species of dog intestinal parasites, the mechanisms of transmission, the risk factors for zoonotic infections, and specific prophylactic measures [100]. Nonetheless, a number of cases of visceral and cutaneous *larva migrans* in humans have been described in Brazil [261-264] and serological data confirmed a high level of exposure to *Toxocara* spp. in humans in this country, reaching over 50% in some cities [265-269]. Accordingly, high levels of environmental contamination by *Toxocara* and *Ancylostoma* eggs in public parks and squares have been reported in different Brazilian regions [270-274]. Furthermore, human lagochilascariasis is an emerging zoonosis in the Americas caused by the roundworm *Lagochilascaris minor* and has been sporadically reported in Brazil [275-279], which holds the largest number of human cases reported in the literature [280]. Cats have been found naturally infested by *L. minor* and *Lagochilascaris major* in Brazil [281-283]. Indeed, the role of cats as reservoirs of *L. minor* has been speculated [284] and the contamination by *Lagochilascaris* eggs in public playgrounds has been documented in southern Brazil [285]. The soil contamination by dog and cat faeces is a common phenomenon in public gardens, sand boxes, and beaches (Figure 5) in Brazil, not only due to the large population of stray animals present in the cities, but also due to the lack of proper sanitary and health education of some pet owners.

Tapeworms are also an underestimated problem in Brazil and other South American countries. For instance, human cases of *D. caninum* have been described in Brazil [286-288]. Indeed, the high prevalence of *D. caninum* in dogs in Brazil (e.g., 45.7% in north-eastern and 36.8% in south-eastern Brazil) [32,36] is a consequence of the high prevalence of flea and lice infestations reported in different regions of the country [18,28,30,32,35] and indicates a high risk of zoonotic transmission in humans, particularly children, who live in close contact with infected animals. In the same way, cystic echinococcosis caused by *E. granulosus* is one of the most prevalent zoonosis in Argentina, Brazil, Chile, Peru, and Uruguay [289], being highly prevalent in southern Brazil, where up to 50% of the dogs may be infested [72,290]. While other canids (*Pseudalopex gymnocercus*, *Cerdocyon thous*, and *Chrysocyon brachyurus*) have been found naturally infested by *E. granulosus* in southern Brazil [290], dogs are the main reservoir of the parasite. In southern Brazil, *E. granulosus* is maintained principally in a dog-sheep cycle, with the sheep strain (G1) as the most prevalent genotype; reviewed in Ref. [290]. Furthermore, human cases of polycystic echinococcosis by *Echinococcus vogeli* have been diagnosed in northern Brazil [291] and dogs have also been suspected to play a role in the zoonotic transmission of this parasite in the Neotropical region [292]. Similarly, experimental evidence indicates that cats may act as definitive hosts for *E. oligarthrus *[293], another agent of polycystic echinococcosis in humans in Latin America [292], which is traditionally associated with wild felids. Last but not least, there is limited published information on other dog-associated taenids (e.g., *T. hydatigena* and *T. multiceps*) in Brazil [91], which suggests that human cysticercosis and coenuruses may be a silent, underestimated public health problem in some regions of this country.

Human pulmonary dirofilariosis is another common, yet underestimated public health issue in Brazil; reviewed in Ref. [294]. Even if several human cases have been described in the literature, most cases are accidentally found by chance on chest radiographies and chest computerized tomography scans, which are usually requested for other reasons [295]. Furthermore, even if most patients with pulmonary dirofilariosis present no apparent clinical signs, many of them will present cough, chest pain, haemoptysis, and dyspnoea [295-298]. Most importantly, the presence of pulmonary nodules could lead to the suspicion of diseases such as tuberculosis or lung cancer and in these cases thoracotomy is usually recommended. Indeed, human pulmonary dirofilariosis is a public health issue that has been so far neglected in Brazil. In the same way, the recent report of a case of ocular dirofilariosis in northern Brazil [299] suggests the risk of *Dirofilaria* infestation in certain Brazilian regions may be higher than currently appreciated.

Zoonotic protozoa are also a serious public health issue in Brazil. For instance, thousands of human cases of visceral leishmaniosis caused by *L. infantum* are notified each year to the Ministry of Health, but control measures mostly based on the elimination of seropositive dogs, vector control (in specific situations) and human treatment have not been enough to control the disease [300]. Indeed, human visceral leishmaniosis is widespread in all Brazilian regions, being less common in the southern part of the country [301]. The occurrence of asymptomatic *Leishmania* infections in humans has been documented in different regions of the country [302-304] and even if the Ministry of Health of Brazil affords the treatment of human patients, the lethality rate may reach 18.4% or much higher in some settings [305-307]. Another important zoonotic protozoan is *T. gondii*, an opportunistic pathogen highly prevalent in Brazil. Indeed, studies indicate that up to 50% of elementary school children and 50-80% of women of child-bearing age have anti-*T. gondii* antibodies; reviewed in Ref. [308]. A recent outbreak of acute toxoplasmosis in an industrial plant in São Paulo, south-eastern Brazil, indicated that ingestion of green vegetables (not meat or water) was associated with the incidence of acute disease [309]. Another recent study revealed a high seropositivity (80%) among HIV-positive patients from southern Brazil, some of which had a history of neurotoxoplasmosis (4.8%) and ocular toxoplasmosis (1.6%) [310].

Chagas disease caused by *T. cruzi* remains an important public health issue in the Americas, affecting about 10% of the poorest people in the region [311]. Dogs and cats are considered important reservoirs of *T. cruzi* in the domestic cycle of transmission [312]. For instance, studies indicate that around 15-50% of dogs living in areas where Chagas disease is endemic are seropositive [313-318]. However, the real role played by dogs and cats in the transmission cycle of *T. cruzi* in the current epidemiological context of Chagas disease in Brazil is arguable, because the great majority of recent human cases have been associated with oral transmission, via the ingestion of contaminated juices [319].

### Final considerations and future research directions

Brazil holds a vast territory and one of the largest dog and cat populations on the Planet. The economic growth and the increasing level of human development recorded in Brazil are provoking profound changes in the attitude of some dog owners and veterinary practitioners towards high-standard healthcare services. Nonetheless, the macroeconomic boom masks historical inequalities in terms of access to basic sanitation, decent housing, safe drinking water, health care and education at both local and regional levels. So, in reality, many dog owners cannot afford preventive measures and will act only when a life-threatening problem is affecting their animals. Furthermore, Brazil is still home to a large population of dogs and cats living as “strays” or “community” animals, not only in rural areas, but also in suburbs and large urban centres. Public health authorities are not able to manage these animals due to the lack of adequate infrastructure and trained personnel to conduct an effective long-term population control program. As a result, pet dogs and cats are usually endangered by a wide range of parasites that may cause disease in them and eventually in their human counterpart. This invariably brings us to the One Health concept, which invites veterinary practitioners and medical physicians to unify their efforts to combat zoonotic parasites (e.g., *T. canis*, *A. caninum*, *D. caninum*, *D. immitis*, *E. granulosus*, and *L. infantum*). Indeed, the unification of “veterinary and human medicines” in One Medicine is fundamental, instrumentally to reduce health hazards to pet animals and humans.

The outstanding diversity of ectoparasites infesting dogs and cats in Brazil is certainly a consequence of a combination of factors. The variety of natural environments and climates occurring in this country is impressive and favours the establishment of different parasites in all geographical regions. In the same way, dogs and cats living in either urban or rural areas may eventually live in close contact with livestock and wildlife species (e.g., foxes, wild cats, and opossums), which ultimately may favour the exchange of ectoparasites such as fleas and ticks with these animals [320-323]. For instance, *R. sanguineus* s.l. has been found on coati (*Nasua nasua*) living in a zoological park in a large urban area in north-eastern Brazil [322]. Even if this is an accidental finding, it suggests the presence of stray dogs circulating freely within the park’s territory and living in close contact with captive wild animals. Conversely, dogs and cats intruding or living near to forested environments may be found infested by wildlife-associated ectoparasites [12,28,50,321]. The exchange of ectoparasites between domestic and wild animals may also have implications from a conservational perspective, also because dogs may exchange endoparasites [324-326] and viruses [327,328] with wildlife species. For example, several wild canids (e.g., *C. thous* and *P. gymnocercus*) and felids (e.g., *Panthera onca*, *Puma concolor*, *Puma yagouaroundi*, and *Leopardus pardalis*) have been found harbouring parasites that may affect dogs and cats, including heminths (e.g., *A. caninum*, *D. caninum*, *T. canis*, and *T. vulpis*) and protozoa (e.g., *L. infantum*) [324-326,329-333]. Remarkably, while free-roaming wild canids infected with *L. infantum* are usually asymptomatic, captive ones seems to be more prone to develop clinical signs [329] and may even die from infection [332].

Veterinary practitioners working in Brazil are provided with a vast repertory of products with proven efficacy against dog and cat parasites. Indeed, the registration of veterinary products in Brazil involves several phases and strict regulatory requirements [334]. However, the success of any parasite control programme depends on the correct use of the chosen product(s) and on the adoption of the best strategy for each situation. As previously discussed, guidelines for the diagnosis, treatment and control of dog and cat parasites in Brazil are currently lacking and are urgently needed. In the meantime, veterinary practitioners working in Brazil should adhere strictly to manufacturers’ instructions while using commercially available products and follow international guidelines for the control of dog and cat parasites, eventually adapting them to regional situations. In addition, the Brazilian Ministry of Agriculture, Livestock and Food Supply recently established the use of a passport for international transit of dogs and cats (http://www.estadao.com.br/noticias/geral,caes-e-gatos-terao-passaporte-e-identificacao-eletronica,1099476,0.htm). This passport will be used as a health certification of origin and will include information about ecto- and endoparasite control. This will help to reduce the risk of unintentional exportation of parasites from Brazil to overseas.

There are many outstanding questions regarding parasites of dogs and cats in Brazil, some of which may have already been answered at the time that this manuscript is being drafted. For instance, it is important to understand why some parasites are apparently restricted to the southern and south-eastern regions of Brazil [9]. This is the case of *R. vitalii* and *B. gibsoni*, for example. While *B. gibsoni* is a relatively rare parasite in dogs in Brazil, *R. vitalii* is quite common in southern and south-eastern states and may cause severe disease that can be easily misdiagnosed with babesiosis or ehrlichiosis. Interestingly, the vectors of both protozoa remain unknown, but *Amblyomma aureolatum* is the putative vector for *R. vitalii*, at least. With this regard, the role of *R. sanguineus* s.l. in the natural cycle of *H. canis* in Brazil needs further investigation. Accordingly, other tick species have been implicated as putative vectors of this orally transmitted protozoan, including *A. ovale *[335,336] and *Rhipicephalus microplus *[337]. Some parasites of dogs and cats have only rarely been reported in Brazil, probably due to the limited research effort on these little studied parasites, whose veterinary and medical significance has yet to be ascertained. For instance, enigmatic parasites such as *Cercopithifilaria bainae *[338] have been originally described in Brazil, but remained doubtful and neglected for long time. Recently, this nematode was redescribed and it is emerging as a common filarioid of dogs in different European countries [339,340]. Altogether, these examples evidence that much has still to be discovered in Brazil regarding parasites of dogs and cats.

Further research on dog and cat parasites in Brazil may also reveal the existence of new species that have been overlooked over the years. Indeed, the use of molecular biology is revolutionizing in a positive fashion our knowledge on some “well-known” parasites, such as the brown dog tick *R. sanguineus* s.l. [52,161,162,341]. Likewise, genetic data suggested that nematodes identified as *A. vasorum* in dogs from South America may represent a different species from that observed in European dogs [342]. In this perspective, the use of molecular biology techniques should become a reality in the daily routine of veterinary practitioners working in Brazil and therefore the development of rapid, economically accessible molecular tools should be a research priority. For example, serological tests currently used by public authorities are not able to discriminate dogs infected by *L. infantum* from those infected by *L. braziliensis* or even distinguish vaccinated from naturally infected dogs [343]. Therefore, veterinary practitioners should request additional tests (e.g., parasite isolation and characterization, PCR and DNA sequencing) to confirm whether the dog is actually infected by *L. infantum* in order to avoid the elimination of dogs infected by other parasites and to decide about the most appropriate therapeutic approach to be adopted. With this respect, it is also important to monitor the emergence of resistance to certain active principles (e.g., amitraz, fipronil, deltamethrin, and permethrin), as these compounds have long been used for ectoparasite control in Brazil. Likewise, there is no information regarding the efficacy level of some endoparasiticides (e.g., praziquantel, pyrantel, febantel, and ivermectin), which are widely used in different Brazilian regions. These issues should be included in the research agenda of parasitologists in Brazil in order to anticipate problems related to parasite resistance to active principles currently used for controlling ectoparasites and endoparasites of dogs and cats in this country.

## Conclusions

In conclusion, the benefits of having a dog or a cat as a pet are undisputed, but the limit between benefit and prejudice is subtle. Indeed, the close contact between pets and humans may involuntarily represent a hazard for humans. Therefore, to avoid the potential risks associated to owning a pet dog or cat, it is fundamental to maintain them in good health and protected from zoonotic pathogens. Beyond the shadow of a doubt, it is our job as veterinarians to ensure that pets and their owners may live in harmony without representing a menace to each other. But from a holistic perspective, veterinary practitioners and medical physicians should work together towards improving the wellbeing and general health of both animals and humans. Paraphrasing a famous American singer, a One Health approach could help us to “heal the world” and “make it a better place” for dogs, cats, and humans.

## Competing interests

The authors declare that they have no competing interests.

## Authors’ contributions

FD-T wrote the manuscript and DO reviewed it critically. Both authors approved the final version of the manuscript.

## Supplementary Material

Additional file 1 Portuguese version of this review article. Click here for file
