# Erratum to: Dogs, cats, parasites, and humans in Brazil: opening the black box

**DOI:** 10.1186/s13071-016-1571-9

**Published:** 2016-05-23

**Authors:** Filipe Dantas-Torres, Domenico Otranto

**Affiliations:** Department of Immunology, Aggeu Magalhães Research Centre, Oswaldo Cruz Foundation, Recife, Pernambuco 50740465 Brazil; Department of Veterinary Medicine, University of Bari, Valenzano, Bari, 70010 Italy

## Erratum

Unfortunately, we found an error in Fig. 3 of our published paper [1]. The order of the images 3 L to 3P is not correct. The corrected version of Fig. 3 is included below. This correction also applies to the electronic supplementary material (Authors’ original file for Fig. 3 13071_2013_1729_MOESM4_ESM.tif).

**Fig. 3:**
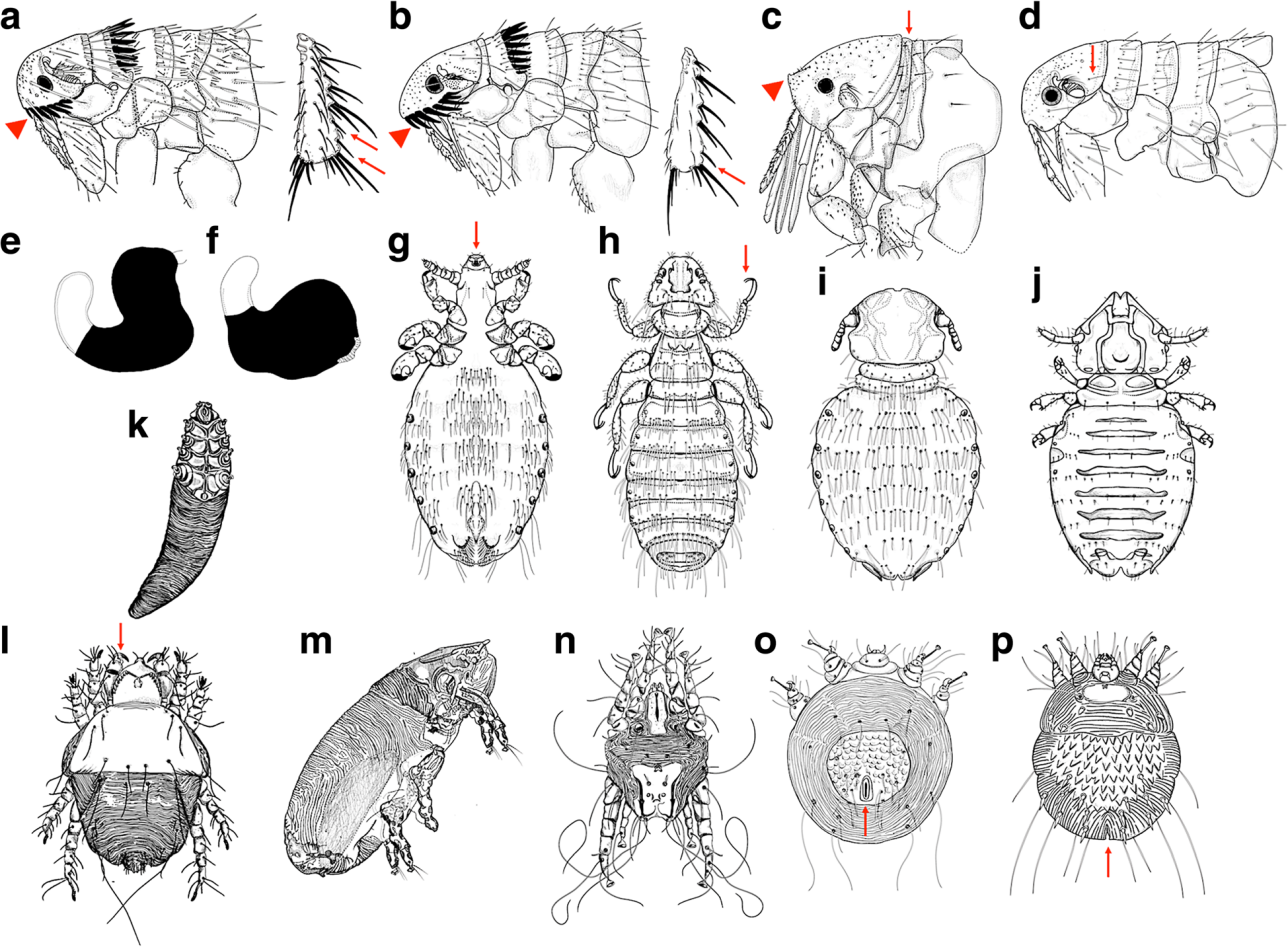
Ectoparasites of dogs and cats. Line drawings for the identification of common dog and cat ectoparasites (fleas: **a**–**f**; lice: **g**–**j** and mites: **k**–**p**) found in Brazil. For details see Table 3

We would like to apologise for this error and for any inconvenience this may have caused.
