# Supplementary material for: Dogs, cats, parasites, and humans in Brazil: opening the black box
Source: Parasit Vectors. 2014 Jan 14;7:22. doi: 10.1186/1756-3305-7-22 (PMC3914713; doi:10.1186/1756-3305-7-22)
Supplement: Additional file 1 — Portuguese version of this review article. [file 1756-3305-7-22-S1.pdf]

Dantas-Torres and Otranto Parasites & Vectors 2014, **7**:22
http://www.parasitesandvectors.com/content/7/1/22

Parasites & Vectors

Cães, gatos, parasitos e humanos no Brasil: abrindo a caixa preta

Filipe Dantas-Torres[1,2,*], Domenico Otranto[2]

[1] Departamento de Imunologia, Centro de Pesquisas Aggeu Magalhães, Fundação Oswaldo Cruz, Recife, Pernambuco 50670420, Brasil

[2] Departamento de Medicina Veterinária, Universidade de Bari, Valenzano, Bari 70010, Itália

Endereços de e-mail:

FD-T: filipe.vet@globo.com

DO: domenico.otranto@uniba.it

Resumo

Cães e gatos no Brasil são hospedeiros primários de um considerável número de parasitos, os quais podem afetar a saúde e o bem-estar desses animais. Esses parasitos incluem endoparasitos (e.g., protozoários, cestódeos, trematódeos e nematódeos) e ectoparasitos (e.g., pulgas, piolhos, ácaros e carrapatos). Enquanto alguns parasitos de cães e gatos são altamente específicos (e.g., *Aelurostrongylus abstrusus* e *Felicola subrostratus* para gatos e *Angiostrongylus vasorum* e *Trichodectes canis* para cães), outros podem facilmente mudar para outros hospedeiros, incluindo seres humanos. De fato, vários parasitos de cães e gatos (e.g., *Toxoplasma gondii*, *Dipylidium caninum*, *Ancylostoma caninum*, *Strongyloides stercoralis* e *Toxocara canis*) são importantes não apenas na perspectiva veterinária, mas também sob o ponto de vista médico. Ademais, alguns desses (e.g., *Lynxacarus radovskyi* em gatos e *Rangelia vitalii* em cães) ainda são pouco conhecidos para a maioria dos médicos veterinários que trabalham no Brasil. Este artigo é um compêndio sobre parasitos de cães e gatos no Brasil e uma chamada para uma abordagem de "Uma Saúde" (*One Health*) na busca de um melhor manejo desses parasitos, alguns dos quais podem afetar os humanos. Aspectos práticos relacionados ao diagnóstico, tratamento e controle de doenças parasitárias de cães e gatos no Brasil são discutidos.

Palavras-chave

Cães, Gatos, Humanos, Zoonose, Controle, América do Sul

Introdução

A palavra "parasito" (do Grego Antigo, *parasitos*: *para* = ao lado de, *sitos* = alimento) significa literalmente uma aquele que come na mesa de outro. No senso figurado, políticos

corruptos que fazem o uso do poder e dos recursos públicos para o seu próprio benefício poderiam ser chamados de “parasitos da sociedade”. Porém, em parasitologia, um parasito é um organismo que se beneficia de outro (o hospedeiro), sem dar algo em troca e usualmente causando algum dano a esse. A propósito, parasitos constituem um grupo diverso de organismos que podem afetar uma ampla variedade de hospedeiros, including anfíbios, aves, peixes, mamíferos e répteis. Eles podem ser geralmente subdivididos como endoparasitos (=internos) e ectoparasitos (=externos), de acordo com a sua localização no hospedeiro. Ectoparasitos também podem ser classificados como permanentes (e.g., piolhos e ácaros) ou não permanentes (e.g., carrapatos e mosquitos), dependendo da sua relação com o hospedeiro; isto é, se seu ciclo de vida acontece exclusivamente no hospedeiro ou também no ambiente.

Cães e gatos são os animais de estimação mais populares do mundo. Hoje em dia, cães têm assumido várias funções como guias para deficientes visuais, como agentes terapêuticos, como cães de guarda e de caça. Além disso, em muitos países em desenvolvimento, ambos os cães e gatos têm se tornado parte das famílias humanas, independentemente da classe social (Figura 1). Os benefícios de ter um animal de estimação são indiscutíveis [1], mas ambos os cães e gatos podem albergar muitos parasitos potencialmente transmissíveis ao homem, que podem representar um risco a saúde, especialmente de crianças, idosos e indivíduos imunocomprometidos [2]. Por exemplo, *Toxoplasma gondii*, *Dipylidium caninum*, *Echinococcus granulosus*, *Ancylostoma braziliense*, *Toxocara canis*, *Onchocerca lupi* e *Thelazia callipaeda* são parasitos comuns de cães e gatos que podem afetar humanos em diferentes países do mundo. Isso enfatiza a necessidade de uma abordagem de “Uma Saúde” (*One Health*) na busca de um melhor manejo e controle desses parasitos [3, 4].

No início do século XXI, as doenças parasitárias continuam causando um impacto significativo sobre as populações humanas e animais em regiões tropicais e subtropicais ao redor do mundo [5-7]. Mormente, o impacto de algumas dessas doenças é disproporcionalmente maior em países em desenvolvimento como o Brasil, onde as condições de vida das populações frequentemente favorecem a exposição a certos parasitos, cuja transmissão pode estar associada a pobres condições sanitárias e de moradia (Figura 2), assim como a inequidades no acesso a educação e a serviços primários de saúde.

O Brasil é um país de dimensões continentais (i.e., 8,515,767 km^2), ocupando a maior parte do território da América do Sul e representando a maior economia da região. Apesar das persistentes desigualdades sociais e econômicas (e.g., acesso desigual a educação, saneamento básico, assistência a saúde, água e habitação), que se tornaram o lado obscuro da história brasileira, a importância dos cães e gatos como animais de estimação, assim como a demanda por serviços veterinários de alto padrão, tem aumentado nas últimas décadas no Brasil. Incrivelmente, de acordo com a Associação Brasileira da Indústria de Produtos para Animais de Estimação (Abinpet), o Brasil possui atualmente a segunda maior população de cães e gatos do mundo, com mais de 37 milhões de cães e 21 milhões de gatos(http://abinpet.org.br/imprensa/mercado-pet-deve-faturar-r-154-bilhoes-em-2013). A propósito, o crescimento do número de cães e gatos em áreas urbanas no Brasil tem sido acompanhado por incrementos substanciais no mercado pet. De acordo com a Abinpet, o mercado pet brasileiro deve faturar 15,4 bilhões de reais (aproximadamente 7 billhões de dólares americanos) em 2013, com um crescimento global de 8,3% em comparação com 2012. Especificamente, estimou-se que em 2013 o setor de Pet Serv (serviços e cuidados com os animais) crescerá em 24,5%, Pet Food (alimento) em 4,9%, Pet Care (equipamentos, acessórios e produtos para higiene e beleza) em 5,2% e Pet Vet (medicamentos veterinários)

em 6,7%. Esses números colocam o Brasil como o segundo maior mercado pet do mundo, atrás apenas dos Estados Unidos.

A diversidade de ambientes naturais presentes no Brasil é incrível, assim como a sua biodiversidade. Por falar nisso, cães e gatos que vivem no Brasil podem albergar uma longa lista de parasitos que podem afetar o seu bem-estar e, eventualmente, constituir um risco para a saúde dos seus proprietários [8, 9]. Porém, as informações sobre a sua distribuição, epidemiologia e impacto desses parasitos na saúde humana são fragmentadas e principalmente publicadas em revistas brasileiras. Nesse sentido, esse artigo é um compêndio sobre os parasitos de cães e gatos no Brasil e uma chamada para uma abordagem de "Uma Saúde" (*One Health*) na busca de um melhor manejo desses parasitos, que podem potencialmente afetar os seres humanos. Aspectos práticos relacionados ao diagnóstico, tratamento e controle de doenças parasitárias de cães e gatos no Brasil são discutidos.

Revisão

Ectoparasitos de cães e gatos no Brasil

Ectoparasitos podem causar irritação, perda de sangue, prurido e lesões cutâneas, potencialmente levando a ocorrência de infecções bacterianas secundárias. Alguns ectoparasitos como pulgas, piolhos e carrapatos também podem transmitir patógenos aos cães e gatos, incluindo bactérias, protozoários e helmintos [revisado na Ref. 9]. Ademais, carrapatos podem também causar toxicose, como recentemente relatado em um cão picado pelo carrapato mouro *Ornithodoros brasiliensis* no Brasil [10].

No Brasil, cães e gatos podem servir como hospedeiros para um grande número de ectoparasitos, incluindo pulgas, piolhos, ácaros e carrapatos (Tabelas 1 e 2). Certamente, cães podem ser frequentemente infestados por pulgas (e.g., *Ctenocephalides canis*, *Ctenocephalides felis felis* e *Tunga penetrans*), piolhos (e.g., *Heterodoxus spiniger* e *Trichodectes canis*), ácaros (e.g., *Demodex canis*, *Otodectes cynotis* e *Sarcoptes scabiei*) e carrapatos (e.g., *Amblyomma aureolatum*, *Amblyomma ovale* e *Rhipicephalus sanguineus* sensu lato) [11-41]. Enquanto algumas pulgas podem ser comuns em ambientes rurais (e.g., *T. penetrans*), *C. felis felis* é certamente a pulga mais frequente em cães no Brasil. Outros ectoparasitos menos comuns (e.g., a pulga *Rhopalopsyllus lutzi lutzi*) têm sido esporadicamente relatados em cães no Brasil [21, 28]. De maneira similar, os achados de algumas espécies de carrapatos associadas de animais silvestres (e.g., *Amblyomma longirostre*, *Amblyomma naponsense*, *Amblyomma nodosum*, *Amblyomma pacae*, *Amblyomma scalpturatum* e *Haemaphysalis juxtakochi*) em cães no Brasil são achados incidentais e de significado clínico desconhecido.

Tal como acontece com os cães, gatos no Brasil são frequentemente encontrados infestados por pulgas (e.g., *C. canis*, *C. felis felis*, *P. irritans* e *T. penetrans*), piolhos (*Felicola subrostratus*) e ácaros (e.g., *D. cati*, *Lynxacarus radovskyi*, *Notoedres cati* e *O. cynotis*) [19, 22, 42-49]. Menos frequentemente, gatos têm sido encontrados infestados por carrapatos tais como *R. sanguineus* s.l. e *Amblyomma triste* [49-50]. Alguns desses ectoparasitos são bem conhecidos em algumas regiões (e.g., *L. radovskyi* no nordeste do Brasil), mas completamente desconhecidos ou considerados raros em outras. Mesmo que os gatos tenham mais conhecimento que cães quando o assunto é "remoção de ectoparasitos" por *grooming*,

eles podem ser altamentes expostos a ectoparasitos, particularmente em situações de confinamento em alta densidade, como acontece em gatis [45].

Embora a maioria das pulgas, piolhos, ácaros e carrapatos que infestam cães e gatos possa ser facilmente identificada, o delineamento de algumas espécies pode difícil para não taxonomistas [51, 52]. Embora algumas chaves para pulgas, piolhos e carrapatos tenham sido publicadas em livros [13, 14, 53] e revistas [54-56], não existem chaves pictóricas abrangentes para identificação de ectoparasitos de cães e gatos no Brasil (*vide* Tabela 3 e Figura 3).

Insetos voadores podem representar um problema clínico significante para cães e gatos no Brasil [9, 57-66]. Por exemplo, mosquitos, flebotomíneos e triatomíneos são vetores de *Dirofilaria immitis*, *Leishmania infantum* e *Trypanosoma cruzi*, respectivamente, para ambos os cães e gatos [9]. Miíase também é um problema muito comum em cães e gatos no Brasil, sendo mais frequentemente associada à infestação por larvas de *Cochliomyia hominivorax* e *Dermatobia hominis* [58-65] e menos comumente por outras espécies de moscas (e.g., *Lucilia eximia*) [57, 65]. Ademais, moscas-dos-estábulos (*Stomoxys calcitrans*) podem produzir lesões cutâneas nas orelhas desses animais, o que também pode levar a ocorrência de infecções bacterianas secundárias [9, 66].

Endoparasitos de cães e gatos no Brasil

Além de ectoparasitos, cães no Brasil podem ser acometidos por numerosos endoparasitos (Tabela 4), incluindo protozoários, trematódeos, cestódeos e nematódeos [32, 67-130]. Por exemplo, endoparasitos como *Dipylidium caninum*, *Echinococcus granulosus*, *A. braziliense*, *Ancylostoma caninum*, *Strongyloides stercoralis*, *Toxocara canis*, *Trichuris vulpis* e *Dirofilaria immitis* são comumente encontrados em cães de diferentes regiões brasileiras [67, 69]. Outros endoparasitos como *Angiostrongylus vasorum* tem sido apenas esporadicamente relatados no Brasil [74]. Similarmente, gatos têm sido frequentemente encontrados albergando vários endoparasitos (Tabela 5) no Brasil, incluindo *T. gondii*, *D. caninum*, *Taenia taeniformis*, *Spirometra mansonoides*, *Aelurostrongylus abstrusus*, *A. braziliense*, *A. caninum*, *A. tubaeforme*, *Toxascaris leonina*, *Toxocara cati*, *Pearsonema feliscati*, *Trichuris campanula*, *Trichuris serrata*, *Physaloptera praeputialis* e *Platynosomum fastosum* [47, 71, 87, 90, 92, 97, 131-137]. Endoparasitos menos comuns também têm sido relatados em gatos, tais como o acantocéfalo *Sphaerirostris erraticus* [137].

Cães tem sido contrados infectados por protozoários transmitidos por vetores, incluindo *Babesia gibsoni*, *Babesia gibsoni*, *Babesia vogeli*, *Rangelia vitalii*, *Hepatozoon canis*, *Leishmania amazonensis*, *Leishmania braziliensis*, *Leishmania infantum*, *Trypanosoma cruzi*, e *Trypanosoma evansi* [73, 78-82, 85, 87-90, 96, 102-104, 109-113, 117-121, 124, 128-130]. Outros protozoários de patogenicidade desconhecida (e.g., *Trypanosoma caninum*) também têm sido relatados em cães brasileiros [108]. Ademais, outros organismos transmitidos por vetores têm sido frequentemente relatados em cães, incluindo *Anaplasma platys*, *Ehrlichia canis* e menos frequentemente *Rickettsia rickettsii* [9].

Gatos também tem sido encontrados infectados por protozoários transmitidos por vetores tais como *B. vogeli*, *Cytauxzoon felis*, *H. canis*, *Hepatozoon felis*, *L. amazonensis*, *L. braziliensis*, e *L. infantum* [42, 44, 129, 136, 138-148]. Sem dúvida, a diversidade de endoparasitos de cães e gatos no Brasil é impressionante, mas certamente subestimada, principalmente porque a maioria dos estudos conduzidos nesse país se baseou na detecção e identificação de ovos e

oocistos em amostras de fezes usando técnicas coprológicas convencionais. Isso enfatiza a necessidade de técnicas diagnósticas adicionais (e.g., testes para detecção de coproantígenos, testes moleculares ou exame *post-mortem*) se quisermos conhecer a real diversidade e prevalência de endoparasitos de cães e gatos no Brasil.

Distribuição geográfica e prevalência

Parasitos de cães e gatos estão amplamente distribuídos no Brasil e na maioria dos casos ocorrem em virtualmente todas as regiões geográficas (norte, nordeste, sul, sudeste e centroeste) do país. Entretanto, para muitos parasitos externos (e.g., *D. canis*, *N. cati* e *O. cynotis*) e internos (e.g., *D. caninum*, *A. brasiliense* e *T. canis*) existe relativamente pouca informação publicada na literatura, particularmente em revistas internacionais. Consequentemente, é difícil no momento gerar mapas confiáveis e informativos para a maioria dos parasitos de cães e gatos que ocorrem no Brasil, mesmo que a maioria desses parasitos esteja certamente presente em todas as regiões do país. Por exmeplo, em um estudo realizado no sudeste do Brasil, 155 cães foram necropsiados e *D. caninum* foi diagnosticado em 57 (36,8%) deles, *A. caninum* em 30 (19,4%) e *T. canis* em 24 (15,5%) [36]. Similarmente, um estudo conduzido no nordeste do Brasil revelou que *A. caninum* (prevalência, 95,7%) foi o endoparasito mais comum em 46 cães errantes necropsiados, seguido por *D. caninum* (45,7%), *T. canis* (8,7%) e *T. vulpis* (4,3%) [32]. Esses estudos indicam altas taxas de prevalências de infestação por endoparasitos em cães errantes de diferentes regiões brasileiras representando uma fonte permanente de endoparasitos para animais de estimação e um grande risco zoonótico para humanos, por meio da contaminação de parques públicos e praias (*vide* "Cães, gatos, parasitos e humanos: aspectos de saúde pública").

Gatos errantes também são freqüentemente expostos a endoparasitos. Por exemplo, um estudo realizado no sudeste do Brasil relatou infestações por *A. braziliense* (67,3%), *A. caninum* (21,1%) e *A. tubaeforme* (9,6%) em 52 gatos necropsiados [116]. Da mesma forma, em um estudo recente realizado no centro-oeste do Brasil, 146 gatos foram necropsiados e 98 deles estavam infestados por nematódeos [*A. braziliense* (50,68%); *A. tubaeforme* (10,27%); *T. cati* (4,11%); *P. praeputialis* (2,05%); *P. feliscati* (3,42%) e *A. abstrusus* (1,37%)], cestódeos [*S. mansonoides* (4,11%); *D. caninum* (3,42%) e *T. taeniformis* (0,68%)], trematódeos [*P. fastosum* (26,03%)] e acantocéfalos [*S. erraticus* (3,42%)] [137]. Mais uma vez, esses estudos revelam uma diversidade considerável de endoparasitos em gatos de diferentes regiões do Brasil, com taxas relativamente elevadas de prevalência.

A distribuição geográfica dos protozoários e helmintos transmitidos por vetores de cães foi revista em outra revisão [9]. Por exemplo, *L. infantum* é amplamente distribuída em todas as regiões do Brasil [81, 113, 149, 150], mas menos freqüente no sul do país [151, 152]. No entanto, *L. longipalpis*, o principal vetor de *L. infantum*, está presente no norte da Argentina [153], no Paraguai [154] e foi detectado recentemente no Uruguai [155]. Além disso, *L. longipalpis* é abundante em Mato Grosso do Sul [156], que faz fronteira com o Estado do Paraná no sul do Brasil. Por último, mas não menos importante, a presença de *L. longipalpis* no Rio Grande do Sul (o estado brasileiro mais meridional) foi comprovada [157]. Esses resultados sugerem que *L. longipalpis* pode já ter colonizado outras áreas do sul do Brasil e que a leishmaniose canina pode se tornar um problema nessa região no futuro próximo.

O pouco conhecido protozoário *R. vitalii* ocorre principalmente nas regiões sul e sudeste do Brasil [119, 124, 158, 159], onde os carrapatos como *A. aureolatum*, o vetor suspeito, são

comumente encontrados em cães que freqüentam o ambiente de Mata Atlântica [160]. Curiosamente, *B. gibsoni* foi relatado exclusivamente no sul do Brasil [87, 104], o que também sugere que *R. sanguineus* s.l. não é o vetor deste protozoário no Brasil, considerando que este carrapato é prevalente em praticamente todas as regiões do país [52, 161, 162]. De fato, patógenos que são transmitidos por *R. sanguineus* s.l. (por exemplo, *B. vogeli* e *Ehrlichia canis*) geralmente apresentam ampla distribuição geográfica no Brasil [9], não concentrados em uma região específica, como ocorre com *B. gibsoni*. De qualquer modo, a possibilidade de que *R. sanguineus* s.l. esteja agindo como vetor de *B. gibsoni* no Brasil não pode ser descartada.

Pulgas do gênero *Ctenocephalides* também são amplamente distribuídas no Brasil [18, 35, 36, 38, 129], mas *C. felis felis* supera *C. canis* em distribuição geográfica, sendo essa última considerada mais comum em regiões com climas mais temperados, como no sudeste e sul do país [51]. De fato, *C. felis felis* foi relatada em 17 estados (Alagoas, Amazonas, Bahia, Ceará, Espírito Santo, Goiás, Mato Grosso, Minas Gerais, Paraíba, Paraná, Pernambuco, Rio de Janeiro, Rio Grande do Norte, Rio Grande do Sul, Roraima, Santa Catarina e São Paulo), enquanto *C. canis* tem sido relatada em apenas nove (Amazonas, Bahia, Maranhão, Minas Gerais, Paraná, Pernambuco, Rio Grande do Sul, Santa Catarina e São Paulo) [51]. Como conseqüência da ampla distribuição de pulgas (*Ctenocephalides* spp.) em cães e gatos, parasitos transmitidas por pulgas, como *D. caninum* e *A. reconditum* [163-167] também são comuns no Brasil [revisado na Ref. 9].

Mesmo que as pulgas, piolhos e carrapatos possam ser encontrados infestando cães em todas as regiões brasileiras, a distribuição e prevalência a nível local pode variar amplamente, também de acordo com as condições climáticas e do grau de urbanização de cada área. Por exemplo, em um estudo realizado no nordeste do Brasil, a frequência de infestação por *R. sanguineus* s.l. tendeu a ser maior em áreas urbanas do que nas áreas rurais, ao passo que as infestações por carrapatos do gênero *Amblyomma* e *C. felis felis* foram mais comuns em cães rurais [39]. No mesmo estudo, infestações mistas foram significativamente mais freqüentes em cães rurais do que em urbanos. No entanto, mesmo que *R. sanguineus* s.l. sejam mais prevalentes em ambientes urbanos [18, 28, 32], eles podem infestar uma grande proporção de cães rurais em algumas áreas [30, 33, 36].

Inquéritos sorológicos também relataram moderados a altos níveis de exposição a parasitos como *Neospora caninum* e *T. gondii* em cães e gatos no Brasil [151, 168-173]. Por exemplo, em um estudo recente realizado no sudeste do Brasil, 386 gatos foram analisados pelo teste de imunofluorescência indireta (IFI) para detectar a presença de anticorpos contra *T. gondii* e 63 (16,3%) deles foram positivos [172]. As taxas de prevalência de anticorpos anti-*N. caninum* em cães variando de 26% a 34,5% foram registradas no nordeste do Brasil, com 57,6 % dos cães soropositivos também positivos para anticorpos anti-*T. gondii* [169]. Em outro estudo realizado no sudeste do Brasil, 703 cães de áreas urbanas e rurais foram testados para *N. caninum* e 11,4 % deles foram positivos. A probabilidade de ser positivo para *N. caninum* foi maior em a cães com idade >4 anos, cães de guarda, cães de caça e cães de fazendas leiteiras com histórico de aborto bovino [173]. Em particular, entre os cães rurais, uma associação com a soroprevalência foi registrada em fazendas de leite, em cães não alimentados com ração comercial, em cães de caça e de guarda. Em conjunto, esses estudos revelam que os cães e gatos de diferentes regiões rurais e áreas urbanas estão em risco de infecção por parasitos, como *N. caninum* e *T. gondii*. Isto também é verdade para *Leishmania* spp. em cães, com taxas de soroprevalência superiores a 60% relatadas em algumas áreas de alta endemicidade [168].

Dinâmica sazonal nos trópicos

Informações sobre a ecologia dos parasitos de cães e gatos no Brasil são fragmentadas, limitadas, e, para vários parasitos, praticamente inexistentes. Considerando a variedade de biomas (Mata Atlântica, Amazônia, Pantanal, Cerrado, Caatinga e Pampa) e climas (do norte tropical até zonas temperadas abaixo do Trópico de Capricórnio) registrados no Brasil, é de se esperar que a dinâmica sazonal das populações de parasitos de cães e gato varie de acordo com a região. No entanto, as condições climáticas no Brasil são tão apropriadas que vários artrópodes vetores são encontrados durante todo o ano. Por exemplo, estudos realizados em uma área onde *D. immitis* ocorre de forma endêmica no Rio de Janeiro indicou que mosquitos vetores (e.g., *Ochlerotatus scapularis* e *Ochlerotatus taeniorhynchus*) são mais abundantes durante os meses de verão, mas também podem ser capturados em grande número nos meses de inverno [174]. Esses resultados sugerem que os cães e gatos podem estar sob o risco de infestação por *D. immitis* durante todo o ano, especialmente porque a temperatura média anual na maior parte do território brasileiro é de entre 21 e 30 °C, o que de fato facilita o desenvolvimento de larvas infectantes (L3) no mosquito vector.

A presença de ectoparasitos durante todo o ano no Brasil é aparentemente a regra, não a exceção. Flebotomíneos vetores de parasitos do gênero *Leishmania* tais como *L. longipalpis* (Figura 4) e *L. whitmani* podem ser encontrados em grande número ao longo do ano em diferentes regiões do Brasil [175-180], embora algumas espécies possam apresentar picos populacionais durante os meses de maior precipitação [181]. O mesmo se aplica para as pulgas e carrapatos. Um estudo recente desenvolvido no sudeste do Brasil revelou elevadas taxas de prevalência de infestações por pulgas (*C. felis felis*) e carrapatos (*A. cajennense* e *R. sanguineus* s.l.) durante as estações seca e chuvosa [35]. Outro estudo realizado na mesma região indicou que *R. sanguineus* s.l. pode infestar cães durante qualquer período do ano e completar até três gerações por ano [182]. Da mesma forma, uma investigação conduzida no centro-oeste Brasil sugeriu que esse carrapato pode completar até quatro gerações por ano [183]. Mais uma vez, estes resultados sugerem que diferentes regiões brasileiras possuem tipos de clima e condições ambientais que permitem a ocorrência de carrapatos durante todos os meses do ano. Por exemplo, embora um estudo tenha identificado variações na infecção por *B. vogeli* em cães aparentemente associadas a picos na população do carrapato vetor [184], as evidências indicam que esse protozoário pode infectar cães em qualquer período do ano em algumas regiões, independentemente da estação [120]. Por outro lado, miíases por *C. hominivorax* em cães e gatos apresenta um padrão sazonal bem definido no Brasil, atingindo o seu pico durante os meses mais quentes do ano [59-61, 65].

Os dados relativos a dinâmica sazonal de endoparasitos em cães e gatos no Brasil são escassos. Por exemplo, a incidência de larva migrans cutânea (*Ancylostoma* spp.) é maior durante a estação chuvosa [185]. Curiosamente, um estudo relatou um pico acentuado na excreção de ovos de *Ancylostoma* nas fezes de cães durante o verão e outono, sugerindo que as condições de inverno em São Paulo (sudeste do Brasil) têm um efeito negativo sobre a transmissão de ancilostomíase [74]. Outro estudo concluiu que a região sul do Brasil tem condições edáficas e climáticas favoráveis para que contaminantes do solo (e.g., ovos de *Toxocara*) persistam durante todo o ano, embora a diversidade de espécies de parasitos tenha sido menor no inverno e o número de parasitos menor no verão [186]. Esses achados estão de acordo com estudos realizados em outras partes da América do Sul. Por exemplo, um estudo realizado em Mar del Plata, Argentina, relatou uma alta prevalência de parasitos intestinais de cães ao longo do ano, com algumas variações sazonais de acordo com o gênero ou espécie de parasito [187]. Ancilostomíneos (*Ancylostoma* spp.) foram mais prevalentes no verão e no

outono, ascarídeos (*T. canis*) no inverno, enquanto tricurídeos (*T. vulpis*) atingiu o pico no inverno, primavera e verão. Mais uma vez, estes dados demonstram um alto risco de infestação parasitária em todas as estações nesta parte do continente americano.

Abordagens para o diagnóstico e manejo

Diagnóstico de doenças parasitárias

O diagnóstico das doenças parasitárias que afetam cães e gatos no Brasil ainda é predominantemente feito por métodos tradicionais. Por exemplo, os parasitos gastrointestinais são normalmente detectados por meio de técnicas coprológicas comuns, tais como as técnicas de Willis (flutuação em solução saturada de cloreto de sódio), Fausto (centrífugo-flutuação em sulfato de zinco) e Hoffman-Pons-Janer (sedimentação fecal espontânea em água) [188, 189]. Esses métodos podem apresentar baixa sensibilidade em alguns casos e resultar na subestimação da real prevalência de alguns parasitos, como *D. caninum* [74, 77, 100, 188, 190], quando comparados com os dados de necropsia [32]. Um teste comercial para concentração fecal (TF-Test®) concebido para a detecção de parasitos intestinais de humanos [191] também tem sido utilizado para a detecção de ovos de helmintos, cistos e oocistos de protozoários nas fezes de cães [190]. Um estudo comparativo revelou que a técnica de centrífugo-flutuação foi mais sensível que a centrífugo-sedimentação e que o TF-Test ® na detecção de *Ancylostoma* spp., *T. canis, T. vulpis* e *Giardia* em fezes de cães [190]. Outro estudo relatou que a técnica de Willis foi mais eficiente na detecção de ovos de *A. caninum* e *T. canis* nas fezes de cães [188]. Como corolário, um estudo mais recente demonstrou que as técnicas de Willis e de centrífugo-flutuação apresentaram um melhor desempenho do que a técnica de Hoffman-Pons-Janer para detectar *Ancylostoma* spp. em fezes de cães [189]. Também foram propostos outros métodos, mas, aparentemente, não havendo diferença significativa em termos de sensibilidade, em comparação com métodos tradicionais [98]. O uso de um ensaio imunoenzimático (ELISA) para a detecção de coproantígenos de *E. granulosus* revelou altas taxas de positividade (27,69–47,37%) em cães rurais do sul do Brasil [192]. Da mesma maneira, técnicas baseadas na reação em cadeia da polimerase (PCR) foram utilizadas para detectar endoparasitos (helmintos e protozoários) de cães no Brasil [83, 126, 193, 194], mas atualmente esses métodos estão restritos principalmente à pesquisa.

Hematozoários (e.g., *B. vogeli* e *H. canis*) são normalmente diagnosticados por exame de esfregaços de sangue corados sob um microscópio de luz, método essse que pode apresentar baixa sensibilidade, especialmente quando as amostras de sangue não são coletadas na fase aguda da infecção [195]. Os testes sorológicos são amplamente utilizados para avaliar a exposição a patógenos como *B. vogeli*, *Leishmania* spp., *N. caninum* e *T. gondii* [168, 169, 184, 196-198]. O uso de ferramentas moleculares para o diagnóstico de protozoários parasitos (e.g., *B. vogeli*, *H. canis* e *L. infantum*) está se tornando cada vez mais popular, mas continua mais restrito à pesquisa [121, 199-204]. Certamente, os protocolos de PCR atuais têm mostrado um bom nível de concordância com os métodos parasitológicos [150]. Infelizmente, os custos de ferramentas moleculares ainda são proibitivos para a maioria dos proprietários de animais que vivem em áreas endêmicas e isso prejudica severamente o diagnóstico de doenças como a leishmaniose visceral e cutânea em cães. De fato, as ferramentas sorológicas atuais não conseguem distinguir entre *L. braziliensis* e *L. infantum* [113, 205]. Esse fato pode ter implicações diretas para médicos veterinários no Brasil uma vez que os cães soropositivos são normalmente eliminados como parte do programa de controle da leishmaniose visceral humana, embora tenha sido demonstrado que muitos destes cães soropositivos estão

infectados por *L. braziliensis* e não por *L. infantum* [113 , 205, 206]. Na verdade, a grande diversidade de parasitos no Brasil pode ser um fator de confusão no diagnóstico de algumas doenças em cães e gatos no país. Por exemplo, diferentes espécies de *Leishmania* foram relatadas nestes animais no Brasil e o quadro clínico não é sempre previsível. Um relato recente descreveu um caso de leishmaniose canina por *L. infantum* em que o cão apresentava uma úlcera cutânea que é normalmente encontrado em cães e seres humanos infectados por *L. braziliensis* [207]. Mais uma vez, cães infectados por *L. amazonensis* geralmente exibem sinais clínicos comumente observados em cães com leishmaniose visceral [96, 117]. Por outro lado, os gatos infectados por *L. amazonensis* exibem tipicamente uma doença cutânea, que se assemelha a esporotricose [141, 208]. Isso significa que o mesmo parasito pode eventualmente induzir doenças clínicas diferentes em cães e gatos, o que enfatiza a necessidade de um diagnóstico laboratorial adequado para confirmar as espécies de parasitos envolvidos. Isso também é verdade para os ovos de ascarídeos (*Toxocara* spp.) e tenídeos (*Taenia* spp. e *Echinococcus* spp.) encontrados nas fezes de cão, os quais normalmente não podem ser diferenciados através da microscopia de luz [209, 210]. Ovos de ancilóstomídeos não são facilmente distinguíveis pela morfologia, mas os dados morfométricos sugerem que eles podem ser diferenciados [211]. No entanto, o uso de técnicas diagnósticas aperfeiçoadas e mais específicas, é fundamental para uma melhor gestão das doenças parasitárias de cães e gatos no Brasil.

Controlando parasitos de cães e gatos

Médicos veterinários que atuam no Brasil dispõem de um repertório relativamente vasto de ferramentas para controlar os parasitos que afetam cães e gatos. No entanto, o controle de parasitos comuns, como pulgas e carrapatos podem parecer bastante difícil, principalmente quando as ferramentas disponíveis não são utilizadas devidamente ou quando médicos veterinários não possuem conhecimentos básicos sobre a bio-ecologia de alguns desses parasitos. No Brasil, não existem diretrizes para o controle de ecto- e endoparasitos de cães e gatos, tais como aquelas fornecidas pelo Conselho Científico Europeu para os Parasitos de Animais de Companhia (ESCCAP: http://www.esccap.org/) na Europa e pelo Conselho de Combate aos Parasitos em Animais (CAPC: http://www.capcvet.org/) nos Estados Unidos. Essas diretrizes são fundamentais para garantir aos médicos veterinários o acesso as informações científicas mais recentes sobre o controle de parasitos de cães e gatos.

Porque os piolhos e ácaros vivem em associação permanente com os seus hospedeiros, eles são relativamente fácil de controlar e, uma vez eliminados, a reinfestação só ocorre após o contato com outros animais infestados [212, 213]. Portanto, o controle de infestações por piolho em cães e gatos é principalmente baseado na aplicação de colares, soluções *spot-on* e sprays com produtos inseticidas sobre os animais infestados. Esses inseticidas podem estar disponíveis como [214-216]. Ácaros também são ectoparasitos permanentes, com alguns deles vivendo no pêlo (*L. radovskyi*), outros no conduto auditivo externo (*O. cynotis*), nos folículos pilosos e glândulas sebáceas (*D. canis* e *D. cati*), ou mesmo em galerias escavadas sob a epiderme (*N. cati* e *S. scabiei*). O tratamento das infestações por ácaros pode ser sistêmico (e.g., selamectina oral para *S. scabiei*) ou tópico (e.g., banhos de amitraz para *D. canis*). Por exemplo, o tratamento de infestações pelo ácaro do pêlo do gato (*L. radovskyi*) no Brasil tem sido realizado com sucesso com banhos semanais com um sabão de monossulfeto de tetraetiltiuram [217]. Do mesmo modo, uma única injecção subcutânea de doramectina 1% (0,3 mg/kg) foi eficaz no tratamento de cães com sarna sarcóptica e com endoparasitos [218]. No entanto, o tratamento da demodicose em cães pode variar dependendo da gravidade do caso, a idade do animal, bem como a presença de uma doença de base [*vide* Ref. 219]. Na

verdade, a maioria das lesões em cães jovens com demodicose juvenil localizada resolverá espontaneamente sem tratamento, ao passo que cães adultos com demodicose generalizada geralmente requerem terapia mais agressiva, por exemplo, com banhos de amitraz a cada duas semanas durante vários meses [219]. Além disso, uma doença de base ou condição de saúde (e.g., câncer, hiperadrenocorticismo, uso de drogas imunossupressoras) deve sempre ser investigada e tratada em conformidade, especialmente em casos de demodicose em cães adultos. É evidente que o tratamento da sarna em cães e gatos depende de vários factores relacionados com as espécies de ácaros envolvidas e com as características de cada doente individual. Médicos veterinários devem usar os produtos rotulados especificamente para o tratamento da sarna em cães e gatos no Brasil e seguir rigorosamente as recomendações do fabricante.

Em contraste com os ácaros e piolhos, carrapatos e pulgas são parasitos não-permanentes [212] e, portanto, exemplares encontrados em cães e gatos podem representar apenas uma pequena proporção de toda a população de pulgas e carrapatos presentes no ambiente onde vivem os animais infestados. Um vasto repertório de produtos está disponível no mercado veterinário brasileiro para o controle de pulgas e carrapatos em cães e gatos. Inseticidas e acaricidas com potentes efeitos *anti-feeding* e de *killing*, repelentes, reguladores de crescimento de insetos e análogos do hormônio juvenil estão disponíveis como pipetas (*spot-on*), coleiras, xampus, sabonete, talco, sprays e comprimidos [212, 220]. No entanto, antes de decidir a melhor estratégia de controle a ser adotada, o médico veterinário também deve investigar a presença e a abundância de pulgas/carrapatos no ambiente onde o animal vive. Carrapatos tendem a cair durante a noite, quando o cão está dormindo [221]. Ademais, os ovos das pulgas não são aderentes, caindo do pêlo do animal no ambiente junto com as fezes de pulgas adultas. Larvas recém-eclodidas geralmente evitam a luz solar e se movem ativamente entres as fibras de tapetes ou sob detritos orgânicos [222]. Aspirar lugares freqüentados por animais (e.g., carpetes, almofadas, tapetes, móveis ou outros substratos), com um aspirador de pó, bem como lavar a roupa de cama do animal de estimação ou roupa de cama freqüentada por esses irá ajudar na remoção de ovos e larvas de pulgas [222]. Além disso, tratamentos do ambiente interno e externo com insecticidas e acaricidas podem ser necessários em algumas situações, tais como a canis e gatis com níveis elevados de infestação ambiental por pulgas e de carrapatos. Cuidados devem ser tomados ao utilizar produtos químicos no ambiente e é aconselhável que os proprietários consultem um veterinário ou especialista autorizado em controle de pragas [222].

O controle químico de outros artrópodes (e.g., barbeiros, mosquitos e flebotomíneos) no ambiente é mais complexo e também deve ser realizado em conjunto com as autoridades de saúde pública. Por exemplo, uma vez que larvas e pupas de mosquitos são estritamente associadas com coleções de água (e.g., *containers*, piscinas desativadas, pneus abandonados), sua população pode ser reduzida e, às vezes erradicada localmente, por meio da destruição de seus locais de reprodução ou por meio de agentes de controle biológico, tais como o *Bacillus thuringiensis israelensis* e peixes como bettas (*Betta splendens*) e guppies (*Poecilia reticulata*) [223, 224]. A pulverização de casas e abrigos de animais (e.g., galinheiros) com inseticidas de ação residual tem sido recomendada para o controle de insetos vetores, como flebotomíneos no Brasil [225, 226]. No entanto, os resultados indicam que um controle efetivo da população do vetor pode ser difícil de alcançar e resistência a inseticidas atualmente em uso no Brasil tem sido documentada [227, 228]. O uso de mosquiteiros tratados com insecticida também foi demonstrado ser eficaz na proteção de seres humanos contra picadas de *L. longipalpis* [229], mas esta estratégia não é usada atualmente no Brasil, como parte do programa nacional contra a leishmaniose visceral zoonótica.

O controle de endoparasitos comuns de cães (e.g., *A. caninum* e *T. canis*) e gatos (e.g., *A. braziliense* e *T. cati*) geralmente consiste na administração de anti-helmínticos de rotina associados ao controle de vetores para aqueles que são transmitidas por vetores (e.g., *D. caninum*, *D. immitis* e *P. praeputialis*); revisado na Ref. [8]. De fato, o controle de helmíntos com potencial zoonótico (e.g., *E. granulosus*) pode ser efetivamente realizado pelo uso sistemático de praziquantel. Por exemplo, um programa de controle de piloto foi realizado no sul do Brasil, em que 44 cães rurais foram tratados mensalmente com praziquantel, durante oito meses [192]. No início do programa, 17 de 44 (28%) cães foram positivos para coproantígenos de *E. granulosus* por ELISA e 30 dias após a última administração no tratamento nenhum cão foi positivo [192]. Especificamente, no caso do cestódeo *D. caninum*, o controle de pulgas e piolhos também deve ser preconizado [8].

O nível de consciência e de *compliance* dos proprietários de animais pode ser baixos em alguns contextos, especialmente na periferia e em áreas rurais. Embora uma parte importante dos proprietários de animais no Brasil não consiga arcar com os custos dos programas de prevenção durante todo o ano, a falta de cumprimento das recomendações veterinárias por parte de pessoas com maior poder aquisitivo é geralmente devida a falta de informação e/ou reduzida percepção de risco. Estratégias alternativas têm sido propostas, incluindo o uso do fungo nematófago *Pochonia chlamydosporia* como agente de controle biológico contra ovos embrionados de *T. canis* [230]. Contudo, essas alternativas ainda parecem um pouco longe da realidade no Brasil.

Finalmente, o controle de parasitos transmitidos por vetores é usualmente baseado na utilização de repelentes nos animais e no controle dos artrópodes vetores no ambiente, sempre que aplicável [9, 231], por exemplo, para o controle de *H. canis*, o qual é associado a altos níveis de infestação ambiental por carrapatos [232, 233]. No entanto, outras estratégias, como a eliminação de cães têm sido utilizadas no Brasil para o controle da leishmaniose visceral zoonótica, mesmo sendo considerada uma estratégia antiética e sem base científica [234]. Recentemente, o Ministro Joaquim Barbosa, presidente do Supremo Tribunal Federal do Brasil, recusou o pedido de suspensão do julgamento da Portaria Interministerial 1.426/2008 que proibia o tratamento e recomendava a eutanásia de cães infectados por *L. infantum* (www.brasileish.com.br/dados/artigos/artigos38155712122013.pdf). O Ministro declarou que essa Portaria viola a Constituição Federal e que o poder público deveria encontrar alternativas para enfrentar essa questão, em parceria com cientistas e veterinários.

A prevenção é melhor que a cura

A maioria dos proprietários de cães procura a ajuda de um médico veterinário apenas quando os seus animais mostram sinais evidentes de doença. Isso é particularmente verdadeiro nos países em desenvolvimento, onde a maioria dos proprietários de cães não pode lidar com os custos dos programas de prevenção durante todo o ano. Naturalmente, em algumas áreas rurais e suburbanas do nível de adesão as recomendações veterinárias pode ser ainda mais reduzido. No entanto, um número considerável de proprietários de animais está cada vez mais consciente dos riscos e custos potenciais associados a não adoção de medidas preventivas no sentido de evitar a exposição a certos patógenos, tais como *L. infantum*. Assim, é fundamental que médicos veterinários recomendem preventivos para proprietários de animais no intuido de estabelecer um programa preventivo de longo prazo contra endoparasitos e ectoparasitos no Brasil.

As únicas vacinas contra doenças parasitárias disponíveis no Brasil são aquelas contra a leishmaniose, que são concebidas e aprovadas contra a “leishmaniose canina” e não contra “a infecção por *L. infantum*”. Na verdade, nenhuma das vacinas atualmente disponíveis contra leishmaniose canina impedirá que os cães sejam infectados por *L. infantum*, mas podem reduzir a probabilidade de adoecimento em cães vacinados [231]. De maneira importante, se um cão vacinado for capaz de desenvolver uma resposta imune eficaz contra *L. infantum*, ele irá provavelmente manter a carga parasitária reduzida, representando, eventualmente, uma fonte pobre de parasitos para os flebotomíneos vetores [235]. Porém, é importante ressaltar que as vacinas não são repelentes e, portanto, não irão impedir que os cães de serem picados por flebotomíneos [235].

O uso de repelentes nos cães é uma estratégia importante contra ectoparasitos e contra os patógenos que eles transmitem [236-238]. Atualmente, existem vários produtos ectoparasiticidas (e.g., pipetas, coleiras e sprays) com rápido efeito de *killing*, mas os piretróides sintéticos continuam a ser os únicos compostos químicos com propriedades repelentes disponíveis no mercado veterinário mundial [239]. Em virtude dos seus efeitos repelentes e de anti-*feeding*, os piretróides sintéticos (e.g., deltametrina e permetrina) são altamente eficazes contra ectoparasitos como pulgas, piolhos, flebotomíneos e carrapatos, mas também podem ajudar a prevenir a transmissão de patógenos como *B. vogeli*, *E. canis* e *L. infantum* [236-238]. Da mesma forma, alguns produtos com um rápido efeito de *killing* são capazes de interromper os processos de fixação e alimentação nas suas fases iniciais, reduzindo também o risco de transmissão de patógenos [240, 241].

Cães, gatos, parasitos e humanos: aspectos de saúde pública

Enquanto alguns ectoparasitos de cães e gatos são estritamente associados a esses animais, alguns deles também podem infestar seres humanos. Por exemplo, casos de infestação humana por carrapatos de cães (e.g., *A. aureolatum*, *A. ovale* e *R. sanguineus* s.l.) esporadicamente têm sido descritos em diferentes regiões do Brasil [242-247]. Os casos de infestações humanas pelo carrapato marrom do cão (*R. sanguineus* s.l.) parecem estar associados com altos níveis de infestação ambiental em habitações humanas e com indivíduos que trabalham em clínicas veterinários ou canis [242, 244]. De maneira importante, alguns destes carrapatos podem transmitir patógenos potencialmente letais para os seres humanos, incluindo *Rickettsia rickettsii* [248]. Por exemplo, *R. sanguineus* s.l. tem sido implicado na transmissão de riquétsias (e.g., *Rickettsia conorii*, *Rickettsia massiliae* e *R. rickettsii*) para os seres humanos no México, nos Estados Unidos e na região Mediterrânea [3]. Além disso, casos de erliquiose humana por *E. canis* foram descritos na Venezuela [249] e evidências sorológicas indicam que os humanos podem estar expostos ao risco de infecção por *E. canis* no Brasil [250]. É importante destacar que *E. canis* é amplamente distribuída e altamente prevalente no Brasil [199, 251-255], sendo transmitida primariamente por *R. sanguineus* s.l. [256]. Casos de infestação humana por *Ctenocephalides* spp. também têm sido relatados no Brasil [13, 257] e atualmente é sabido que essas pulgas podem albergar *Rickettsia felis* [258], um patógeno humano emergente em todo o mundo [259]. Isso significa que o controle de alguns ectoparasitos comuns de cães e gatos (e.g., *C. felis felis* e *R. sanguineus* s.l.) é fundamental não só para a saúde desses animais, mas também para proteger a saúde dos seus proprietários.

Ascarídeos (*Toxocara* spp.) e ancilostomídeos (*Ancylostoma* spp.) de cães e gatos são parasitos de grande relevância veterinária e para a saúde pública, mesmo considerando que a percepção geral dos proprietários de animais seja de que os parasitos intestinais de cães e

gatos são de importância menor para a saúde pública; revisado na Ref. [260]. Por exemplo, um estudo realizado no sudeste do Brasil revelou que a maioria dos proprietários de cães não conhecem as espécies de parasitos intestinais de cães, os mecanismos de transmissão, os fatores de risco para infecções zoonóticas e medidas profiláticas específicas [100]. No entanto, inúmeros casos de larva migrans visceral e cutânea em seres humanos têm sido descritos no Brasil [261-264] e os dados sorológicos confirmaram um nível elevado de exposição a *Toxocara* spp. em seres humanos no país, atingindo mais de 50% em algumas cidades [265-269]. Assim, altos níveis de contaminação ambiental por ovos de *Toxocara* e *Ancylostoma* em parques e praças públicas têm sido relatados em diferentes regiões do Brasil [270-274]. Além disso, lagochilascaríase humana é uma zoonose emergente nas Américas causada pelo nematódeo *Lagochilascaris minor* e tem sido esporadicamente relatada no Brasil [275-279], país que detém o maior número de casos humanos relatados na literatura [280]. Os gatos têm sido encontrados naturalmente infestados por *L. minor* e *Lagochilascaris major* no Brasil [281-283]. Na verdade, o papel dos gatos como reservatórios de *L. minor* tem sido especulado [284] e a contaminação por ovos de *Lagochilascaris* em playgrounds públicos foi documentada no sul do Brasil [285]. A contaminação do solo por fezes de cães e gatos é um fenômeno comum em jardins públicos, caixas de areia e praias (Figura 5) no Brasil, não só devido à grande população de animais errantes presentes nas cidades, mas também devido à falta de educação sanitária e de saúde de alguns proprietários de animais.

Os cestódeos também são um problema subestimado no Brasil e em outros países sul-americanos. Por exemplo, os casos humanos de *D. caninum* foram descritos no Brasil [286-288]. De fato, a alta prevalência de *D. caninum* em cães no Brasil (e.g., 45,7% no nordeste e 36,8% no sudeste do Brasil) [32, 36] é uma conseqüência da alta prevalência de infestações por pulgas e piolhos relatadas em diferentes regiões do país [18, 28, 30, 32, 35] e indica um alto risco de transmissão zoonótica para humanos, particularmente crianças que vivem em estreito contato com animais infestados. Da mesma forma, a equinococose cística causada por *E. granulosus* é uma das zoonoses de maior prevalência na Argentina, Brasil, Chile, Peru e Uruguai [289], sendo altamente prevalente no sul do Brasil, onde até 50% dos cães podem estar infestados [72, 290]. Embora outros canídeos (*Pseudalopex gymnocercus, Cerdocyon thous* e *Chrysocyon brachyurus*) tenham sido encontrados naturalmente infestados por *E. granulosus* no sul do Brasil [290], os cães são os principais reservatórios do parasito. No sul do Brasil, *E. granulosus* é mantido principalmente em um ciclo que envolve cães e ovelhas, sendo a cepa ovina (genótipo G1) a mais prevalente; revisado na Ref. [290]. Além disso, casos humanos de equinococose policística por *Echinococcus vogeli* foram diagnosticados no norte do Brasil [291] e suspeita-se que os cães desempenhem um papel na transmissão zoonótica deste parasito na região Neotropical [292]. Da mesma forma, evidências experimentais indicam que os gatos podem atuar como hospedeiros definitivos para *E. oligarthrus* [293], um outro agente da equinococose policística em humanos na América Latina [292], que é tradicionalmente associado a felinos selvagens. Por último, mas não menos importante, há limitadas informações publicadas em outros tenídeos associadas ao cão (e.g., *T. hydatigena* e *T. multiceps*) no Brasil [91], o que sugere que a cisticercose e cenurose humana pode ser um problema silêncioso e subestimado de saúde pública em algumas regiões do país.

A dirofilariose pulmonar humana é outro problema de saúde pública comum, ainda que subestimado no Brasil; revisado na Ref. [294]. Embora vários casos humanos tenham sido descritos na literatura, a maioria deles é acidentalmente encontrada por acaso em radiografias de tórax e tomografia computadorizada de tórax, que geralmente são solicitados por outras razões [295]. Além disso, embora a maioria dos pacientes com dirofilariose pulmonar não

apresentam sinais clínicos aparentes, muitos deles irão apresentar tosse, dor torácica, hemoptise e dispnéia [295-298]. Mais importante ainda, a presença de nódulos pulmonares podem levar a suspeita de doenças como a tuberculose ou câncer de pulmão e, nesses casos, a toracotomia é geralmente recomendada. De fato, dirofilariose pulmonar humana é uma questão de saúde pública que tem sido até agora negligenciada no Brasil. Da mesma forma, o relato recente de um caso de dirofilariose ocular no norte do Brasil [299] sugere que o risco de infestação por *Dirofilaria* spp. em certas regiões do Brasil pode ser maior do que atualmente reconhecido.

Protozoários zoonóticos também são uma questão de saúde pública grave no Brasil. Por exemplo, milhares de casos humanos de leishmaniose visceral causada por *L. infantum* são notificados todos os anos ao Ministério da Saúde, mas as medidas de controle focalizadas na eliminação de cães soropositivos, controle de vetores (em situações específicas) e tratamento humano não tem sido suficientes para controlar a doença [300]. De fato, a leishmaniose visceral humana é amplamente distribuída em todas as regiões brasileiras, sendo menos comum na região sul do país [301]. A ocorrência das infecções assintomáticas por *Leishmania* em humanos tem sido documentada em diferentes regiões do país [302-304] e embora o Ministério da Saúde forneça o tratamento aos pacientes humanos, a taxa de letalidade pode atingir 18,4% ou valores muito maiores em algumas localidades [305-307]. Outro importante protozoário zoonótico é *T. gondii*, um patógeno oportunista altamente prevalente no Brasil. De fato, estudos indicam que até 50% das crianças do ensino fundamental e 50-80% das mulheres em idade fértil têm anticorpos anti-*T. gondii*; revisado na Ref. [308]. Um recente surto de toxoplasmose aguda em uma planta industrial em São Paulo, sudeste do Brasil, indicou que a ingestão de vegetais verdes (não de carne ou água) foi associada com a incidência de doença aguda [309]. Outro estudo recente revelou uma soropositividade elevada (80%) entre os pacientes HIV-positivos do sul do Brasil, alguns dos quais tinham uma história de neurotoxoplasmose (4,8%) e da toxoplasmose ocular (1,6%) [310].

A doença de Chagas causada por *T. cruzi* continua a ser um importante problema de saúde pública nas Américas, afetando cerca de 10% das pessoas mais pobres da região [311]. Cães e gatos são considerados importantes reservatórios de *T. cruzi* no ciclo doméstico de transmissão [312]. Por exemplo, os estudos indicam que 15-50% dos cães que vivem em áreas onde a doença de Chagas é endêmica são soropositivos [313-318]. No entanto, o verdadeiro papel desempenhado pelos cães e gatos no ciclo de transmissão do *T. cruzi* no contexto epidemiológico atual da doença de Chagas no Brasil é discutível, pois a grande maioria dos casos humanos recentes tem sido associada com a transmissão oral, através da ingestão de sucos contaminados [319].

Considerações finais e futuras pesquisas

O Brasil ocupa um vasto território e possui uma das maiores populações de cães e gatos no Planeta. O crescimento econômico e o aumento do nível de desenvolvimento humano registrado no Brasil estão provocando mudanças profundas na atitude de alguns proprietários de cães e veterinários na busca de padrões mais elevados de serviços de saúde. No entanto, o *boom* macroeconômico mascara desigualdades históricas no acesso ao saneamento básico, habitação digna, água potável, assistência médica e educação tanto a nível local quanto regional. Assim, na realidade, muitos proprietários de cães não podem arcar com medidas preventivas e agirão somente na presença de algum problema grave que ponha em risco a vida de seus animais. Além disso, o Brasil ainda é o lar de uma grande população de cães e

gatos que vivem como “errantes” ou animais de “comunidade”, não só em áreas rurais, mas também nas periferias e em grandes centros urbanos. As autoridades de saúde pública não são capazes de gerir esses animais devido à falta de infraestrutura adequada e pessoal treinado para a realização de um programa de controle efetivo da população em longo prazo. Como resultado, cães e gatos são geralmente expostos a uma vasta gama de parasitos que podem causar doença neles e, eventualmente, nos seus parentes humanos. Esse fato, invariavelmente, nos leva ao conceito de Uma Saúde (*One Health*), que convida veterinários e médicos humanos para unificar seus esforços para combater parasitos zoonóticos (e.g., *T. canis*, *A. caninum*, *D. caninum*, *D. immitis*, *E. granulosus* e *L. infantum*). Na verdade, a unificação das “medicinas veterinária e humana” em uma Única Medicina é fundamental, instrumentalmente para reduzir os riscos de saúde para animais de companhia e seres humanos.

A diversidade notável de ectoparasitos que infestam cães e gatos no Brasil é certamente uma consequência de uma combinação de fatores. A variedade de ambientes naturais e climas encontrados nesse país é impressionante e favorece o estabelecimento de diferentes parasitos em todas as regiões geográficas. Da mesma forma, os cães e gatos que vivem em áreas urbanas ou rurais podem, eventualmente, viver em estreito contato com animais silvestres (e.g., raposas, felinos silvestres e gambás), o que pode, em última instância, favorecer a troca de ectoparasitos como pulgas e carrapatos com esses animais [320-323]. Por exemplo, *R. sanguineus* s.l. foi encontrado em quatis (*Nasua nasua*) em um parque zoológico em uma região metropolitana no nordeste do Brasil [322]. Embora este seja um achado acidental, isso sugere a presença de cães errantes circulando livremente no território do parque e vivendo em contato próximo com animais silvestres mantidos em cativeiro. Por outro lado, cães e gatos intrusos ou que vivem perto de ambientes florestais podem ser encontrados infestados por ectoparasitos associados animais silvestres [12, 28, 50, 321]. A troca de ectoparasitos entre animais domésticos e silvestres também pode ter implicações do ponto de vista da conservação, porque os cães também podem trocar endoparasitos [324-326] e vírus [327328] com espécies selvagens. Por exemplo, vários canídeos silvestres (e.g., *C. thous* e *P. gymnocercus*) e felinos (e.g., *Panthera onca*, *Puma concolor*, *Puma yagouaroundi* e *Leopardus pardalis*) já foram encontrados albergando parasitos que podem afetar cães e gatos, incluindo helmintos (e.g., *A. caninum*, *D. caninum*, *T. canis* e *T. vulpis*) e protozoários (e.g., *L. infantum*) [324-326, 329-333]. Notavelmente, enquanto canídeos silvestres de vida livre infectados com *L. infantum* são geralmente assintomáticos, aqueles mantidos em cativeiro parecem ser mais propensos ao desenvolvimento de sinais clínicos [329] e podem até padecer da infecção [332].

Médicos veterinários que atuam no Brasil são fornecidos com um vasto repertório de produtos com eficácia comprovada contra parasitos de cães e gatos. Na verdade, o registro de produtos veterinários no Brasil envolve várias fases e requisitos regulatórios rigorosos [334]. No entanto, o sucesso de qualquer programa de controle de parasitos depende do uso correto do produto(s) escolhido e da adoção da melhor estratégia para cada situação. Conforme discutido anteriormente, faltam atualmente diretrizes para o diagnóstico, tratamento e controle de parasitos de cães e gatos no Brasil, as quais são urgentemente necessárias. Enquanto isso, na ausência dessas diretrizes, médicos veterinários que atuam no Brasil devem seguir rigorosamente as instruções do fabricante ao utilizar produtos disponíveis no mercado e seguir as orientações internacionais para o controle de parasitos de cães e gatos, eventualmente, adaptando-as às situações regionais. Além disso, o Ministério da Agricultura, Pecuária e Abastecimento (MAPA) estabeleceu recentemente o uso de um passaporte para o trânsito internacional de cães e gatos (http://www.estadao.com.br/noticias/geral,caes-e-gatos-terao-passaporte-e-identificacao-eletronica,1099476,0.htm). Este passaporte será usado como

uma certificação sanitária de origem e incluirá informações sobre o controle de ecto- e endoparasitos. Isso ajudará a reduzir o risco de exportação não intencional de parasitos do Brasil para o exterior.

Ainda existem muitas perguntas pendentes no que concerne os parasitos de cães e gatos no Brasil, algumas das quais já podem ter sido respondidas, no momento em que este trabalho está sendo redigido. Por exemplo, é importante entender por que alguns parasitos são aparentemente restritos às regiões sul e sudeste do Brasil [9]. Esse é o caso de *R. vitalii* e *B. gibsoni*, por exemplo. Enquanto *B. gibsoni* é um parasito relativamente raro em cães no Brasil, *R. vitalii* é bastante comum nos estados do sul e sudeste e pode causar doença grave que pode ser facilmente confundida com babesiose ou erliquiose. Curiosamente, os vetores de ambos os protozoários permanecem desconhecidos, muito embora *Amblyomma aureolatum* seja apontado o vector putativo de *R. vitalii*. A propósito, o papel de *R. sanguineus* s.l. no ciclo natural de *H. canis* no Brasil precisa de uma investigação mais aprofundada. Assim, outras espécies de carrapatos têm sido implicadas como vetores putativos deste protozoário de transmissão oral, incluindo *A. ovale* [335, 336] e *Rhipicephalus microplus* [337]. Alguns parasitos de cães e gatos têm sido raramente relatados no Brasil, provavelmente devido ao limitado esforço de pesquisa sobre esses parasitos pouco estudados, cujo significado médico-veterinário ainda precisa ser determinado. Por exemplo, parasitos enigmáticos como *Cercopithifilaria bainae* [338] foram originalmente descritos no Brasil, mas permaneceram duvidosos e negligenciados por muito tempo. Recentemente, esse nematóide foi redescrito e está emergindo como um filarióide comum de cães em diferentes países europeus [339, 340]. Juntos, esses exemplos indicam que ainda existe muito a ser descoberto em relação aos parasitos de cães e gatos no Brasil.

Mais pesquisas sobre parasitos de cães e gatos no Brasil podem revelar a existência de novas espécies que permaneceram ignoradas ao longo dos anos. De fato, o uso da biologia molecular está revolucionando de forma positiva o nosso conhecimento sobre alguns parasitos “bem conhecidos”, como o carrapato do cão marrom *R. sanguineus* s.l. [52, 161, 162, 341]. Da mesma forma, os dados genéticos sugerem que nematódeos identificados como *A. vasorum* em cães na América do Sul podem representar uma espécie diferente daquela observada em cães europeus [342]. Nessa perspectiva, o uso de técnicas de biologia molecular deve tornar-se uma realidade no cotidiano de médicos veterinários que atuam no Brasil e, portanto, o desenvolvimento de ferramentas moleculares rápidas, economicamente acessíveis deve ser uma prioridade de pesquisa. Por exemplo, os testes sorológicos utilizados atualmente pelas autoridades públicas não são capazes de discriminar cães infectados por *L. infantum* daqueles infectados por *L. braziliensis* ou mesmo distinguir vacinados de cães naturalmente infectados [343]. Portanto, médicos veterinários devem solicitar exames complementares (e.g., isolamento e caracterização do parasito, PCR e seqüenciamento de DNA) para confirmar se o cão está realmente infectado por *L. infantum*, a fim de evitar a eliminação de cães infectados por outros parasitos e para decidir sobre a conduta terapêutica mais adequada a ser adotada. A propósito, é importante também para monitorar a emergência de resistência a certos princípios ativos (e.g., amitraz, fipronil, deltametrina e permetrina), uma vez que esses compostos têm sido muito utilizados para o controle de ectoparasitos no Brasil. Da mesma forma, não há informações sobre a eficácia de alguns endoparasiticidas (e.g, praziquantel, pirantel, febantel e ivermectina), que são amplamente utilizados em diferentes regiões brasileiras. Essas questões devem ser incluídas na agenda de pesquisa de parasitologistas no Brasil, a fim de antecipar os problemas relacionados à resistência parasitária a alguns princípios ativos atualmente utilizados para o controle de ectoparasitos e endoparasitos de cães e gatos no país.

Conclusões

Em conclusão, os benefícios de se ter um cão ou um gato como animal de estimação são indiscutíveis, mas o limite entre benefício e prejuizo é sutil. Certamente, o estreito contato entre animais de estimação e humanos pode involuntariamente representar um risco para humanos. Consequentemente, para evitar riscos potenciais associados a possuir um cão ou um gato de estimação, é fundamental manter esses animais em boa saúde e protegidos contra patógenos zoonóticos. Sem sombra de dúvidas, é nosso dever como veterinários garantir que os pets e seus proprietários possam viver em harmonia sem representar uma ameaça uns aos outros. Mas, sob uma perspectiva holística, veterinários e médicos deveriam trabalhar juntos em busca de melhorar o bem-estar e saúde geral de ambos os animais e humanos. Parafraseando um famoso cantor americano, apenas uma abordagem de “Uma Saúde” pode nos ajudar a “*heal the world*” e “*make it a better place*” para cães, gatos e humanos.

Conflitos de interesse

Os autores declaram que não existem conflitos de interesse.

Agradecimentos

Novartis Animal Health apoiou a preparação dessa revisão. Obrigado a Vinícius Roratto (Figura 1), Thiago André S. de Andrade (Figura 2), Fernando Silva e Pietra Lemos Costa (Figura 4) e Carlos A. P. Parchen (Figura 5) por compartilhar as suas fotos. Obrigado também a Viviana D. Tarallo por preparar os desenhos usados na Figura 3, os quais foram adaptados de livros-texto (e.g., Ref. [13]) e de fotos gentilmente fornecidas pelo Prof. Marcelo de Campos Pereira (http://www.icb.usp.br/~marcelcp/Default.htm).

Contribuições dos autores

FD-T escreveu e DO revisou criticamente o manuscrito. Ambos os autores aprovaram a versão final do manuscrito.

Referências

1. McConnell AR, Brown CM, Shoda TM, Stayton LE, Martin CE: **Friends with benefits: on the positive consequences of pet ownership**. *J Pers Soc Psychol* 2011, **101:**1239–1252.
2. Irwin PJ: **Companion animal parasitology: a clinical perspective**. *Int J Parasitol* 2002, **32:**581–593.
3. Dantas-Torres F, Chomel BB, Otranto D: **Ticks and tick-borne diseases: a One Health perspective**. *Trends Parasitol* 2012, **28:**437–446.
4. Lorusso V, Dantas-Torres F, Caprio F, Manzionna M, Santoro N, Baneth G, Otranto D: **Paediatric visceral leishmaniasis in Italy: a 'One Health' approach is needed**. *Parasit Vectors* 2013, **6:**123.
5. Pullan R, Brooker S: **The health impact of polyparasitism in humans: are we under-estimating the burden of parasitic diseases?** *Parasitology* 2008, **135:**783–794.

6. den Boer M, Argaw D, Jannin J, Alvar J: **Leishmaniasis impact and treatment access**. *Clin Microbiol Infect* 2011, **17:**1471–1477.
7. Lustigman S, Prichard RK, Gazzinelli A, Grant WN, Boatin BA, McCarthy JS, Basáñez MG: **A research agenda for helminth diseases of humans: the problem of helminthiases**. *PLoS Negl Trop Dis* 2012, **6:**e1582.
8. Ribeiro VM: **Controle de helmintos de cães e gatos**. *Rev Bras Parasitol Vet* 2004, **13**(Suppl 1):88–95.
9. Dantas-Torres F: **Canine vector-borne diseases in Brazil**. *Parasit Vectors* 2008, **1:**25.
10. Reck J, Soares JF, Termignoni C, Labruna MB, Martins JR: **Tick toxicosis in a dog bitten by *Ornithodoros brasiliensis***. *Vet Clin Pathol* 2011, **40:**356–360.
11. Evans DE, Martins JR, Guglielmone AA: **A review of the ticks (Acari, ixodida) of Brazil, their hosts and geographic distribution - 1. The state of Rio Grande do Sul, southern Brazil**. *Mem Inst Oswaldo Cruz* 2000, **95:**453–470.
12. Labruna MB, Homem VS, Heinemann MB, Ferreira Neto JS: **Ticks (Acari: Ixodidae) associated with rural dogs in Uruará, eastern Amazon, Brazil**. *J Med Entomol* 2000, **37:**774–776.
13. Linardi PM, Guimarães LR: *Sifonápteros do Brasil*. São Paulo: Ed. Museu de Zoologia USP/FAPESP; 2000.
14. Guimarães JH, Tucci EC, Barros-Battesti DM: *Ectoparasitos de importância veterinária*. São Paulo: Plêiade/FAPESP; 2001.
15. Rodrigues AFSF, Daemon E, D'Agosto M: **Investigação sobre alguns ectoparasitos em cães de rua no município de Juiz de Fora, Minas Gerais**. *Rev Bras Parasitol Vet* 2001, **10**:13–19.
16. Szabó MP, Cunha TM, Pinter A, Vicentini F: **Ticks (Acari: Ixodidae) associated with domestic dogs in Franca region, São Paulo, Brazil**. *Exp Appl Acarol* 2001, **25:**909–916.
17. Bellato V, Sartor AA, Souza AP, Ramos BC: **Ectoparasites in dogs from Lages municipality, Santa Catarina, Brazil**. *Rev Bras Parasitol Vet* 2003, 12:95–98.
18. Dantas-Torres F, Figueredo LA, Faustino MAG: **Ectoparasites of dogs from some municipalities of the metropolitan region of Recife, Pernambuco state, Brazil**. *Rev Bras Parasitol Vet* 2004, **13:**151–154.
19. Heukelbach J, Costa AM, Wilcke T, Mencke N, Feldmeier H: **The animal reservoir of *Tunga penetrans* in severely affected communities of north-east Brazil**. *Med Vet Entomol* 2004, **18:**329–335.
20. Souza CP, Scott FB, Pereira MJS: **Validity and reprodutibility of otoscopy and pinnal pedal reflex on the diagnostic of *Otodectes cynotis* infestation in dogs**. *Rev Bras Parasitol Vet* 2004, **13:**111–114.
21. Scofield A, Riera MDF, Elisei C, Massard CL, Linardi PM: **Occurrence of *Rhopalopsyllus lutzi lutzi* (Baker) (Siphonaptera, Rhopalopsyllidae) on *Canis familiaris* (Linnaeus) from rural areas of the county of Piraí, State of Rio de Janeiro, Brazil.** *Rev Bras Entomol* 2005, **49:**159–161.
22. Castro MCM, Rafael JA: **Ectoparasites on cats and dogs from Manaus, Amazonas State, Brazil**. *Acta Amaz* 2006, **36:**535–538.
23. Soares AO, Souza AD, Feliciano EA, Rodrigues AF, D'Agosto M, Daemon E: **Evaluation of ectoparasites and hemoparasites in dogs kept in apartments and houses with yards in the city of Juiz de Fora, Minas Gerais, Brazil**. *Rev Bras Parasitol Vet* 2006, **15:**13–16.

24. Dantas-Torres F, Figueredo LA: ***Heterodoxus spiniger* (Enderlein, 1909) on domestic dogs (*Canis familiaris*, L. 1758) from the city of Recife, Pernambuco state, Brazil**. *Braz J Vet Res Anim Sci* 2007, **43:**77–80.
25. Szabó MP, Olegário MM, Santos AL: **Tick fauna from two locations in the Brazilian savannah**. *Exp Appl Acarol* 2007, **43:**73–84.
26. Cardoso CP, Stalliviere FM, Schelbauer CA, de Souza AP, Bellato V, Sartor AA: ***Amblyomma tigrinum* in the municipality of Lages, SC, and biology observations under laboratory conditions**. *Rev Bras Parasitol Vet* 2008, **17:**56–58.
27. Rocha GS, Ahid SMM, Bezerra ACDS, Filgueira KD, Santos JPS: **Mites frequency in dogs and cats at the city of Mossoro, Rio Grande do Norte, Brazil**. *Acta Sci Vet* 2008, **36:**263–266.
28. Rodrigues DF, Daemon E, Rodrigues AF. **Characterization of ectoparasites on dogs in the nucleus of urban expansion of Juiz de Fora, Minas Gerais, Brazil**. *Rev Bras Parasitol Vet* 2008, **17:**185–188.
29. Dantas-Torres F: **Ticks on domestic animals in Pernambuco, Northeastern Brazil**. *Rev Bras Parasitol Vet* 2009, **18:**22–28.
30. Dantas-Torres F, Melo MF, Figueredo LA, Brandão-Filho SP: **Ectoparasite infestation on rural dogs in the municipality of São Vicente Férrer, Pernambuco, Northeastern Brazil**. *Rev Bras Parasitol Vet* 2009, **18:**75–77.
31. Martins TF, Spolidorio MG, Batista TC, Oliveira IA, Yoshinari NH, Labruna MB: **Occurrence of ticks (Acari: Ixodidae) in the municipality of Goiatins, Tocantins**. *Rev Bras Parasitol Vet* 2009, **18:**50–52.
32. Klimpel S, Heukelbach J, Pothmann D, Rückert S: **Gastrointestinal and ectoparasites from urban stray dogs in Fortaleza (Brazil): high infection risk for humans?** *Parasitol Res* 2010, **107:**713–719.
33. Szabó MP, de Souza LG, Olegário MM, Ferreira FA, de Albuquerque Pajuaba Neto A: **Ticks (Acari: Ixodidae) on dogs from Uberlândia, Minas Gerais, Brazil**. *Transbound Emerg Dis* 2010 **57:**72–74.
34. Guimarães AM, Lima BS, Rocha CMBM: **Parasitic ectofauna of urban domiciled dogs examined in private veterinary clinics from Lavras municipality, Minas Gerais state, Brazil**. *Cien Anim Bras* 2011, **12:**172–177.
35. Costa-Junior LM, Rembeck K, Mendonça FL, Azevedo SC, Passos LM, Ribeiro MF: **Occurrence of ectoparasites on dogs in rural regions of the state of Minas Gerais, Brazil.** *Rev Bras Parasitol Vet* 2012, **21:**237–242.
36. Heukelbach J, Frank R, Ariza L, de Sousa Lopes I, de Assis E Silva A, Borges AC, Limongi JE, de Alencar CH, Klimpel S: **High prevalence of intestinal infections and ectoparasites in dogs, Minas Gerais State (southeast Brazil)**. *Parasitol Res* 2012, **111:**1913–1921.
37. Mazioli R, Szabó M, Mafra C: ***Amblyomma nodosum* (Acari: Ixodidae) parasitizing a domestic dog in Colatina, Espírito Santo, Brazil**. *Rev Bras Parasitol Vet* 2012, **21**:428–432.
38. Santos JL, Magalhães NB, Dos Santos HA, Ribeiro RR, Guimarães MP: **Parasites of domestic and wild canids in the region of Serra do Cipó National Park, Brazil**. *Rev Bras Parasitol Vet* 2012, **21:**270–277.
39. Costa AP, Silva AB, Costa FB, Xavier GS, Martins TF, Labruna MB, Guerra RM: **A survey of ectoparasites infesting urban and rural dogs of Maranhão state, Brazil**. *J Med Entomol* 2013, **50:**674–678.
40. Reck J, Marks FS, Guimarães JA, Termignoni C, Martins JR: **Epidemiology of *Ornithodoros brasiliensis* (mouro tick) in the southern Brazilian highlands and**

the description of human and animal retrospective cases of tick parasitism. *Ticks Tick Borne Dis* 2013, **4:**101–109.

41. Souza CP, Verocai GG, Balbi M, Scott FB: **Video otoscopy as a diagnostic tool for canine otoacariasis**. *Rev Bras Parasitol Vet* 2013, **22:**440–442.
42. Mendes-de-Almeida F, Faria MC, Branco AS, Serrão ML, Souza AM, Almosny N, Charme M, Labarthe N: **Sanitary conditions of a colony of urban feral cats (*Felis catus* Linnaeus, 1758) in a zoological garden of Rio de Janeiro, Brazil**. *Rev Inst Med Trop Sao Paulo* 2004, **46:**269–274.
43. Pereira SA, Schubach TMP, Figueiredo FB, Paes Leme LR, Santos IB, Okamoto T, Cuzzi T, Reis RS, Schubach A: **Demodicosis associated with sporotrichosis and pediculosis in a positive FIV/FeLV cat**. *Acta Sci Vet* 2005, **33:**75–78.
44. Mendes-de-Almeida F, Labarthe N, Guerrero J, Faria MC, Branco AS, Pereira CD, Barreira JD, Pereira MJ: **Follow-up of the health conditions of an urban colony of free-roaming cats (*Felis catus* Linnaeus, 1758) in the city of Rio de Janeiro, Brazil**. *Vet Parasitol* 2007, **147:**9–15.
45. Romeiro ET, Alves LC, Soares YM, Matoso UN, Faustino MA: **Infestation by *Lynxacarus radovskyi* (Tenorio, 1974) in domestic cats from Metropolitan Region of Recife, Pernambuco, Brazil**. *Rev Bras Parasitol Vet* 2007, **16:**159–162.
46. Aguiar J, Machado MLS, Ferreira RR, Hünning PS, Muschner AC, Ramos RZ: **Mixed infestation by *Lynxacarus radovskyi* and *Felicola subrostratus* in a cat in Porto Alegre, RS, Brazil**. *Acta Scie Vet* 2009, **37:**301–305.
47. Stalliviere FM, Bellato V, Souza AP, Sartor AA, Moura AB, Rosa LD: **Ectoparasites and intestinal helminths in *Felis catus domesticus* from Lages city, SC, Brazil and social-economical and cultural aspects of owners of family pets**. *Rev Bras Parasitol Vet* 2009, **18:**26–31.
48. Ferreira DRA, Alves LC, Faustino MAG: **Ectoparasitic species from *Felis catus domesticus* (Linnaeus, 1758) in João Pessoa city, Paraíba state, Brazil**. *Biotemas* 2010, **23:**43–50.
49. Mendes-de-Almeida F, Crissiuma AL, Gershony LC, Willi LM, Paiva JP, Guerrero J, Labarthe N: **Characterization of ectoparasites in an urban cat (*Felis catus* Linnaeus, 1758) population of Rio de Janeiro, Brazil**. *Parasitol Res* 2011, **108:**1431–1435.
50. Silva AS, Silva MK, Monteiro SG: **Parasitism by *Amblyomma triste* in domestic cat.** *Rev Bras Parasitol Vet* 2007, **16:**108–109.
51. Linardi PM, Santos JL: ***Ctenocephalides felis felis* vs. *Ctenocephalides canis* (Siphonaptera: Pulicidae): some issues in correctly identify these species**. *Rev Bras Parasitol Vet* 2012, **21:**345–354.
52. Dantas-Torres F, Latrofa MS, Annoscia G, Giannelli A, Parisi A, Otranto D: **Morphological and genetic diversity of *Rhipicephalus sanguineus* sensu lato from the New and Old Worlds**. *Parasit Vectors* 2013, **6:**213.
53. Barros-Battesti DM, Arzua M, Bechara GH. *Carrapatos de importância médico-veterinária da Região Neotropical: Um guia ilustrado para identificação de espécies*. São Paulo: Vox/ International Consortium on Ticks and Tick-borne Diseases (ICTTD-3)/Butantan; 2006.
54. Aragão HB, Fonseca F: **Notas de ixodologia. VIII. Lista e chave para os representantes da fauna ixodológica brasileira.** *Mem Inst Oswaldo Cruz* 1961, **59:**115–129.
55. Labruna MB, Campos Pereira M: **Carrapato em cães no Brasil**. *Clin Vet* 2001, **30:** 24–32.

56. Martins TF, Onofrio VC, Barros-Battesti DM, Labruna MB: **Nymphs of the genus *Amblyomma* (Acari: Ixodidae) of Brazil: descriptions, redescriptions, and identification key**. *Ticks Tick Borne Dis* 2010, **1:**75–99.
57. Azeredo-Espin AM, Madeira NG: **Primary myiasis in dog caused by *Phaenicia eximia* (Diptera: Calliphoridae) and preliminary mitochondrial DNA analysis of the species in Brazil**. *J Med Entomol* 1996, **33:**839–843.
58. Silva Junior VP, Souza Leandro A, Moya Borja GE**: Occurrence of berne, *Dermatobia hominis* (Diptera: Cuterebridae) in several host, in the Rio de Janeiro, Brazil**. *Parasitol día* 1998, **22:**97–101.
59. Cramer-Ribeiro BC, Sanavria A, Oliveira MQ, Souza FS, Rocco FS, Cardoso PG: **Inquiry of cases of myiasis by *Cochliomyia hominivorax* in dogs of the southern zone of Rio de Janeiro city in 2000**. *Braz J Vet Res Anim Sci* 2002, **39:**171–175.
60. Cramer-Ribeiro BC, Sanavria A, Oliveira MQ, Souza FS, Rocco FS, Cardoso PG: **Inquiry of cases of myiasis by *Dermatobia hominis* in dogs of the southern zone of Rio de Janeiro municipality in 2000**. *Braz J Vet Res Anim Sci* 2002, **39:**176–180.
61. Cramer-Ribeiro BC, Sanavria A, Monteiro HHMS, Oliveira MQ, Souza FS: **Inquiry of cases of myiasis by *Cochliomyia hominivorax* in dogs (*Canis familiaris*) of the Northern and Western zones of Rio de Janeiro city in 2000**. *Braz J Vet Res Anim Sci* 2003, **40:**13–20.
62. Cardozo SV, Ramadinha RR: **Evaluation of myiasis treatment in dogs using nitenpyram**. *R Bras Ci Vet* 2007, **14:**139–142.
63. Correia TR, Scott FB, Verocai GG, Souza CP, Fernandes JI, Melo RM, Vieira VP, Ribeiro FA: **Larvicidal efficacy of nitenpyram on the treatment of myiasis caused by *Cochliomyia hominivorax* (Diptera: Calliphoridae) in dogs**. *Vet Parasitol* 2010, **173:**169–172.
64. Marotta CR, Scherer PO, Sanavria A: **Cutaneous and oro-nasal internal myiasis by *Cochliomyia hominivorax* (Coquerel, 1858) in feline (*Felis catus*) - Case report**. *Rev Bras Med Vet* 2011, **33:**137-141.
65. Cansi ER, Demo C: **Occurrence of myiasis in pets from the Federal District, in Brazil**. *Acta Sci Vet* 2011, **39:**982.
66. Barros ATM, Koller WW, Catto JB, Soares CO: ***Stomoxys calcitrans* outbreaks in pastured beef cattle in the State of Mato Grosso do Sul, Brazil**. *Pesq Vet Bras* 2010, **30:**945–952.
67. Freitas MG, Costa HMA: **Lista de helmintos parasitos dos animais domésticos do Brasil**. *Arq Esc Vet UFMG* 1959, **12:**443-510.
68. Travassos L, Freitas JFT, Kohn A: **Trematódeos do Brasil**. *Mem Inst Oswaldo Cruz* 1959, **67**:1–886.
69. Vicente JJ, Rodrigues HO, Gomes DC, Pinto RM: **Nematóides do Brasil. Parte V. Nematóides de mamíferos**. *Rev Bras Zool* 1997, **14**(Supl 1):1–452.
70. Costa JO, Guimarães MP, Lima WS, Lima EA: **Endo and ectoparasites of dogs from Vitoria county - Espírito Santo – Brazil**. *Arq Bras Med Vet Zootec* 1990, **42:**451–452.
71. Gennari SM, Kasai N, Pena HFJ, Cortez A: **Occurrence of protozoa and helminthes in faecal samples of dogs and cats from São Paulo city**. *Braz J Vet Res An Sci* 1999, **36:**87–91.
72. Hoffmann AN, Malgor R, de la Rue ML: **Prevalence of *Echinococcus granulosus* (Batsch, 1786) in urban stray dogs from Dom Pedrito in the state of Rio Grande do Sul, Brazil**. *Cienc Rural* 2001, **31:**843–847.
73. Gontijo CM, da Silva ES, de Fuccio MB, de Sousa MC, Pacheco RS, Dias ES, Andrade Filho JD, Brazil RP, Melo MN: **Epidemiological studies of an outbreak of**

cutaneous leishmaniasis in the Rio Jequitinhonha Valley, Minas Gerais, Brazil. *Acta Trop* 2002, **81:**143–150.
74. Oliveira-Sequeira TC, Amarante AF, Ferrari TB, Nunes LC: **Prevalence of intestinal parasites in dogs from São Paulo State, Brazil**. *Vet Parasitol* 2002, **103:**19–27.
75. Oliveira-Júnior SD, Barçante JM, Barçante TA, Ribeiro VM, Lima WS: **Ectopic location of adult worms and first-stage larvae of *Angiostrongylus vasorum* in an infected dog**. *Vet Parasitol* 2004, **121**:293–296.
76. Sreekumar C, Hill DE, Miska KB, Rosenthal BM, Vianna MC, Venturini L, Basso W, Gennari SM, Lindsay DS, Dubey JP: ***Hammondia heydorni*: evidence of genetic diversity among isolates from dogs**. *Exp Parasitol* 2004, **107:**65–71.
77. Blazius RD, Emerick S, Prophiro JS, Romão PR, Silva OS: **Occurrence of protozoa and helminthes in faecal samples of stray dogs from Itapema City, Santa Catarina**. *Rev Soc Bras Med Trop* 2005, **38:**73–74.
78. Herrera HM, Norek A, Freitas TP, Rademaker V, Fernandes O, Jansen AM: **Domestic and wild mammals infection by *Trypanosoma evansi* in a pristine area of the Brazilian Pantanal region**. *Parasitol Res* 2005, **96:**121–126.
79. Passos LM, Geiger SM, Ribeiro MF, Pfister K, Zahler-Rinder M: **First molecular detection of *Babesia vogeli* in dogs from Brazil**. *Vet Parasitol* 2005, **127:**81–85.
80. Rubini AS, dos Santos Paduan K, Cavalcante GG, Ribolla PE, O'Dwyer LH: **Molecular identification and characterization of canine *Hepatozoon* species from Brazil**. *Parasitol Res* 2005, **97:**91–93.
81. Savani ES, Nunes VL, Galati EA, Castilho TM, Araujo FS, Ilha IM, Camargo MC, D'Auria SR, Floeter-Winter LM: **Occurrence of co-infection by *Leishmania* (*Leishmania*) *chagasi* and *Trypanosoma* (*Trypanozoon*) *evansi* in a dog in the state of Mato Grosso do Sul, Brazil**. *Mem Inst Oswaldo Cruz* 2005, **100:**739–741.
82. Andrade HM, Reis AB, dos Santos SL, Volpini AC, Marques MJ, Romanha AJ: **Use of PCR-RFLP to identify *Leishmania* species in naturally-infected dogs**. *Vet Parasitol* 2006, **140:**231–238.
83. e Silva LM, Miranda RR, Santos HA, Rabelo EM: **Differential diagnosis of dog hookworms based on PCR-RFLP from the ITS region of their rDNA**. *Vet Parasitol* 2006, **140:**373–377.
84. Labruna MB, Pena HFJ, Souza SLP, Pinter A, Silva JCR, Ragozo AMA, Camargo LMA, Gennari SM: **Prevalence of endoparasites in dogs from the urban area of Monte Negro municipality, Rondônia, Brazil**. *Arq Inst Biol São Paulo* 2006, **73:**183–193.
85. Madeira MF, Schubach A, Schubach TM, Pacheco RS, Oliveira FS, Pereira SA, Figueiredo FB, Baptista C, Marzochi MC: **Mixed infection with *Leishmania* (*Viannia*) *braziliensis* and *Leishmania* (*Leishmania*) *chagasi* in a naturally infected dog from Rio de Janeiro, Brazil**. *Trans R Soc Trop Med Hyg* 2006, **100:**442–445.
86. Pereira BJ, Girardelli GL, Trivilin LO, Lima VR, Nunes Lde C, Martins IV: **The occurrence of dioctophymosis in dogs from Municipality of Cachoeiro do Itapemirim in the State of Espírito Santo, Brazil, from May to December of 2004**. *Rev Bras Parasitol Vet* 2006, **15:**123–125.
87. Trapp SM, Messick JB, Vidotto O, Jojima FS, de Morais HS: ***Babesia gibsoni* genotype Asia in dogs from Brazil**. *Vet Parasitol* 2006, **141:**177–180.
88. Velasquez LG, Membrive N, Membrive U, Rodrigues G, Reis N, Lonardoni MV, Teodoro U, Tessmann IP, Silveira TG: **PCR in the investigation of canine American tegumentary leishmaniasis in northwestern Paraná State, Brazil**. *Cad Saude Publica* 2006, **22:**571–578.

89. Forlano MD, Teixeira KR, Scofield A, Elisei C, Yotoko KS, Fernandes KR, Linhares GF, Ewing SA, Massard CL: **Molecular characterization of *Hepatozoon* sp. from Brazilian dogs and its phylogenetic relationship with other *Hepatozoon* spp**. *Vet Parasitol* 2007, **145:**21–30.
90. Gomes AH, Ferreira IM, Lima ML, Cunha EA, Garcia AS, Araújo MF, Pereira-Chioccola VL: **PCR identification of *Leishmania* in diagnosis and control of canine leishmaniasis.** *Vet Parasitol* 2007, **144:**234–241.
91. Katagiri S, Oliveira-Sequeira TCG: **Zoonoses caused by dog intestinal parasites and the problem of diagnosis**. *Arq Inst Biol* 2007, **74**:175–184.
92. Mundim MJ, Rosa LA, Hortêncio SM, Faria ES, Rodrigues RM, Cury MC: **Prevalence of *Giardia duodenalis* and *Cryptosporidium* spp. in dogs from different living conditions in Uberlândia, Brazil**. *Vet Parasitol* 2007, **144:**356–359.
93. Nakagawa TL, Bracarense AP, dos Reis AC, Yamamura MH, Headley SA: **Giant kidney worm (*Dioctophyma renale*) infections in dogs from Northern Paraná, Brazil**. *Vet Parasitol* 2007, **145:**366–370.
94. Souza SL, Gennari SM, Richtzenhain LJ, Pena HF, Funada MR, Cortez A, Gregori F, Soares RM: **Molecular identification of *Giardia duodenalis* isolates from humans, dogs, cats and cattle from the state of São Paulo, Brazil, by sequence analysis of fragments of glutamate dehydrogenase (gdh) coding gene**. *Vet Parasitol* 2007, **149:**258–264.
95. Thomaz A, Meireles MV, Soares RM, Pena HF, Gennari SM: **Molecular identification of *Cryptosporidium* spp. from fecal samples of felines, canines and bovines in the state of São Paulo, Brazil**. *Vet Parasitol* 2007, **150:**291–296.
96. Tolezano JE, Uliana SR, Taniguchi HH, Araújo MF, Barbosa JA, Barbosa JE, Floeter-Winter LM, Shaw JJ: **The first records of *Leishmania* (*Leishmania*) *amazonensis* in dogs (*Canis familiaris*) diagnosed clinically as having canine visceral leishmaniasis from Araçatuba County, São Paulo State, Brazil**. *Vet Parasitol* 2007, **149:**280–284.
97. Volotão AC, Costa-Macedo LM, Haddad FS, Brandão A, Peralta JM, Fernandes O: **Genotyping of *Giardia duodenalis* from human and animal samples from Brazil using beta-giardin gene: a phylogenetic analysis**. *Acta Trop* 2007, **102:**10–19.
98. Campos Filho PC, Barros LM, Campos JO, Braga VB, Cazorla IM, Albuquerque GR, Carvalho SM: **Zoonotic parasites in dog feces at public squares in the municipality of Itabuna, Bahia, Brazil**. *Rev Bras Parasitol Vet* 2008, **17:**206–209.
99. Fernandes AB, Baêta BA, Filho WF, Massad FV, Rebouças FA, De Carvalho JB, Lopes CW: **Relationship between companion animals and intestinal parasites in children at municipality of Seropédica, RJ**. *Rev Bras Parasitol Vet* 2008, **17**(Suppl 1):296–300.
100. Katagiri S, Oliveira-Sequeira TC: **Prevalence of dog intestinal parasites and risk perception of zoonotic infection by dog owners in São Paulo State, Brazil**. *Zoonoses Public Health* 2008, **55:**406–413.
101. Meireles P, Montiani-Ferreira F, Thomaz-Soccol V: **Survey of giardiosis in household and shelter dogs from metropolitan areas of Curitiba, Paraná state, Southern Brazil**. *Vet Parasitol* 2008, **152:**242–248.
102. Oliveira Lima AN, da Silva Santos S, Herrera HM, Gama C, Cupolillo E, Jansen AM, Fernandes O: ***Trypanosoma evansi*: molecular homogeneity as inferred by phenetical analysis of ribosomal internal transcribed spacers DNA of an eclectic parasite**. *Exp Parasitol* 2008, **118:**402–407.

103. Rubini AS, dos Santos Paduan K, Von Ah Lopes V, O'Dwyer LH: **Molecular and parasitological survey of *Hepatozoon canis* (Apicomplexa: Hepatozoidae) in dogs from rural area of São Paulo state, Brazil**. *Parasitol Res* 2008, **102:**895–899.
104. Shigueru FJ, Garcia JL, Vidotto MC, Balarin MR, Fabretti AK, Gasparini MR, Coelho AL, Vidotto O: **Occurrence and molecular characterization of *Babesia* species in a canine hospital population in the Londrina Region, Paraná State, Brazil**. *Rev Bras Parasitol Vet* 2008, **17**(Suppl 1):277–283.
105. Torrico KJ, Santos KR, Martins T, Paz e Silva FM, Takahira RK, Lopes RS: **Occurrence of gastrointestinal parasites in dogs and cats in the laboratory of routine of parasitic diseases FMVZ/Unesp-Botucatu, SP**. *Rev Bras Parasitol Vet* 2008, **17**(Suppl 1):182–183.
106. Costa-Júnior LM, Ribeiro MF, Rembeck K, Rabelo EM, Zahler-Rinder M, Hirzmann J, Pfister K, Passos LM: **Canine babesiosis caused by *Babesia canis vogeli* in rural areas of the State of Minas Gerais, Brazil and factors associated with its seroprevalence**. *Res Vet Sci* 2009, **86:**257–260.
107. Furtado AP, Do Carmo ES, Giese EG, Vallinoto AC, Lanfredi RM, Santos JN: **Detection of dog filariasis in Marajo Island, Brazil by classical and molecular methods**. *Parasitol Res* 2009, **105:**1509–1515
108. Madeira MF, Sousa MA, Barros JH, Figueiredo FB, Fagundes A, Schubach A, DE Paula CC, Faissal BN, Fonseca TS, Thoma HK, Marzochi MC: ***Trypanosoma caninum* n. sp. (Protozoa: Kinetoplastida) isolated from intact skin of a domestic dog (*Canis familiaris*) captured in Rio de Janeiro, Brazil**. *Parasitology* 2009, **136:**411–423.
109. O'Dwyer LH, Lopes VV, Rubini AS, Paduan Kdos S, Ribolla PE: ***Babesia* spp. infection in dogs from rural areas of São Paulo State, Brazil**. *Rev Bras Parasitol Vet* 2009, **18:**23–26.
110. Oliveira LP, Cardozo GP, Santos EV, Mansur MA, Donini IA, Zissou VG, Roberto PG, Marins M: **Molecular analysis of the rRNA genes of *Babesia* spp and *Ehrlichia canis* detected in dogs from Ribeirão Preto, Brazil**. *Braz J Microbiol* 2009, **40:**238–240.
111. Quaresma PF, Murta SM, Ferreira Ede C, Rocha-Lima AC, Xavier AA, Gontijo CM: **Molecular diagnosis of canine visceral leishmaniasis: identification of *Leishmania* species by PCR-RFLP and quantification of parasite DNA by real-time PCR.** *Acta Trop* 2009, **111:**289–294.
112. Spolidorio MG, Labruna MB, Zago AM, Donatele DM, Caliari KM, Yoshinari NH: ***Hepatozoon canis* infecting dogs in the State of Espírito Santo, southeastern Brazil**. *Vet Parasitol* 2009, **163:**357–361.
113. Dantas-Torres F, de Paiva-Cavalcanti M, Figueredo LA, Melo MF, Silva FJ, Silva AL, Almeida EL, Brandão-Filho SP: **Cutaneous and visceral leishmaniasis in dogs from a rural community in northeastern Brazil**. *Vet Parasitol* 2010, **170:**313–317.
114. Gomes PV, Mundim MJ, Mundim AV, de Ávila DF, Guimarães EC, Cury MC: **Occurrence of *Hepatozoon* sp. in dogs in the urban area originating from a municipality in southeastern Brazil**. *Vet Parasitol* 2010, **174:**155–161.
115. Sevá AP, Funada MR, Souza SO, Nava A, Richtzenhain LJ, Soares RM: **Occurrence and molecular characterization of *Cryptosporidium* spp. isolated from domestic animals in a rural area surrounding Atlantic dry forest fragments in Teodoro Sampaio municipality, State of São Paulo, Brazil**. *Rev Bras Parasitol Vet* 2010, **19:**249–253.

116. Coelho WM, Amarante AF, Apolinário Jde C, Coelho NM, Bresciani KD: **Occurrence of *Ancylostoma* in dogs, cats and public places from Andradina city, São Paulo state, Brazil**. *Rev Inst Med Trop Sao Paulo* 2011, **53:**181–184.
117. Dias ES, Regina-Silva S, França-Silva JC, Paz GF, Michalsky EM, Araújo SC, Valadão JL, de Oliveira Lara-Silva F, de Oliveira FS, Pacheco RS, Fortes-Dias CL: **Eco-epidemiology of visceral leishmaniasis in the urban area of Paracatu, state of Minas Gerais, Brazil**. *Vet Parasitol* 2011, **176:**101–111.
118. Duarte SC, Parente JA, Pereira M, Soares CM, Linhares GF: **Phylogenetic characterization of *Babesia canis vogeli* in dogs in the state of Goiás, Brazil**. *Rev Bras Parasitol Vet* 2011, **20:**274–280.
119. Soares JF, Girotto A, Brandão PE, Da Silva AS, França RT, Lopes ST, Labruna MB: **Detection and molecular characterization of a canine piroplasm from Brazil**. *Vet Parasitol* 2011, **180:**203–208.
120. Costa LM Jr, Zahler-Rinder M, Ribeiro MF, Rembeck K, Rabelo EM, Pfister K, Passos LM: **Use of a Real Time PCR for detecting subspecies of *Babesia canis***. *Vet Parasitol* 2012, **188:**160–163.
121. Eloy LJ, Lucheis SB: **Hemoculture and polymerase chain reaction using primers TCZ1/TCZ2 for the diagnosis of canine and feline trypanosomiasis**. *ISRN Vet Sci* 2012, **2012:**419378.
122. Gonçalves AQ, Ascaso C, Santos I, Serra PT, Julião GR, Orlandi PP: ***Calodium hepaticum*: household clustering transmission and the finding of a source of human spurious infection in a community of the Amazon region**. *PLoS Negl Trop Dis* 2012, **6:**e1943.
123. Langoni H, Matteucci G, Medici B, Camossi LG, Richini-Pereira VB, Silva RC: **Detection and molecular analysis of *Toxoplasma gondii* and *Neospora caninum* from dogs with neurological disorders**. *Rev Soc Bras Med Trop* 2012, **45:**365–368.
124. Lemos TD, Cerqueira Ade M, Toma HK, Silva AV, Corrêa RG, Paludo GR, Massard CL, Almosny NR: **Detection and molecular characterization of piroplasms species from naturally infected dogs in southeast Brazil**. *Rev Bras Parasitol Vet* 2012, **21**:137–142.
125. Martins CM, Barros CC, Bier D, Marinho AP, Figueiredo JM, Hoffmann JL, Molento MB, Biondo AW: **Dog parasite incidence and risk factors, from sampling after one-year interval, in Pinhais, Brazil**. *Rev Bras Parasitol Vet* 2012, **21:**101–106.
126. Paz e Silva FM, Monobe MM, Lopes RS, Araujo JP Jr: **Molecular characterization of *Giardia duodenalis* in dogs from Brazil**. *Parasitol Res* 2012, **110:**325–334.
127. Santana VL, Souza AP, Lima DASD, Araújo AL, Justiniano SV, Dantas RP, Guedes PMM, Melo MA: **Clinical and laboratorial characterization of naturally infected *Trypanosoma cruzi* dogs in the northeastern semi-arid**. *Pesq Vet Bras* 2012, **32:**536–541.
128. Leça Júnior NF, Dos Anjos Almeida V, Santos Carvalho F, Rego Albuquerque G, Lessa Silva F: **First report of *Trypanosoma cruzi* infection in naturally infected dogs from southern Bahia, Brazil**. *Rev Bras Parasitol Vet,* in press.
129. Morais RC, Gonçalves SC, Costa PL, da Silva KG, da Silva FJ, Silva RP, de Brito ME, Brandão-Filho SP, Dantas-Torres F, de Paiva-Cavalcanti M: **Detection of *Leishmania infantum* in animals and their ectoparasites by conventional PCR and real time PCR**. *Exp Appl Acarol* 2013, **59:**473–481.
130. Rocha FL, Roque AL, Arrais RC, Santos JP, Lima VS, Xavier SC, Cordeir-Estrela P, D'Andrea PS, Jansen AM: ***Trypanosoma cruzi* TcI and TcII transmission among

wild carnivores, small mammals and dogs in a conservation unit and surrounding areas, Brazil. *Parasitology* 2013, **140:**160–170.
131. Labarthe N, Ferreira AM, Guerrero J, Newcomb K, Paes-de-Almeida E: **Survey of *Dirofilaria immitis* (Leidy, 1856) in random source cats in metropolitan Rio de Janeiro, Brazil, with descriptions of lesions**. *Vet Parasitol* 1997, **71:**301–306.
132. Serra CM, Uchôa CM, Coimbra RA: **Parasitological study with faecal samples of stray and domiciliated cats (*Felis catus domesticus*) from the Metropolitan Area of Rio de Janeiro, Brazil**. *Rev Soc Bras Med Trop* 2003, **36:**331–334.
133. Labarthe N, Serrão ML, Ferreira AM, Almeida NK, Guerrero J: **A survey of gastrointestinal helminths in cats of the metropolitan region of Rio de Janeiro, Brazil**. *Vet Parasitol* 2004, **123:**133–139.
134. Coelho WM, do Amarante AF, de Soutello RV, Meireles MV, Bresciani KD: **Occurrence of gastrointestinal parasites in fecal samples of cats in Andradina City, São Paulo**. *Rev Bras Parasitol Vet* 2009, **18:**46–49.
135. Verocai GG, Measures LN, Azevedo FD, Correia TR, Fernandes JI, Scott FB: ***Dioctophyme renale* (Goeze, 1782) in the abdominal cavity of a domestic cat from Brazil**. *Vet Parasitol* 2009, **161:**342–344.
136. Sobrinho LS, Rossi CN, Vides JP, Braga ET, Gomes AA, de Lima VM, Perri SH, Generoso D, Langoni H, Leutenegger C, Biondo AW, Laurenti MD, Marcondes M: **Coinfection of *Leishmania chagasi* with *Toxoplasma gondii*, Feline Immunodeficiency Virus (FIV) and Feline Leukemia Virus (FeLV) in cats from an endemic area of zoonotic visceral leishmaniasis**. *Vet Parasitol* 2012, **187:**302–306.
137. Ramos DG, Scheremeta RG, Oliveira AC, Sinkoc AL, Pacheco R de C: **Survey of helminth parasites of cats from the metropolitan area of Cuiabá, Mato Grosso, Brazil.** *Rev Bras Parasitol Vet* 2013, **22:**201–206.
138. Perez RR, Rubini AS, O'Dwyer LH: **The first report of *Hepatozoon* spp. (Apicomplexa, Hepatozoidae) in domestic cats from São Paulo state, Brazil**. *Parasitol Res* 2004, **94:**83–85.
139. Savani ES, de Oliveira Camargo MC, de Carvalho MR, Zampieri RA, dos Santos MG, D'Auria SR, Shaw JJ, Floeter-Winter LM: **The first record in the Americas of an autochthonous case of *Leishmania* (*Leishmania*) *infantum chagasi* in a domestic cat (*Felix catus*) from Cotia County, São Paulo State, Brazil**. *Vet Parasitol* 2004, **120:**229–233.
140. Schubach TM, Figueiredo FB, Pereira SA, Madeira MF, Santos IB, Andrade MV, Cuzzi T, Marzochi MC, Schubach A: **American cutaneous leishmaniasis in two cats from Rio de Janeiro, Brazil: first report of natural infection with *Leishmania* (*Viannia*) *braziliensis***. *Trans R Soc Trop Med Hyg* 2004, **98:**165–167.
141. Souza AI, Barros EM, Ishikawa E, Ilha IM, Marin GR, Nunes VL: **Feline leishmaniasis due to *Leishmania* (*Leishmania*) *amazonensis* in Mato Grosso do Sul State, Brazil**. *Vet Parasitol* 2005, **128:**41–45.
142. Criado-Fornelio A, Ruas JL, Casado N, Farias NA, Soares MP, Müller G, Brumt JG, Berne ME, Buling-Saraña A, Barba-Carretero JC: **New molecular data on mammalian *Hepatozoon* species (Apicomplexa: Adeleorina) from Brazil and Spain**. *J Parasitol* 2006, **92:**93–99.
143. Rubini AS, Dos Santos Paduan K, Perez RR, Ribolla PE, O'Dwyer LH: **Molecular characterization of feline *Hepatozoon* species from Brazil**. *Vet Parasitol* 2006, **137:**168–171.
144. Coelho WM, Lima VM, Amarante AF, Langoni H, Pereira VB, Abdelnour A, Bresciani KD: **Occurrence of *Leishmania* (*Leishmania*) *chagasi* in a domestic cat**

(***Felis catus***) **in Andradina, São Paulo, Brazil: case report**. *Rev Bras Parasitol Vet* 2010, **19:**256–258.

145. Coelho WM, Richini-Pereira VB, Langoni H, Bresciani KD: **Molecular detection of *Leishmania* sp. in cats (*Felis catus*) from Andradina Municipality, São Paulo State, Brazil**. *Vet Parasitol* 2011, **176:**281–282.
146. de Bortoli CP, André MR, Braga Mdo S, Machado RZ: **Molecular characterization of *Hepatozoon* sp. in cats from São Luís Island, Maranhão, Northeastern Brazil**. *Parasitol Res* 2011, **109:**1189–1192.
147. Vides JP, Schwardt TF, Sobrinho LS, Marinho M, Laurenti MD, Biondo AW, Leutenegger C, Marcondes M: ***Leishmania chagasi* infection in cats with dermatologic lesions from an endemic area of visceral leishmaniosis in Brazil**. *Vet Parasitol* 2011, **178:**22–28.
148. Maia LM, Cerqueira AM, de Barros Macieira D, de Souza AM, Moreira NS, da Silva AV, Messick JB, Ferreira RF, Almosny NR: ***Cytauxzoon felis* and '*Candidatus* Mycoplasma haemominutum' coinfection in a Brazilian domestic cat (*Felis catus*)**. *Rev Bras Parasitol Vet* 2013, **22:**289–291.
149. Coura-Vital W, Marques MJ, Veloso VM, Roatt BM, Aguiar-Soares RD, Reis LE, Braga SL, Morais MH, Reis AB, Carneiro M: **Prevalence and factors associated with *Leishmania infantum* infection of dogs from an urban area of Brazil as identified by molecular methods**. *PLoS Negl Trop Dis* 2011, **5:**e1291.
150. Teles NM, Agostini MA, Bigeli JG, Noleto RV, Oliveira JD, de Oliveira Junior WP: **Molecular and parasitological detection of *Leishmania* spp. in dogs caught in Palmas, TO, Brazil**. *Rev Bras Parasitol Vet* 2012, **21:**278–282.
151. Tomaz-Soccol V, Castro EA, Navarro IT, de Farias MR, de Souza LM, Carvalho Y, Bispo S, Membrive NA, Minozzo JC, Truppel J, Bueno W, Luz E: **Allochthonous cases of canine visceral leishmaniasis in Paraná, Brazil: epidemiological implications**. *Rev Bras Parasitol Vet* 2009, **18:**46–51.
152. Marcondes CB, Pirmez C, Silva ES, Laurentino-Silva V, Steindel M, Santos AJ, Smaniotto H, Silva CF, Schuck Neto VF, Donetto A: **A survey of visceral leishmaniasis in dogs from Santa Maria and neighbouring municipalities, State of Rio Grande do Sul**. *Rev Soc Bras Med Trop* 2003, **36:**499–501.
153. Salomón OD, Orellano PW: ***Lutzomyia longipalpis* in Clorinda, Formosa province, an area of potential visceral leishmaniasis transmission in Argentina**. *Mem Inst Oswaldo Cruz* 2005, **100:**475–476.
154. Brazil RP, Caballero NN, Hamilton JG: **Identification of the sex pheromone of *Lutzomyia longipalpis* (Lutz and Neiva, 1912) (Diptera: Psychodidae) from Asunción, Paraguay**. *Parasit Vectors* 2009, **2:**51.
155. Salomón OD, Basmajdian Y, Fernández MS, Santini MS: ***Lutzomyia longipalpis* in Uruguay: the first report and the potential of visceral leishmaniasis transmission**. *Mem Inst Oswaldo Cruz* 2011, **106:**381–382.
156. Oliveira AG, Galati EA, de Oliveira O, de Oliveira GR, Espindola IA, Dorval ME, Brazil RP: **Abundance of *Lutzomyia longipalpis* (Diptera: Psychodidae: Phlebotominae) and urban transmission of visceral leishmaniasis in Campo Grande, state of Mato Grosso do Sul, Brazil**. *Mem Inst Oswaldo Cruz* 2006, **101:**869–874.
157. Souza GD, Santos Ed, Andrade Filho JD: **The first report of the main vector of visceral leishmaniasis in America, *Lutzomyia longipalpis* (Lutz & Neiva) (Diptera: Psychodidae: Phlebotominae), in the state of Rio Grande do Sul, Brazil**. *Mem Inst Oswaldo Cruz* 2009, **104:**1181–1182.

158. Loretti AP, Barros SS: **Hemorrhagic disease in dogs infected with an unclassified intraendothelial piroplasm in southern Brazil**. *Vet Parasitol* 2005, **134:**193–213.
159. Silva AS, Martins DB, Soares JF, França RT: **Canine rangeliosis: the need for differential diagnosis**. *Parasitol Res* 2013, **112:**1329–1332.
160. Szabó MP, Pinter A, Labruna MB: **Ecology, biology and distribution of spotted-fever tick vectors in Brazil**. *Front Cell Infect Microbiol* 2013, **3:**27.
161. Burlini L, Teixeira KR, Szabó MP, Famadas KM: **Molecular dissimilarities of *Rhipicephalus sanguineus* (Acari: Ixodidae) in Brazil and its relation with samples throughout the world: is there a geographical pattern?** *Exp Appl Acarol* 2010, **50:**361–374.
162. Moraes-Filho J, Marcili A, Nieri-Bastos FA, Richtzenhain LJ, Labruna MB: **Genetic analysis of ticks belonging to the *Rhipicephalus sanguineus* group in Latin America**. *Acta Trop* 2011, **117:**51–55.
163. Alves LC, de Almeida Silva LV, Faustino MA, McCall JW, Supakonderj P, Labarthe NW, Sanchez M, Caires O: **Survey of canine heartworm in the city of Recife, Pernambuco, Brazil**. *Mem Inst Oswaldo Cruz* 1999, **94:**587–590.
164. Brito AC, Vila-Nova MC, Martins Rocha DA, Gomes Costa L, Pinheiro de Almeida WA, da Silva Viana L, Ramalho Lopes R Jr, Fontes G, da Rocha EM, Regis L: **Prevalence of canine filariasis by *Dirofilaria immitis* and *Dipetalonema reconditum* in Maceió, Alagoas State, Brazil**. *Cad Saude Publica* 2001, **17:**1497–504.
165. Linardi PM: **Endoparasitos de *Ctenocephalides felis felis* (Siphonaptera: Pulicidae) em Belo Horizonte, MG**. *Biológico* 2002, **64:**65.
166. Reifur L, Thomaz-Soccol V, Montiani-Ferreira F: **Epidemiological aspects of filariosis in dogs on the coast of Paraná state, Brazil: with emphasis on *Dirofilaria immitis***. *Vet Parasitol* 2004, **122:**273–286.
167. Avelar DM, Bussolotti AS, do Carmo A Ramos M, Linardi PM: **Endosymbionts of *Ctenocephalides felis felis* (Siphonaptera: Pulicidae) obtained from dogs captured in Belo Horizonte, Minas Gerais, Brazil**. *J Invertebr Pathol* 2007, **94:**149–152.
168. Dantas-Torres F, de Brito ME, Brandão-Filho SP: **Seroepidemiological survey on canine leishmaniasis among dogs from an urban area of Brazil**. *Vet Parasitol* 2006, **140:**54–60.
169. Figueredo LA, Dantas-Torres F, de Faria EB, Gondim LF, Simões-Mattos L, Brandão-Filho SP, Mota RA: **Occurrence of antibodies to *Neospora caninum* and *Toxoplasma gondii* in dogs from Pernambuco, Northeast Brazil**. *Vet Parasitol* 2008, **157:**9–13.
170. Santos JM, Dantas-Torres F, Mattos MR, Lino FR, Andrade LS, Souza RC, Brito FL, Brito ME, Brandão-Filho SP, Simões-Mattos L: **Prevalence of anti-*Leishmania* spp antibodies in dogs from Garanhuns, in the middle scrub zone (Agreste) of Pernambuco**. *Rev Soc Bras Med Trop* 2010, **43:**41–45.
171. Coelho WM, do Amarante AF, Apolinário JC, Coelho NM, de Lima VM, Perri SH, Bresciani KD: **Seroepidemiology of *Toxoplasma gondii, Neospora caninum,* and *Leishmania* spp. infections and risk factors for cats from Brazil**. *Parasitol Res* 2011, **109:**1009–1013.
172. Cardia DF, Camossi LG, Neto LD, Langoni H, Bresciani KD: **Prevalence of *Toxoplasma gondii* and *Leishmania* spp. infection in cats from Brazil**. *Vet Parasitol* 2013, **197:**634–637.
173. Nogueira CI, Mesquita LP, Abreu CC, Nakagaki KY, Seixas JN, Bezerra PS, Rocha CM, Guimaraes AM, Peconick AP, Varaschin MS: **Risk factors associated with seroprevalence of *Neospora caninum* in dogs from urban and rural areas of milk**

and coffee production in Minas Gerais state, Brazil. *Epidemiol Infect* 2013, **141:**2286–2293.

174. Labarthe N, Serrão ML, Melo YF, de Oliveira SJ, Lourenço-de-Oliveira R: **Mosquito frequency and feeding habits in an enzootic canine dirofilariasis area in Niterói, state of Rio de Janeiro, Brazil**. *Mem Inst Oswaldo Cruz* 1998, **93:**145–154.
175. Resende MC, Camargo MC, Vieira JR, Nobi RC, Porto MN, Oliveira CD, Pessanha JE, Cunha Mda C, Brandão ST: **Seasonal variation of *Lutzomyia longipalpis* in Belo Horizonte, State of Minas Gerais**. *Rev Soc Bras Med Trop* 2006, **39:**51–55.
176. Ximenes MF, Castellón EG, De Souza MF, Menezes AA, Queiroz JW, Macedo e Silva VP, Jerônimo SM: **Effect of abiotic factors on seasonal population dynamics of *Lutzomyia longipalpis* (Diptera: Psychodidae) in northeastern Brazil**. *J Med Entomol* 2006, **43:**990–995.
177. Oliveira AG, Galati EA, Fernandes CE, Dorval ME, Brazil RP: **Seasonal variation of *Lutzomyia longipalpis* (Lutz & Neiva, 1912) (Diptera: Psychodidae: Phlebotominae) in endemic area of visceral leishmaniasis, Campo Grande, state of Mato Grosso do Sul, Brazil**. *Acta Trop* 2008, **105:**55–61.
178. Amóra SS, Bevilaqua CM, Dias EC, Feijó FM, Oliveira PG, Peixoto GC, Alves ND, Oliveira LM, Macedo IT: **Monitoring of *Lutzomyia longipalpis* Lutz & Neiva, 1912 in an area of intense transmission of visceral leishmaniasis in Rio Grande do Norte, Northeast Brazil**. *Rev Bras Parasitol Vet* 2010, **19:**39–43.
179. Saraiva L, Andrade Filho JD, Falcão AL, de Carvalho DA, de Souza CM, Freitas CR, Gomes Lopes CR, Moreno EC, Melo MN: **Phlebotominae fauna (Diptera: Psychodidae) in an urban district of Belo Horizonte, Brazil, endemic for visceral leishmaniasis: characterization of favored locations as determined by spatial analysis**. *Acta Trop* 2011, **117:**137–145.
180. Costa PL, Dantas-Torres F, da Silva FJ, Guimarães VC, Gaudêncio K, Brandão-Filho SP: **Ecology of *Lutzomyia longipalpis* in an area of visceral leishmaniasis transmission in north-eastern Brazil**. *Acta Trop* 2013, **126:**99–102.
181. Reinhold-Castro KR, Fenelon VC, Rossi RM, Brito JE, Freitas JS, Teodoro U: **Impact of control measures and dynamics of sand flies in southern Brazil**. *J Vector Ecol* 2013, **38:**63–68.
182. Silveira JA, Passos LM, Ribeiro MF: **Population dynamics of *Rhipicephalus sanguineus* (Latrielle, 1806) in Belo Horizonte, Minas Gerais state, Brazil**. *Vet Parasitol* 2009, **161:**270–275.
183. Louly CCB, Fonseca IN, Oliveira VF, Linhares GFC, Menezes LB, Borges LMF: **Seasonal dynamics of *Rhipicephalus sanguineus* (Acari: Ixodidae) in dogs from a police unit in Goiania, Goias, Brazil**. *Cienc Rural* 2007, **37**:464–469.
184. Maia MG, Costa RT, Haddad JP, Passos LM, Ribeiro MF: **Epidemiological aspects of canine babesiosis in the semiarid area of the state of Minas Gerais, Brazil**. *Prev Vet Med* 2007, **79:**155–162.
185. Heukelbach J, Jackson A, Ariza L, Feldmeier H: **Prevalence and risk factors of hookworm-related cutaneous larva migrans in a rural community in Brazil**. *Ann Trop Med Parasitol* 2008, **102:**53–61.
186. Tiyo R, Guedes TA, Falavigna DL, Falavigna-Guilherme AL: **Seasonal contamination of public squares and lawns by parasites with zoonotic potential in southern Brazil**. *J Helminthol* 2008, **82:**1–6.
187. Andresiuk V, Sardella N, Denegri G: **Seasonal fluctuations in prevalence of dog intestinal parasites in public squares of Mar del Plata city, Argentina and its risk for humans**. *Rev Argent Microbiol* 2007, **39:**221–224.

188. Táparo CV, Perri SHV, Serrano ACM, Ishizaki MN, Costa TP, Amarante AFT, Bresciani KDS: **Comparison between coproparasitological techniques for the diagnosis of helminth eggs or protozoa oocysts in dogs**. *Rev Bras Parasitol Vet* 2006, **15:**1–5.
189. Mandarino-Pereira A, de Souza FS, Lopes CW, Pereira MJ: **Prevalence of parasites in soil and dog feces according to diagnostic tests**. *Vet Parasitol* 2010, **170**:176–181.
190. Katagiri S, Oliveira-Sequeira TC: **Comparison of three concentration methods for the recovery of canine intestinal parasites from stool samples**. *Exp Parasitol* 2010, **126:**214–216.
191. Gomes JF, Hoshino-Shimizu S, Dias LC, Araujo AJ, Castilho VL, Neves FA: **Evaluation of a novel kit (TF-Test) for the diagnosis of intestinal parasitic infections.** *J Clin Lab Anal* 2004, **18:**132–138.
192. Farias LN, Malgor R, Cassaravilla C, Bragança C, de la Rue ML: **Echinococcosis in southern Brazil: efforts toward implementation of a control program in Santana do Livramento, Rio Grande do Sul**. *Rev Inst Med Trop Sao Paulo* 2004, **46:**153–156.
193. Monteiro RM, Pena HF, Gennari SM, de Souza SO, Richtzenhain LJ, Soares RM: **Differential diagnosis of oocysts of *Hammondia*-like organisms of dogs and cats by PCR-RFLP analysis of 70-kilodalton heat shock protein (HSP70) gene**. *Parasitol Res* 2008, **103:**235-238.
194. Soares RM, Lopes EG, Keid LB, Sercundes MK, Martins J, Richtzenhain LJ: **Identification of *Hammondia heydorni* oocysts by a heminested-PCR (hnPCR-AP10) based on the *H. heydorni* RAPD fragment AP10**. *Vet Parasitol* 2011, **175:**168–172.
195. Dantas-Torres F, Figueredo LA: **Canine babesiosis: a Brazilian perspective**. *Vet Parasitol* 2006, **141:**197–203.
196. Trapp SM, Dagnone AS, Vidotto O, Freire RL, Amude AM, de Morais HS: **Seroepidemiology of canine babesiosis and ehrlichiosis in a hospital population**. *Vet Parasitol* 2006, **140:**223–230.
197. Guimarães AM, Rocha CM, Oliveira TM, Rosado IR, Morais LG, Santos RR: **Factors associated the seropositivity for *Babesia*, *Toxoplasma*, *Neospora* e *Leishmania* in dogs attended at nine veterinary clinics in the municipality of Lavras, MG**. *Rev Bras Parasitol Vet* 2009, **18**(Suppl 1):49–53.
198. Furuta PI, Oliveira TM, Theixeira MC, Rocha AG, Machado RZ, Tinucci-Costa MG: **Comparison between a soluble antigen-based ELISA and IFAT in detecting antibodies against *Babesia canis* in dogs**. *Rev Bras Parasitol Vet* 2009, **18:**41–45.
199. Ramos R, Ramos C, Araújo F, Oliveira R, Souza I, Pimentel D, Galindo M, Santana M, Rosas E, Faustino M, Alves L: **Molecular survey and genetic characterization of tick-borne pathogens in dogs in metropolitan Recife (north-eastern Brazil)**. *Parasitol Res* 2010, **107:**1115–1120.
200. Almeida AB, Sousa VR, Gasparetto ND, da Silva GF, Figueiredo FB, Dutra V, Nakazato L, Madeira MF: **Canine visceral leishmaniasis: diagnostic approaches based on polymerase chain reaction employing different biological samples**. *Diagn Microbiol Infect Dis* 2013, **76:**321–324.
201. Ferreira SA, Almeida GG, Silva SO, Vogas GP, Fujiwara RT, de Andrade AS, Melo MN: **Nasal, oral and ear swabs for canine visceral leishmaniasis diagnosis: new practical approaches for detection of *Leishmania infantum* DNA**. *PLoS Negl Trop Dis* 2013, **7:**e2150.

202. Paiva Cavalcanti M, Dantas-Torres F, da Cunha Gonçalves de Albuquerque S, Silva de Morais RC, de Brito ME, Otranto D, Brandão-Filho SP: **Quantitative real time PCR assays for the detection of *Leishmania* (*Viannia*) *braziliensis* in animals and humans**. *Mol Cell Probes* 2013, **27:**122–128.
203. Reis LE, Coura-Vital W, Roatt BM, Bouillet LE, Ker HG, Fortes de Brito RC, Resende DD, Carneiro M, Giunchetti RC, Marques MJ, Carneiro CM, Reis AB: **Molecular diagnosis of canine visceral leishmaniasis: A comparative study of three methods using skin and spleen from dogs with natural *Leishmania infantum* infection**. *Vet Parasitol* 2013, **197**:498–503.
204. Silva MA, Pedrosa Soares CR, Medeiros RA, Medeiros Z, de Melo FL: **Optimization of single-tube nested PCR for the diagnosis of visceral leishmaniasis**. *Exp Parasitol* 2013, **134:**206–210.
205. Madeira MF, de O Schubach A, Schubach TM, Pereira SA, Figueiredo FB, Baptista C, Leal CA, Melo CX, Confort EM, Marzochi MC: **Post mortem parasitological evaluation of dogs seroreactive for *Leishmania* from Rio de Janeiro, Brazil**. *Vet Parasitol* 2006, **138:**366–370.
206. Silva DA, Madeira MF, Teixeira AC, de Souza CM, Figueiredo FB: **Laboratory tests performed on *Leishmania* seroreactive dogs euthanized by the leishmaniasis control program**. *Vet Parasitol* 2011, **179:**257–261.
207. Cavalcanti A, Lobo R, Cupolillo E, Bustamante F, Porrozzi R: **Canine cutaneous leishmaniasis caused by neotropical *Leishmania infantum* despite of systemic disease: A case report**. *Parasitol Int* 2012, **61:**738–740.
208. Souza AI, Nunes VLB, Borralho VM, Ishikawa EAY: **Domestic feline cutaneous leishmaniasis in the municipality of Ribas do Rio Pardo, Mato Grosso do Sul state, Brazil: a case report**. *J Venom Anim Toxins incl Trop Dis* 2009, **15:**359–365.
209. Demeler J, Ramünke S, Wolken S, Ianiello D, Rinaldi L, Gahutu JB, Cringoli G, von Samson-Himmelstjerna G, Krücken J: **Discrimination of gastrointestinal nematode eggs from crude fecal egg preparations by inhibitor-resistant conventional and real-time PCR**. *PLoS One* 2013, **8:**e61285.
210. Deplazes P, van Knapen F, Schweiger A, Overgaauw PA: **Role of pet dogs and cats in the transmission of helminthic zoonoses in Europe, with a focus on echinococcosis and toxocarosis**. *Vet Parasitol* 2011, **182:**41–53.
211. Lucio-Forster A, Liotta JL, Yaros JP, Briggs KR, Mohammed HO, Bowman DD: **Morphological differentiation of eggs of *Ancylostoma caninum*, *Ancylostoma tubaeforme*, and *Ancylostoma braziliense* from dogs and cats in the United States**. *J Parasitol* 2012, **98:**1041–1044.
212. Taylor MA: **Recent developments in ectoparasiticides**. *Vet J* 2001, **161:**253–268.
213. Wall R: **Ectoparasites: future challenges in a changing world**. *Vet Parasitol* 2007, **148:**62–74.
214. Pollmeier M, Pengo G, Jeannin P, Soll M: **Evaluation of the efficacy of fipronil formulations in the treatment and control of biting lice, *Trichodectes canis* (De Geer, 1778) on dogs**. *Vet Parasitol* 2002, **107:**127–136.
215. Stanneck D, Kruedewagen EM, Fourie JJ, Horak IG, Davis W, Krieger KJ: **Efficacy of an imidacloprid/flumethrin collar against fleas, ticks, mites and lice on dogs**. *Parasit Vectors* 2012, **5:**102.
216. Kužner J, Turk S, Grace S, Soni-Gupta J, Fourie JJ, Marchiondo AA, Rugg D: **Confirmation of the efficacy of a novel fipronil spot-on for the treatment and control of fleas, ticks and chewing lice on dogs**. *Vet Parasitol* 2013, **193:**245–251.
217. Serra-Freire NM, Benigno RNM, Oliveira SA, Lopes LMS, Galvão G: ***Lynxacarus radovskyi* - diagnostic and treatment in cats from the Metropolitan region of

Belem City, Para State. *Rev Universidade Rural, Série Ciências da Vida* 2002, **22:**57–60.

218. Franco MB, Hamann W: **Doramectin in the treatment of dogs with sarcoptic mange and gastrintestinal nematodes**. *Arch Vet Sci* 2004, **9:**23–29.
219. Silva RPB, Belettini ST, Stel RF, Martins LA, Pachaly JR: **Canine dermodicosis and new treatment perspectives: review**. *Arq Cienc Vet Zool Unipar* 2008, **11:**139–151.
220. Otranto D, Wall R: **New strategies for the control of arthropod vectors of disease in dogs and cats**. *Med Vet Entomol* 2008, **22:**291–302.
221. Paz GF, Labruna MB, Leite RC: **Drop off rhythm of *Rhipicephalus sanguineus* (Acari: Ixodidae) of artificially infested dogs**. *Rev Bras Parasitol Vet* 2008, **17:**139–144.
222. Blagburn BL, Dryden MW: **Biology, treatment, and control of flea and tick infestations**. *Vet Clin North Am Small Anim Pract* 2009, **39:**1173–1200.
223. Oliveira Lima JW, de Góes Cavalcanti LP, Pontes RJ, Heukelbach J: **Survival of *Betta splendens* fish (Regan, 1910) in domestic water containers and its effectiveness in controlling *Aedes aegypti* larvae (Linnaeus, 1762) in Northeast Brazil**. *Trop Med Int Health* 2010, **15:**1525–1532.
224. Barbosa RM, Regis LN: **Monitoring temporal fluctuations of *Culex quinquefasciatus* using oviposition traps containing attractant and larvicide in an urban environment in Recife, Brazil**. *Mem Inst Oswaldo Cruz* 2011, **106:**451–415.
225. Ministério da Saúde: *Manual de vigilância e controle da leishmaniose visceral*. Brasília: Ministério da Saúde; 2006.
226. Ministério da Saúde: *Manual de Vigilância da leishmaniose tegumentar americana*. 2a ed. Brasília: Ministério da Saúde; 2007.
227. Alexander B, Barros VC, de Souza SF, Barros SS, Teodoro LP, Soares ZR, Gontijo NF, Reithinger R: **Susceptibility to chemical insecticides of two Brazilian populations of the visceral leishmaniasis vector *Lutzomyia longipalpis* (Diptera: Psychodidae)**. *Trop Med Int Health* 2009, **14:**1272–1277.
228. Belinato TA, Martins AJ, Valle D: **Fitness evaluation of two Brazilian *Aedes aegypti* field populations with distinct levels of resistance to the organophosphate temephos**. *Mem Inst Oswaldo Cruz* 2012, **107:**916–922.
229. Courtenay O, Gillingwater K, Gomes PA, Garcez LM, Davies CR: **Deltamethrin-impregnated bednets reduce human landing rates of sandfly vector *Lutzomyia longipalpis* in Amazon households**. *Med Vet Entomol* 2007, **21:**168–176.
230. Araujo JM, Araújo JV, Braga FR, Araújo DM, Ferreira SR, Soares FE, Benjamin LA: **Survival of *Pochonia chlamydosporia* in the gastrointestinal tract of experimentally treated dogs**. *Res Vet Sci* 2012, **93:**803–806.
231. Otranto D, Dantas-Torres F: **The prevention of canine leishmaniasis and its impact on public health**. *Trends Parasitol* 2013, **29:**339–345.
232. Dantas-Torres F, Latrofa MS, Weigl S, Tarallo VD, Lia RP, Otranto D: ***Hepatozoon canis* infection in ticks during spring and summer in Italy**. *Parasitol Res* 2012, **110:**695–698.
233. Giannelli A, Ramos RA, Di Paola G, Mencke N, Dantas-Torres F, Baneth G, Otranto D: **Transstadial transmission of *Hepatozoon canis* from larvae to nymphs of *Rhipicephalus sanguineus***. *Vet Parasitol* 2013, **196:**1–5.
234. Dantas-Torres F: **Canine leishmaniosis in South America**. *Parasit Vectors* 2009, **2**(Suppl 1):S1.
235. Bongiorno G, Paparcone R, Manzillo VF, Oliva G, Cuisinier AM, Gradoni L: **Vaccination with LiESP/QA-21 (CaniLeish®) reduces the intensity of infection in**

***Phlebotomus perniciosus* fed on *Leishmania infantum* infected dogs-A preliminary xenodiagnosis study**. *Vet Parasitol* 2013, **197:**691–695.
236. Otranto D, de Caprariis D, Lia RP, Tarallo V, Lorusso V, Testini G, Dantas-Torres F, Latrofa S, Diniz PP, Mencke N, Maggi RG, Breitschwerdt E, Capelli G, Stanneck D: **Prevention of endemic canine vector-borne diseases using imidacloprid 10% and permethrin 50% in young dogs: a longitudinal field study**. *Vet Parasitol* 2010, **172:**323–332.
237. Dantas-Torres F, Capelli G, Giannelli A, Ramos RA, Lia RP, Cantacessi C, de Caprariis D, De Tommasi AS, Latrofa MS, Lacasella V, Tarallo VD, Di Paola G, Qurollo B, Breitschwerdt E, Stanneck D, Otranto D: **Efficacy of an imidacloprid/flumethrin collar against fleas, ticks and tick-borne pathogens in dogs**. *Parasit Vectors* 2013, **6:**245.
238. Otranto D, Dantas-Torres F, de Caprariis D, Di Paola G, Tarallo VD, Latrofa MS, Lia RP, Annoscia G, Breitshwerdt EB, Cantacessi C, Capelli G, Stanneck D: **Prevention of canine leishmaniosis in a hyper-endemic area using a combination of 10% imidacloprid/4.5% flumethrin**. *PLoS One* 2013, **8:**e56374.
239. Beugnet F, Franc M: **Insecticide and acaricide molecules and/or combinations to prevent pet infestation by ectoparasites**. *Trends Parasitol* 2012, **28:**267–279.
240. Beugnet F, Delport P, Luus H, Crafford D, Fourie J: **Preventive efficacy of Frontline(®) Combo and Certifect(®) against *Dipylidium caninum* infestation of cats and dogs using a natural flea (*Ctenocephalides felis*) infestation model**. *Parasite* 2013, **20:**7.
241. Fourie JJ, Ollagnier C, Beugnet F, Luus HG, Jongejan F: **Prevention of transmission of *Ehrlichia canis* by *Rhipicephalus sanguineus* ticks to dogs treated with a combination of fipronil, amitraz and (S)-methoprene (CERTIFECT®)**. *Vet Parasitol* 2013, **193:**223–228.
242. Dantas-Torres F, Figueredo LA, Brandão-Filho SP: ***Rhipicephalus sanguineus* (Acari: Ixodidae), the brown dog tick, parasitizing humans in Brazil**. *Rev Soc Bras Med Trop* 2006, **39:**64–67.
243. Guglielmone AA, Beati L, Barros-Battesti DM, Labruna MB, Nava S, Venzal JM, Mangold AJ, Szabó MP, Martins JR, González-Acuña D, Estrada-Peña A: **Ticks (Ixodidae) on humans in South America**. *Exp Appl Acarol* 2006, **40:**83–100.
244. Louly CCB, Fonseca IN, Oliveira VF, Borges LMF: **Occurrence of *Rhipicephalus sanguineus* in workers of veterinary clinics and kennels from Goiânia county, Goiás, Brazil**. *Cienc Anim Bras* 2006, **7:**103–106.
245. Szabó MP, Labruna MB, Castagnolli KC, Garcia MV, Pinter A, Veronez VA, Magalhães GM, Castro MB, Vogliotti A: **Ticks (Acari: Ixodidae) parasitizing humans in an Atlantic rainforest reserve of Southeastern Brazil with notes on host suitability**. *Exp Appl Acarol* 2006, **39:**339–346.
246. Serra-Freire NM: **Occurrence of ticks (Acari: Ixodidae) on human hosts, in three municipalities in the State of Pará, Brazil**. *Rev Bras Parasitol Vet* 2010, **19:**141–147.
247. Borsoi ABP, Serra-Freire NM: **Parasitic relations between human beings and ticks in the Volta Redonda city, state of Rio de Janeiro, Brazil**. *Rev UNIABEU* 2012, 5:306–317.
248. Dantas-Torres F: **Rocky Mountain spotted fever**. *Lancet Infect Dis* 2007, **7:**724–732.

249. Perez M, Bodor M, Zhang C, Xiong Q, Rikihisa Y: **Human infection with *Ehrlichia canis* accompanied by clinical signs in Venezuela**. *Ann N Y Acad Sci* 2006, **1078:**110–117.
250. Vieira RF, Vieira TS, Nascimento DA, Martins TF, Krawczak FS, Labruna MB, Chandrashekar R, Marcondes M, Biondo AW, Vidotto O: **Serological survey of *Ehrlichia* species in dogs, horses and humans: zoonotic scenery in a rural settlement from southern Brazil**. *Rev Inst Med Trop Sao Paulo* 2013, **55:**335–340.
251. Saito TB, Cunha-Filho NA, Pacheco RC, Ferreira F, Pappen FG, Farias NA, Larsson CE, Labruna MB: **Canine infection by rickettsiae and ehrlichiae in southern Brazil**. *Am J Trop Med Hyg* 2008, **79:**102–108.
252. Santos F, Coppede JS, Pereira AL, Oliveira LP, Roberto PG, Benedetti RB, Zucoloto LB, Lucas F, Sobreira L, Marins M: **Molecular evaluation of the incidence of *Ehrlichia canis*, *Anaplasma platys* and *Babesia* spp. in dogs from Ribeirão Preto, Brazil**. *Vet J* 2009, **179:**145–148.
253. Silva JN, Almeida AB, Boa Sorte Eda C, Freitas AG, Santos LG, Aguiar DM, Sousa VR: **Seroprevalence anti-*Ehrlichia canis* antibodies in dogs of Cuiabá, Mato Grosso**. *Rev Bras Parasitol Vet* 2010, **19:**108–111.
254. Souza BM, Leal DC, Barboza DC, Uzêda RS, De Alcântara AC, Ferreira F, Labruna MB: **Prevalence of ehrlichial infection among dogs and ticks in Northeastern Brazil**. *Rev Bras Parasitol Vet* 2010, **19:**89–93.
255. Aguiar DM, Cavalcante GT, Pinter A, Gennari SM, Camargo LM, Labruna MB: **Prevalence of *Ehrlichia canis* (Rickettsiales: Anaplasmataceae) in dogs and *Rhipicephalus sanguineus* (Acari: Ixodidae) ticks from Brazil**. *J Med Entomol* 2007, **44:**126–132.
256. Dantas-Torres F: **The brown dog tick, *Rhipicephalus sanguineus* (Latreille, 1806) (Acari: Ixodidae): from taxonomy to control**. *Vet Parasitol* 2008, **152:**173–185.
257. Limongi JE, Silva JJ, Paula MBC, Mendes J: **Epidemiologic aspects of flea infestations in the urban area of Uberlândia, Minas Gerais, 2007-2010**. *Epidemiol Serv Saude* 2013, **22:**285–294.
258. Oliveira RP, Galvão MA, Mafra CL, Chamone CB, Calic SB, Silva SU, Walker DH: ***Rickettsia felis* in *Ctenocephalides* spp. fleas, Brazil**. *Emerg Infect Dis* 2002, **8:**317–319.
259. Parola P: ***Rickettsia felis*: from a rare disease in the USA to a common cause of fever in sub-Saharan Africa**. *Clin Microbiol Infect* 2011, **17:**996–1000.
260. Traversa D: **Pet roundworms and hookworms: a continuing need for global worming**. *Parasit Vectors* 2012, **5:**91.
261. Vidal JE, Sztajnbok J, Seguro AC: **Eosinophilic meningoencephalitis due to *Toxocara canis*: case report and review of the literature**. *Am J Trop Med Hyg* 2003, **69:**341–343.
262. Moreira-Silva SF, Rodrigues MG, Pimenta JL, Gomes CP, Freire LH, Pereira FE: ***Toxocariasis* of the central nervous system: with report of two cases**. *Rev Soc Bras Med Trop* 2004, **37:**169–174.
263. Musso C, Castelo JS, Tsanaclis AM, Pereira FE: **Prevalence of *Toxocara*-induced liver granulomas, detected by immunohistochemistry, in a series of autopsies at a Children's Reference Hospital in Vitoria, ES, Brazil**. *Virchows Arch* 2007, **450:**411–417.
264. Schuster A, Lesshafft H, Reichert F, Talhari S, de Oliveira SG, Ignatius R, Feldmeier H: **Hookworm-related cutaneous larva migrans in northern Brazil: resolution of clinical pathology after a single dose of ivermectin**. *Clin Infect Dis* 2013, **57:**1155–1157.

265. Fragoso RP, Monteiro MB, Lemos EM, Pereira FE: **Anti-*Toxocara* antibodies detected in children attending elementary school in Vitoria, State of Espírito Santo, Brazil: prevalence and associated factors**. *Rev Soc Bras Med Trop* 2011, **44:**461–466.
266. Souza RF, Dattoli VC, Mendonça LR, Jesus JR, Baqueiro T, Santana Cde C, Santos NM, Barrouin-Melo SM, Alcantara-Neves NM: **Prevalence and risk factors of human infection by *Toxocara canis* in Salvador, State of Bahia, Brazil**. *Rev Soc Bras Med Trop* 2011, **44:**516–519.
267. Mattia S, Colli CM, Adami CM, Guilherme GF, Nishi L, Rubinsky-Elefant G, Marchioro AA, Gomes ML, Falavigna-Guilherme AL: **Seroprevalence of *Toxocara* infection in children and environmental contamination of urban areas in Paraná State, Brazil**. *J Helminthol* 2012, **86:**440–445.
268. Mendonça LR, Veiga RV, Dattoli VC, Figueiredo CA, Fiaccone R, Santos J, Cruz ÁA, Rodrigues LC, Cooper PJ, Pontes-de-Carvalho LC, Barreto ML, Alcantara-Neves NM: ***Toxocara* seropositivity, atopy and wheezing in children living in poor neighbourhoods in urban Latin American**. *PLoS Negl Trop Dis* 2012, **6:**e1886.
269. Schoenardie ER, Scaini CJ, Brod CS, Pepe MS, Villela MM, McBride AJ, Borsuk S, Berne ME: **Seroprevalence of *Toxocara* infection in children from southern Brazil**. *J Parasitol* 2013, **99:**537–539.
270. Capuano DM, Rocha GM: **Environmental contamination by *Toxocara* sp. eggs in Ribeirão Preto, São Paulo State, Brazil**. *Rev Inst Med Trop Sao Paulo* 2005, **47:**223–226.
271. Cassenote AJ, Pinto Neto JM, Lima-Catelani AR, Ferreira AW: **Soil contamination by eggs of soil-transmitted helminths with zoonotic potential in the town of Fernandópolis, State of São Paulo, Brazil, between 2007 and 2008**. *Rev Soc Bras Med Trop* 2011, **44:**371–374.
272. Gallina T, Silva MA, Castro LL, Wendt EW, Villela MM, Berne ME: **Presence of eggs of *Toxocara* spp. and hookworms in a student environment in Rio Grande do Sul, Brazil**. *Rev Bras Parasitol Vet* 2011, **20:**176–177.
273. Marques JP, Guimarães CR, Boas AV, Carnaúba PU, Moraes J: **Contamination of public parks and squares from Guarulhos (São Paulo State, Brazil) by *Toxocara* spp. and *Ancylostoma* spp**. *Rev Inst Med Trop Sao Paulo* 2012, **54:**267–271.
274. Marchioro AA, Colli CM, Ferreira EC, Tiyo R, Mattia S, de Souza WF, Falavigna-Guilherme AL: **Identification of public areas with potential toxocariasis transmission risk using Geographical Information Systems**. *Acta Parasitol* 2013 **58:**328–333.
275. Artigas PT, Araújo P, Romiti N, Ruivo M: **Sobre um caso de parasitismo humano por *Lagochilascaris minor* Leiper, 1909, no Estado de São Paulo, Brasil**. *Rev Inst Med Trop Sao Paulo* 1968, **10:**78–83.
276. Veloso MG, Faria MC, de Freitas JD, Moraes MA, Gorini DF, de Mendonça JL: **Human lagochilascariasis. 3 cases encountered in the Federal District, Brazil**. *Rev Inst Med Trop Sao Paulo* 1992, **34:**587–591.
277. Aguilar-Nascimento JE, Silva GM, Tadano T, Valadares Filho M, Akiyama AM, Castelo A: **Infection of the soft tissue of the neck due to *Lagochilascaris minor***. *Trans R Soc Trop Med Hyg* 1993, **87:**198.
278. Vieira MA, de Oliveira JA, Ferreira LS, de Oliveira V, Barbosa CA: **A case report of human lagochilascariasis coming from Pará State, Brazil**. *Rev Soc Bras Med Trop* 2000, **33:**87–90.
279. Aquino RT, Magliari ME, Vital Filho J, Silva MA, Lima CA, Rocha AJ, Silva CJ, Rewin JA, Nahas TR, Chieffi PP: **Lagochilascariasis leading to severe involvement**

of ocular globes, ears and meninges. *Rev Inst Med Trop Sao Paulo* 2008, **50:**355–358.

280. Palheta-Neto FX, Leão RNQ, Neto HF, Tomita S, Lima MAMT, Pezzin-Palheta AC: **Contribution to the study of human lagochilascariasis**. *Rev Bras Otorrinolaringol* 2002 **68:**101–105.
281. Amato JF, Grisi L, Pimentel Neto M: **Two cases of fistulated abscesses caused by *Lagochilascaris major* in the domestic cat**. *Mem Inst Oswaldo Cruz* 1990, **85:**471–473.
282. Sudré AP, Uchôa F, Brener B: **Lagochilascariasis in a housecat and the potential risk for human disease**. *Braz J Infect Dis* 2012, **16:**111–112.
283. Faccio L, Oliveira CB, Denardin CA, Tonin AA, Gressler LT, Dalla Rosa L, Sampaio LC, Stainki DR, Monteiro SG. **Case report: Feline infection by *Lagochilascaris* sp. in the State of Rio Grande do Sul, Brazil**. *Vet Parasitol* 2013, **196:**541–543.
284. Barbosa CAL, Barbosa AP, Campos DMB: **Domestic cat (*Felis catus domesticus*) as a possible reservoir of *Lagochilascaris minor* Leiper, 1909**. *Rev Patol Trop* 2005, **34:**205–211.
285. Moura MQ, Jeske S, Gallina T, Borsuk S, Berne ME, Villela MM: **First report of *Lagochilascaris* (Nematoda: Ascarididae) eggs in a public park in Southern Brazil**. *Vet Parasitol* 2012, **184:**359–361.
286. Marinho RP, Neves DP: ***Dipylidium caninum* (Dilepididae-Cestoda). Report of 2 human cases**. *Rev Inst Med Trop Sao Paulo* 1979, **21:**266–268.
287. Lemos CH, Oliveira CR: **Infestação humana pelo *Dipylidium caninum***. *Rev Soc Bras Med Trop* 1985, **18:**267–268.
288. Maia MA, Campos DMB, Damasceno FA: ***Dipylidium caninum* (Cestoda-Dilepididae). Report of one human case in Goiânia, Goiás, Brazil**. *Rev Patol Trop* 1991, **20**:7–12.
289. Larrieu E, Zanini F: **Critical analysis of cystic echinococcosis control programs and praziquantel use in South America, 1974-2010**. *Rev Panam Salud Publica* 2012, **31:**81–87.
290. de la Rue ML: **Cystic echinococcosis in southern Brazil**. *Rev Inst Med Trop Sao Paulo* 2008, **50:**53–56.
291. Moraes MA, Sobreira MN, Medeiros Filho P, Tavares AC, Gomes MI: **Polycystic hydatidosis: casual finding of calcified hydatid cyst simulating mesenteric neoplasm**. *Rev Soc Bras Med Trop* 2003, **36:**519–521.
292. D'Alessandro A, Rausch RL: **New aspects of neotropical polycystic (*Echinococcus vogeli*) and unicystic (*Echinococcus oligarthrus*) echinococcosis**. *Clin Microbiol Rev* 2008, **21:**380–401.
293. Eckert J, Deplazes P: **Biological, epidemiological, and clinical aspects of echinococcosis, a zoonosis of increasing concern**. *Clin Microbiol Rev* 2004, **17**:107–135.
294. Dantas-Torres F, Otranto D: **Dirofilariosis in the Americas: a more virulent *Dirofilaria immitis*?** *Parasit Vectors* 2013, **6**:288.
295. Milanez de Campos JR, Barbas CS, Filomeno LT, Fernandez A, Minamoto H, Filho JV, Jatene FB: **Human pulmonary dirofilariasis: analysis of 24 cases from São Paulo, Brazil**. *Chest* 1997, **112**:729–733.
296. Rodrigues-Silva R, Moura H, Dreyer G, Rey L: **Human pulmonary dirofilariasis: a review**. *Rev Inst Med Trop Sao Paulo* 1995, **37**:523–530.
297. Cavallazzi R, Cavallazzi A, Souza I, Cardoso J: **Dirofilariose pulmonar humana: relato de sete casos**. *J Pneumol* 2002, **28:**100–102.

298. Rodrigues-Silva R, Guerra RJ, de Almeida FB, Machado-Silva JR, de Paiva DD: **Human pulmonary dirofilariasis at Rio de Janeiro, Brazil: a case report**. *Rev Soc Bras Med Trop* 2004, **37**:56–59.
299. Otranto D, Diniz DG, Dantas Torres F, Casiraghi M, De Almeida IN, De Almeida LN, Dos Santos JN, Furtado AP, De Almeida Sobrinho EF, Bain O: **Human intraocular filariasis caused by *Dirofilaria* sp. nematode, Brazil**. *Emerg Infect Dis* 2011, **17**:863–866.
300. Dantas-Torres F, Solano-Gallego L, Baneth G, Ribeiro VM, de Paiva-Cavalcanti M, Otranto D: **Canine leishmaniosis in the Old and New Worlds: unveiled similarities and differences**. *Trends Parasitol* 2012, **28**:531–538.
301. Maia-Elkhoury AN, Alves WA, Sousa-Gomes ML, Sena JM, Luna EA: **Visceral leishmaniasis in Brazil: trends and challenges**. *Cad Saude Publica* 2008, **24**:2941–2947.
302. Costa CH, Stewart JM, Gomes RB, Garcez LM, Ramos PK, Bozza M, Satoskar A, Dissanayake S, Santos RS, Silva MR, Shaw JJ, David JR, Maguire JH: **Asymptomatic human carriers of *Leishmania chagasi***. *Am J Trop Med Hyg* 2002, **66**:334–337.
303. Silveira FT, Lainson R, Crescente JA, de Souza AA, Campos MB, Gomes CM, Laurenti MD, Corbett CE: **A prospective study on the dynamics of the clinical and immunological evolution of human *Leishmania* (*L.*) *infantum chagasi* infection in the Brazilian Amazon region**. *Trans R Soc Trop Med Hyg* 2010, **104**:529–535.
304. Lima ID, Queiroz JW, Lacerda HG, Queiroz PV, Pontes NN, Barbosa JD, Martins DR, Weirather JL, Pearson RD, Wilson ME, Jeronimo SM: ***Leishmania infantum chagasi* in northeastern Brazil: asymptomatic infection at the urban perimeter**. *Am J Trop Med Hyg* 2012, **86**:99–107.
305. Alvarenga DG, Escalda PM, Costa AS, Monreal MT: **Visceral leishmaniasis: retrospective study on factors associated with lethality**. *Rev Soc Bras Med Trop* 2010, **43:**194–197.
306. Madalosso G, Fortaleza CM, Ribeiro AF, Cruz LL, Nogueira PA, Lindoso JA: **American visceral leishmaniasis: factors associated with lethality in the state of São Paulo, Brazil**. *J Trop Med* 2012, **2012:**281572.
307. Barata RA, Peixoto JC, Tanure A, Gomes ME, Apolinário EC, Bodevan EC, de Araújo HS, Dias ES, Pinheiro AC: **Epidemiology of visceral leishmaniasis in a reemerging focus of intense transmission in Minas Gerais state, Brazil**. *Biomed Res Int* 2013, **2013:**405083.
308. Dubey JP, Lago EG, Gennari SM, Su C, Jones JL: **Toxoplasmosis in humans and animals in Brazil: high prevalence, high burden of disease, and epidemiology**. *Parasitology* 2012, **139:**1375–1424.
309. Ekman CC, Chiossi MF, Meireles LR, Andrade Júnior HF, Figueiredo WM, Marciano MA, Luna EJ: **Case-control study of an outbreak of acute toxoplasmosis in an industrial plant in the state of São Paulo, Brazil**. *Rev Inst Med Trop Sao Paulo* 2012, **54**:239–244.
310. Xavier GA, Cademartori BG, Cunha Filho NA, Farias NA: **Evaluation of seroepidemiological toxoplasmosis in HIV/AIDS patients in the south of Brazil**. *Rev Inst Med Trop Sao Paulo* 2013, **55:**25–30.
311. Hotez PJ, Dumonteil E, Heffernan MJ, Bottazzi ME: **Innovation for the 'bottom 100 million': eliminating neglected tropical diseases in the Americas**. *Adv Exp Med Biol* 2013, **764:**1–12.
312. Coura JR, Dias JC: **Epidemiology, control and surveillance of Chagas disease: 100 years after its discovery**. *Mem Inst Oswaldo Cruz* 2009, **104**(Suppl 1):31-40.

313. Deane LM: **Animal reservoirs of *Trypanosoma cruzi* in Brazil**. *Rev Bras Malariol Doenças Trop* 1964, **16:**27–48.
314. Alencar JE, Almeida YM, Santos AR, Freitas LM: **Epidemiology of Chagas' disease in the state of Ceará, Brazil**. *Rev Bras Malariol D Trop* 1975, **26:**5–26.
315. Mott KE, Mota EA, Sherlock I, Hoff R, Muniz TM, Oliveira TS, Draper CC: ***Trypanosoma cruzi* infection in dogs and cats and household seroreactivity to *T. cruzi* in a rural community in northeast Brazil**. *Am J Trop Med Hyg* 1978, **27:**1123–1127.
316. Barrett TV, Hoff R, Mott KE, Guedes F, Sherlock IA: **An outbreak of acute Chagas's disease in the São Francisco Valley region of Bahia, Brazil: triatomine vectors and animal reservoirs of *Trypanosoma cruzi***. *Trans R Soc Trop Med Hyg* 1979, **73:**703–709.
317. Maywald PG, Machado MI, Costa-Cruz JM, Gonçalves-Pires M: **Leishmaniose tegumentar, visceral e doença de Chagas caninas em municípios do Triângulo Mineiro e Alto Paranaíba, Minas Gerais, Brasil**. *Cad Saude Publica* 1996, **12:**321–328.
318. Herrera L, D'Andrea PS, Xavier SC, Mangia RH, Fernandes O, Jansen AM: ***Trypanosoma cruzi* infection in wild mammals of the National Park 'Serra da Capivara' and its surroundings (Piauí, Brazil), an area endemic for Chagas disease**. *Trans R Soc Trop Med Hyg* 2005, **99:**379–388.
319. Souza-Lima RD, Barbosa MD, Coura JR, Arcanjo AR, Nascimento AD, Ferreira JM, Magalhães LK, Albuquerque BC, Araújo GA, Guerra JA: **Outbreak of acute Chagas disease associated with oral transmission in the Rio Negro region, Brazilian Amazon**. *Rev Soc Bras Med Trop* 2013, **46:**510–514.
320. Cerqueira EJ, Silva EM, Monte-Alegre AF, Sherlock IA: **Notes on fleas (Siphonaptera) of the fox *Cerdocyon thous* (Canidae) from an endemic area of visceral leishmaniasis in Jacobina, Bahia, Brazil**. *Rev Soc Bras Med Trop* 2000, **33:**91–93.
321. Labruna MB, Jorge RS, Sana DA, Jácomo AT, Kashivakura CK, Furtado MM, Ferro C, Perez SA, Silveira L, Santos TS Jr, Marques SR, Morato RG, Nava A, Adania CH, Teixeira RH, Gomes AA, Conforti VA, Azevedo FC, Prada CS, Silva JC, Batista AF, Marvulo MF, Morato RL, Alho CJ, Pinter A, Ferreira PM, Ferreira F, Barros-Battesti DM: **Ticks (Acari: Ixodida) on wild carnivores in Brazil**. *Exp Appl Acarol* 2005, **36:**149–163.
322. Dantas-Torres F, Ferreira DR, Melo LM, Lima PA, Siqueira DB, Rameh-de-Albuquerque LC, de Melo AV, Ramos JA: **Ticks on captive and free-living wild animals in northeastern Brazil**. *Exp Appl Acarol* 2010, **50:**181–189.
323. Martins JR, Reck J Jr, Doyle RL, Cruz NL, Vieira AW, Souza UA: ***Amblyomma aureolatum* (Acari: Ixodidae) parasitizing margay (*Leopardus wiedii*) in Rio Grande do Sul**. *Rev Bras Parasitol Vet* 2010, **19:**189–191.
324. Ruas JL, Muller G, Farias NA, Gallina T, Lucas AS, Pappen FG, Sinkoc AL, Brum JG: **Helminths of Pampas fox, *Pseudalopex gymnocer*cus (Fischer, 1814) and of Crab-eating fox, *Cerdocyon thous* (Linnaeus, 1766) in the South of the State of Rio Grande do Sul, Brazil**. *Rev Bras Parasitol Vet* 2008, **17:**87–92.
325. Ribeiro CT, Verocai GG, Tavares LE: ***Dioctophyme renale* (Nematoda, Dioctophymatidae) infection in the crab-eating fox (*Cerdocyon thous*) from Brazil**. *J Wildl Dis* 2009, **45:**248–250.
326. Vieira FM, Luque JL, Lima SS, Neto AH, Muniz-Pereira LC: ***Dipylidium caninum* (Cyclophyllidea, Dipylidiidae) in a wild carnivore from Brazil**. *J Wildl Dis* 2012, **48:**233–234.

327. Megid J, de Souza VA, Teixeira CR, Cortez A, Amorin RL, Heinemman MB, Cagnini DQ, Richtzenhain LJ: **Canine distemper virus in a crab-eating fox (*Cerdocyon thous*) in Brazil: case report and phylogenetic analyses**. *J Wildl Dis* 2009, **45:**527-530.
328. Megid J, Teixeira CR, Amorin RL, Cortez A, Heinemann MB, Antunes JMP, Costa LF, Fornazari F, Cipriano JR, Cremasco A, Richtzenhain LJ: **First identification of canine distemper virus in hoary fox (*Lycalopex vetulus*): pathologic aspects and virus phylogeny**. *J Wildl Dis* 2010, **46:**303–305.
329. Luppi MM, Malta MC, Silva TM, Silva FL, Motta RO, Miranda I, Ecco R, Santos RL: **Visceral leishmaniasis in captive wild canids in Brazil**. *Vet Parasitol* 2008, **155:**146–151.
330. Vieira FM, Luque JL, Muniz-Pereira LC: **Checklist of helminth parasites in wild carnivore mammals from Brazil**. *Zootaxa* 2008, **1721:**1–23.
331. Soares RM, Cortez LR, Gennari SM, Sercundes MK, Keid LB, Pena HF: **Crab-eating fox (*Cerdocyon thous*), a South American canid, as a definitive host for *Hammondia heydorni***. *Vet Parasitol* 2009, **162:**46–50.
332. Tenório MS, Oliveira e Sousa L, Paixão MS, Alves MF, Paulan SC, Lima FL, Jusi MM, Tasca KI, Machado RZ, Starke-Buzetti WA: **Visceral leishmaniasis in a captive crab-eating fox *Cerdocyon thous***. *J Zoo Wildl Med* 2011, **42:**608–616.
333. Almeida Curi NH, Coelho CM, de Campos Cordeiro Malta M, Magni EM, Sábato MA, Araújo AS, Lobato ZI, Santos JL, Santos HA, Ragozo AA, de Souza SL: **Pathogens of wild maned wolves (*Chrysocyon brachyurus*) in Brazil**. *J Wildl Dis* 2012, **48:**1052-1056.
334. Ministério da Agricultura, Pecuária e Abastecimento: *Legislação relacionada aos produtos de uso veterinário*. Brasília: MAPA/ACS; 2012.
335. Forlano M, Scofield A, Elisei C, Fernandes KR, Ewing SA, Massard CL: **Diagnosis of *Hepatozoon* spp. in *Amblyomma ovale* and its experimental transmission in domestic dogs in Brazil**. *Vet Parasitol* 2005, **134:**1–7.
336. Rubini AS, Paduan KS, Martins TF, Labruna MB, O'Dwyer LH: **Acquisition and transmission of *Hepatozoon canis* (Apicomplexa: Hepatozoidae) by the tick *Amblyomma ovale* (Acari: Ixodidae)**. *Vet Parasitol* 2009, **164:**324–327
337. de Miranda RL, de Castro JR, Olegário MM, Beletti ME, Mundim AV, O'Dwyer LH, Eyal O, Talmi-Frank D, Cury MC, Baneth G: **Oocysts of *Hepatozoon canis* in *Rhipicephalus* (*Boophilus*) *microplus* collected from a naturally infected dog**. *Vet Parasitol* 2011, **177:**392–396.
338. Almeida GLG, Vicente JJ: ***Cercopithifilaria bainae* sp. n. parasita de *Canis familiaris* (L.) (Nematoda, Filarioidea)**. *Atas Soc Biol Rio de Janeiro* 1984, **24:**18.
339. Otranto D, Brianti E, Latrofa MS, Annoscia G, Weigl S, Lia RP, Gaglio G, Napoli E, Giannetto S, Papadopoulos E, Mirò G, Dantas-Torres F, Bain O: **On a *Cercopithifilaria* sp. transmitted by *Rhipicephalus sanguineus*: a neglected, but widespread filarioid of dogs**. *Parasit Vectors* 2012, **5:**1.
340. Otranto D, Varcasia A, Solinas C, Scala A, Brianti E, Dantas-Torres F, Annoscia G, Martin C, Mutafchiev Y, Bain O: **Redescription of *Cercopithifilaria bainae***

Almeida & Vicente, 1984 (Spirurida, Onchocercidae) from a dog in Sardinia, Italy. *Parasit Vectors* 2013, **6:**132.
341. Latrofa MS, Dantas-Torres F, Annoscia G, Cantacessi C, Otranto D: **Comparative analyses of mitochondrial and nuclear genetic markers for the molecular identification of *Rhipicephalus* spp**. *Infect Genet Evol*, in press.
342. Jefferies R, Shaw SE, Viney ME, Morgan ER: ***Angiostrongylus vasorum* from South America and Europe represent distinct lineages**. *Parasitology* 2009, **136:**107–115.
343. Marcondes M, de Lima VM, de Araújo MD, Hiramoto RM, Tolezano JE, Vieira RF, Biondo AW: **Longitudinal analysis of serological tests officially adopted by the Brazilian Ministry of Health for the diagnosis of canine visceral leishmaniasis in dogs vaccinated with Leishmune®**. *Vet Parasitol* 2013, **197:**649–652.
344. Franken AMJ, Figueiredo MJ, Coutinho SG: **Prevalência de *Isospora* em cães de diferentes áreas da cidade do Rio de Janeiro**. *Rev Soc Bras Med Trop* 1975, **9:**45–51.
345. Ogassawara S, Larsson CE, Larsson MHMA, Hagiwara MK: **Incidence of *Isospora* sp. in dog of the city of São Paulo**. *Rev Fac Med Vet Zootec Univ S Paulo* 1978, **15:**137–142.
346. Pena HF, Ogassawara S, Sinhorini IL: **Occurrence of cattle *Sarcocystis* species in raw kibbe from Arabian food establishments in the city of São Paulo, Brazil, and experimental transmission to humans**. *J Parasitol* 2001, **87:**1459–1465.
347. Ruas JL, Cunha CW, Silva SS: **Prevalência de *Sarcocystis* spp. (Lankester, 1882) em bovinos clinicamente sadios, da Região Sul do Rio Grande do Sul, Brasil**. *Rev Bras Agrociência* 2001, **7:**227–230.
348. Lopes CW, de Sá WF, Botelho GG: **Lesions in cross-breed pregnant cows, experimentally infected with *Sarcocystis cruzi* (Hasselmann, 1923) Wenyon, 1926 (Apicomplexa: Sarcocytidae)**. *Rev Bras Parasitol Vet* 2005, **14:**79–83.
349. Funada MR, Pena HFJ, Soares RM, Amaku M, Gennari SM: **Frequency of gastrointestinal parasites in dogs and cats referred to a veterinary school hospital in the city of São Paulo**. *Arq Bras Med Vet Zootec* 2007, **59:**1338–1340.
350. Santos FAG, Yamamura MH, Vidotto O, Camargo PL: **Occurrence of gastrointestinal parasites in dogs (*Canis familiaris*) with acute diarrhea from metropolitan region of Londrina, Parana State, Brazil**. *Semina: Ciências Agrárias* 2007, **28:**257–268.
351. Silva RC, Su C, Langoni H: **First identification of *Sarcocystis tenella* (Railliet, 1886) Moulé, 1886 (Protozoa: Apicomplexa) by PCR in naturally infected sheep from Brazil**. *Vet Parasitol* 2009, **165:**332–336.
352. Monteiro RM, Richtzenhain LJ, Pena HF, Souza SL, Funada MR, Gennari SM, Dubey JP, Sreekumar C, Keid LB, Soares RM: **Molecular phylogenetic analysis in *Hammondia*-like organisms based on partial Hsp70 coding sequences**. *Parasitology* 2007, **134:**1195–1203.
353. Coelho WMD, Amarante AFT, Perri SHV, Coelho NMD, Apolinário JC, Teixeira WFP, Brescian KDS: **Coccidiosis in dogs and cats at the municipality of Andradina in the State of São Paulo, Brazil**. *Braz J Vet Res Anim Sci* 2012, **49:**162–166.

354. Ragozo AMA, Muradian V, Silva JCR, Caravieri R, Amajoner VR, Magnabosco C, Gennari SM: **Occurrence of gastrointestinal parasites in feces of cats from the cities of São Paulo and Guarulhos**. *Braz J Vet Res Anim Sci* 2002, **39:**244–246.
355. Nery-Guimarães F, Lage HA: **Prevalence and intestinal cycle of *Isospora felis* Wenyon, 1923 e *I. rivolta* (Grassi, 1879) Wenyon, 1923 in the domestic cat**. *Mem Inst Oswaldo Cruz* 1973, **71:**43–54.
356. Togni M, Panziera W, Souza TM, Oliveira Filho JC, Mazzanti A, Barros CSL, Fighera RA: **Aspectos epidemiológicos, clínicos e anatomopatológicos da infecção por *Gurltia paralysans* em gatos**. *Pesq Vet Bras* 2013, **33**:363–371.

Figuras

Figura 1 – Ligação homem-animal

Um morador de rua e seus amigos inseparáveis que foram encontrados abandonados nas ruas de Porto Alegre, sul do Brasil.

Figura 2 – Precárias condições de vida

Um proprietário e seus cães vivendo em uma pobre comunidade rural em Goiana, nordeste do Brasil, onde a leishmaniose visceral é endêmica. Em pobres comunidades rurais pessoas (como esse homem da foto) estão acostumados a caminhar descalços, o que pode ser um fator de risco para doenças com *larva migrans* cutânea e tungíase.

Figura 3 – Ectoparasitos de cães e gatos

Desenhos para a identificação dos ectoparasitos mais comuns de cães e gatos (pulgas: A-F; piolhos: G-J; e ácaros: K-P) encontrados no Brasil. Para maiores detalhes, *vide* Tabela 3.

Figura 4 – Densidade do vector

Centenas de flebotomíneos (*Lutzomyia longipalpis*) sobre uma galinha (A) que estava repousando sobre um tronco de uma árvore em Passira, nordeste do Brasil, onde a leishmaniose visceral é endêmica. A Figura 4B mostra um *close-up* dos flebotomíneos.

Figura 5 – Contaminação ambiental com fezes de cães

Cães de rua passeando livremente em uma praia no sul do Brasil. Fezes de cães são uma fonte importante de parasitos zoonóticos que podem causar doenças como *larva migrans* cutânea.

Tabela 1 - Ectoparasitos de cães no Brasil.

Filo	Classe	Ordem	Família	Espécie[a]
Arthropoda	Arachnida	Ixodida	Argasidae	*Ornithodoros brasiliensis*
				Ornithodoros rostratus
			Ixodidae	*Amblyomma aureolatum*
				Amblyomma cajennense
				Amblyomma longirostre
				Amblyomma naponsense
				Amblyomma nodosum
				Amblyomma oblongoguttatum
				Amblyomma ovale
				Amblyomma pacae
				Amblyomma parvum
				Amblyomma scalpturatum
				Amblyomma tigrinum
				Haemaphysalis juxtakochi
				Rhipicephalus microplus
				Rhipicephalus sanguineus s.l.
		Sarcoptiformes	Psoroptidae	*Otodectes cynotis*
			Sarcoptidae	*Notoedres cati*
				Sarcoptes scabiei
		Trombidiformes	Demodicidae	*Demodex canis*
	Insecta	Phthiraptera	Boopidae	*Heterodoxus spiniger*
			Linognathidae	*Linognathus setosus*
			Trichodectidae	*Trichodectes canis*
		Siphonaptera	Ceratophyllidae	*Nosopsyllus fasciatus*
			Tungidae	*Tunga penetrans*
			Pulicidae	*Ctenocephalides canis*
				Ctenocephalides felis felis
				Pulex irritans
				Xenopsylla brasiliensis
				Xenopsylla cheopis
			Rhopalopsyllidae	*Rhopalopsyllus lutzi lutzi*

[a] Lista de ectoparasitos (excluíndo insetos voadores) que infestão cães no Brasil [11-41].

Tabela 2 - Ectoparasitos de gatos no Brasil.

**Filo**	**Classe**	**Ordem**	**Família**	**Espécie**[a]
Arthropoda	Arachnida	Ixodida	Ixodidae	*Amblyomma aureolatum*
				Amblyomma ovale
				Amblyomma triste
				Rhipicephalus sanguineus s.l.
		Sarcoptiformes	Listrophoridae	*Lynxacarus radovskyi*
			Psoroptidae	*Otodectes cynotis*
			Sarcoptidae	*Notoedres cati*
				Sarcoptes scabiei
		Trombidiformes	Demodicidae	*Demodex cati*
	Insecta	Phthiraptera	Trichodectidae	*Felicola subrostratus*
		Siphonaptera	Pulicidae	*Ctenocephalides canis*
				Ctenocephalides felis felis
				Pulex irritans
			Tungidae	*Tunga penetrans*

[a] Lista de ectoparasitos (excluíndo insetos voadores) que infestão gatos no Brasil [19, 22, 42-49].

Tabela 3 – Chave para ectoparasitos de cães e gatos.

1a	Três pares de pernas	**2**
1b	Quatro pares de pernas	**13**
2a	Corpo achatado lateralmente (SIPHONAPTERA)	**3**
2b	Corpo achatado dorsoventralmente (PHTHIRAPTERA)	**10**
3a	Ctenídeos pronotal e/ou genal presentes	**4**
3b	Ctenídeos pronotal e genal ausentes	**6**
4a	Ctenídeo genal ausente e pronotal presente	***Nosopsyllus fasciatus***
4b	Ctenídeos genal e pronotal presentes	**5**
5a	Ctenídio genal com a primeira cerda bem mais curta que as demais (Figura 3A; ponta de seta); tíbia posterior com duas cerdas simples entre a penúltima e a última cerdas duplas (Figura 3A; setas)	***Ctenocephalides canis***
5b	Ctenídio genal com a primeira cerda aproximadamente do mesmo comprimento das demais (Figura 3B; ponta de seta); tíbia posterior com uma cerda simples entre a penúltima e a última cerdas duplas (Figura 3B; seta)	***Ctenocephalides felis felis***
6a	Segmentos torácicos estreitos (Figura 3C; seta); cerdas oculares e occipitais ausentes; cabeça com porção antero-dorsal arrebitada (Figura 3C; ponta de seta)	***Tunga penetrans***
6b	Não como descrito acima	**7**
7a	Segmentos abdominais com duas fileiras de cerdas na borda dorsal	***Rhopalopsyllus lutzi lutzi***
7b	Segmentos abdominais com uma fileira de cerdas na borda dorsal	**8**
8a	Região occipital com uma cerda (Figura 3D; seta)	***Pulex irritans***
8b	Região occipital com duas fileiras de cerdas dispostas em "V"	**9**
9a	Macho com cerda antepigidial implantada em discreto tubérculo; fêmea com o corpo da espermateca (em preto) não mais largo que a base da cauda (Figura 3E)	***Xenopsylla cheopis***
9b	Macho com cerda antepigidial implantada em nítido tubérculo; fêmea com o corpo da espermateca (em preto) mais largo que a base da cauda (Figura 3F)	***Xenopsylla brasiliensis***
10a	Cabeça mais longa que larga (Figura 3G; seta); aparelho bucal sugador	***Linognathus setosus***
10b	Cabeça tão larga quanto longa; aparelho bucal mastigador	**11**
11a	Tarso com duas garras (Figura 3H; seta)	***Heterodoxus spiniger***
11b	Tarso com uma garra	**12**
12a	Cabeça arredondada anteriormente (Figura 3I)	***Trichodectes canis***
12b	Cabeça triangular (Figura 3J)	***Felicola subrostratus***
13a	Corpo igual ou menor 0.5 mm; órgão de Haller ausente	**14**
13b	Corpo maior que 0.5 mm; órgão de Haller presente (IXODIDA)	**19**
14a	Em forma de cenoura ou pimentão (TROMBIDIFORMES)	**15**
14b	Corpo não como acima, geralmente arredondado (SARCOPTIFORMES)	**16**
15a	Corpo longo e delgado (em forma de cenoura) (Figura 3K)	***Demodex* spp.** [a]
15b	Corpo em forma de pimentão; peças bucais bem desenvolvidas; palpos artículos terminais em forma de garra (Figura 3L; seta)	***Cheyletiella* spp.** [b]
16a	Corpo globoso	**17**
16b	Corpo cilíndrico e alongado, fortemente estriado (Figura 3M)	***Lynxacarus radovskyi***
17a	Pernas longas, com pedicelos curtos e não-articulados (Figura 3N)	***Otodectes cynotis***
17b	Pernas curtas, com pedicelos longos e não-articulados	**18**
18a	Ânus dorsal (Figura 3O; seta)	***Notoedres cati***
18b	Ânus terminal (Figura 3P; seta); superfície dorsal do corpo recoberta numerosos por espinhos triangulares organizados em fileiras	***Sarcoptes scabiei***
19a	Não ornamentado; *basis capituli* hexagonal; coxa I bífida	***Rhipicephalus sanguineus* s.l.** [c]
19b	Usualmente ornamentado; *basis capituli* com formas variadas	***Amblyomma* spp.** [d]

[a] *Demodex canis* em cães e *Demodex cati* em gatos.

[b] Não existe dados confiáveis sobre *Cheyletiella* spp. em cães e gatos no Brasil.

[c] Pelo menos duas populações biologicamente e geneticamente distintas sob o nome "*R. sanguineus*" foram encontradas em cães na América do Sul [52, 161, 162].

[d] Várias espécies relatadas em cães e gatos. *Amblyomma aureolatum*, *Amblyomma cajennense* e *Amblyomma ovale* estão entre as espécies de *Amblyomma* encontradas em cães de áreas rurais no Brasil [55].

Tabela 4 - Endoparasitos de cães no Brasil.

Filo	Classe	Ordem	Família	Espécie[a]
Apicomplexa	Aconoidasida	Piroplasmida	Babesiidae	*Babesia gibsoni*
				Babesia vogeli
			Theileriidae[b]	*Rangelia vitalii*
	Conoidasida	Eucoccidiorida	Hepatozoidae	*Hepatozoon canis*
			Cryptosporidiidae	*Cryptosporidium canis*
			Eimeridae	*Isospora canis* (sin. *Cystoisospora canis*)
				Isospora ohioensis (sin. *Cystoisospora ohioensis*)
			Sarcocystidae	*Hammondia heydorni*
				Neospora caninum
				Sarcocystis capracanis
				Sarcocystis cruzi (sin. *Sarcocystis bovicanis*)
				Sarcocystis levinei
				Sarcocystis miescheriana (sin. *Sarcocystis suicanis*)
				Sarcocystis tenella (sin. *Sarcocystis ovicanis*)
Metamonada	Parabasalia	Trichomonadida	Trichomonadidae	*Pentatrichomonas hominis* (sin. *Trichomonas hominis*)
Sarcomastigophora	Zoomastigophora	Diplomonadida	Hexamitidae	*Giardia duodenalis* (sin. *Giardia intestinalis*)
		Kinetoplastida	Trypanosomatidae	*Leishmania infantum* (sin. *Leishmania chagasi*)
				Leishmania amazonensis
				Leishmania braziliensis
				Trypanosoma caninum
				Trypanosoma evansi
				Trypanosoma cruzi
Nematoda	Secernentea	Strongylida	Ancylostomatidae	*Ancylostoma braziliense*
				Ancylostoma caninum
			Angiostrongylidae	*Angiostrongylus vasorum*
		Rhabditida	Strongyloididae	*Strongyloides stercoralis*
		Ascaridida	Ascarididae	*Lagochilascaris minor*
				Toxascaris leonina
				Toxocara canis
			Dioctophymatidae	*Dioctophyme renale*
		Trichurida	Trichinellidae	*Calodium hepaticum* (sin. *Capillaria hepatica*)
			Trichuridae	*Trichuris vulpis*
		Spirurida	Gongylonematidae	*Gongylonema pulchrum*
			Onchocercidae	*Acanthocheilonema reconditum*
				Cercopithifilaria bainae
				Cercopithifilaria grassii (sin. *Dipetalonema grassii*)

				Dirofilaria immitis
			Physalopteridae	*Physaloptera praeputialis*
			Spirocercidae	*Spirocerca lupi*
Platyhelminthes	Cestoda	Cyclophyllidea	Dipylidiidae	*Dipylidium caninum*
			Taeniidae	*Echinococcus granulosus*
				Taenia hydatigena
				Taenia multiceps (sin. *Multiceps multiceps*)
	Trematoda	Opisthorchiida	Heterophyidae	*Ascocotyle longa* (sin. *Phagicola arnaldoi*)
Acanthocephala	Archiacanthocephala	Oligacanthorhynchida	Oligacanthorhynchidae	*Oncicola canis*

[a] Lista de endoparasitos (protozoários e helmintos) que afetam cães no Brasil [32,67-130,344-353]. Achados duvidosos (e.g., *Phagicola minuta*, *Aelurostrongylus abstrusus*, *Dirofilaria repens*, *Necator americanus*, *Trichuris serrata*, *Uncinaria stenocephala*, *Isospora rivolta* e *Isospora bigemina*) [68,69] não foram incluídos.

[b] O gênero *Rangelia* foi considerado por muito tempo como sinônimo de *Babesia* (família Babesiidae), mas datos genéticos e biológicos suportam a sua validade e a sua inclusão na família Theileriidae. A criação de uma família mono-específica (Rangeliidae) não parece desejável.

Tabela 5 - Endoparasitos de gatos no Brasil.

Filo	Classe	Ordem	Família	Espécie[a]
Sarcomastigophora	Zoomastigophora	Diplomonadida	Hexamitidae	*Giardia duodenalis*
		Kinetoplastida	Trypanosomatidae	*Leishmania infantum*
				Leishmania amazonensis
				Leishmania braziliensis
				Trypanosoma cruzi
Apicomplexa	Aconoidasida	Piroplasmida	Babesiidae	*Babesia vogeli*
			Theileriidae	*Cytauxzoon felis*
	Conoidasida	Eucoccidiorida	Hepatozoidae	*Hepatozoon canis*
				Hepatozoon felis
			Cryptosporidiidae	*Cryptosporidium felis*
			Eimeriidae	*Isospora felis* (sin. *Cystoisospora felis*)
				Isospora felis (sin. *Cystoisospora rivolta*)
			Sarcocystidae	*Hammondia hammondi*
				Sarcocystis hirsuta (sin. *Sarcocystis bovifelis*)
				Toxoplasma gondii
Nematoda	Secernentea	Strongylida	Ancylostomatidae	*Ancylostoma braziliense*
				Ancylostoma caninum
				Ancylostoma tubaeforme
			Angiostrongylidae	*Aelurostrongylus abstrusus*
				Gurltia paralysans
			Syngamidae	*Mammomonogamus dispar*
		Rhabditida	Strongyloididae	*Strongyloides stercoralis*
		Ascaridida	Ascarididae	*Lagochilascaris minor*
				Toxascaris leonina
				Toxocara cati (sin. *Toxocara mistax*)
			Dioctophymatidae	*Dioctophyme renale*
		Trichurida	Capillariidae	*Calodium hepaticum* (sin. *Capillaria hepatica*)
				Pearsonema feliscati
			Trichuridae	*Trichuris campanula*
				Trichuris serrata
		Spirurida	Onchocercidae	*Dirofilaria immitis*
			Physalopteridae	*Physaloptera praeputialis*
Platyhelminthes	Cestoda	Cyclophyllidea	Dipylidiidae	*Dipylidium caninum*
			Taeniidae	*Taenia taeniformis*
		Pseudophyllidea	Diphyllobothriidae	*Spirometra mansonoides*
	Trematoda	Opisthorchiida	Heterophyidae	*Ascocotyle angrense*

				Ascocotyle longa (sin. *Phagicola arnaldoi*)
		Plagiorchiida	Dicrocoeliidae	*Platynosomum fastosum*
		Strigeatida	Diplostomatidae	*Alaria alata*
Acanthocephala	Archiacanthocephala	Moniliformida	Moniliformidae	*Moniliformis moniliformis*
	Palaeacanthocephala	Polymorphida	Centrorhynchidae	*Sphaerirostris erraticus*

[a] Lista de endoparasitos (protozoários e helmintos) que afetam gatos no Brasil [42,44,47,71,87,90,92,97,129,131-148,346,349,352-356]. Achados duvidosos (e.g., *Ascaridia galli* e *Heterakis gallinarum*) [68,69] não foram incluídos.

doi:10.1186/1756-3305-7-22
Cite this article as: Dantas-Torres and Otranto: **Dogs, cats, parasites, and humans in Brazil: opening the black box.** Parasites & Vectors 2014 7:22.
